# A Pan-Cancer Analysis of the Oncogenic Role of Cell Division Cycle-Associated Protein 4 (CDCA4) in Human Tumors

**DOI:** 10.3389/fimmu.2022.826337

**Published:** 2022-02-17

**Authors:** Hui Fang, Shuyan Sheng, Bangjie Chen, Jianpeng Wang, Deshen Mao, Yanxun Han, Yuchen Liu, Xinyi Wang, Siyu Gui, Tongyuan Zhang, Lizhi Zhang, Conghan Li, Xinyang Hu, Wanyu Deng, Xin Liu, Honghai Xu, Wentao Xu, Xingyu Wang, Rongqiang Liu, Weihao Kong

**Affiliations:** ^1^ Department of Pharmacy, Affiliated Hospital of Hangzhou Normal University, Hangzhou, China; ^2^ The First Clinical Medical College of Anhui Medical University, Hefei, China; ^3^ Department of Oncology, The First Affiliated Hospital of Anhui Medical University, Hefei, China; ^4^ Department of Otolaryngology Head and Neck surgery, The First Affiliated Hospital of Anhui Medical University, Hefei, China; ^5^ Department of Ophthalmology, The Second Affiliated Hospital of Anhui Medical University, Hefei, China; ^6^ Department of Emergency Surgery, The First Affiliated Hospital of Anhui Medical University, Hefei, China; ^7^ School of Pharmacy, Anhui Medical University, Hefei, China; ^8^ Department of Pathology, The First Affiliated Hospital of Anhui Medical University, Hefei, China; ^9^ Department of Hepatobiliary Surgery, First Affiliated Hospital of Guangzhou Medical University, Guangzhou, China

**Keywords:** CDCA4, pan-cancer analysis, tumor, HCC, molecular biology experiments

## Abstract

**Purpose:**

To unravel the oncogenic role of CDCA4 in different cancers from the perspective of tumor immunity.

**Methods:**

Raw data on CDCA4 expression in tumor samples and paracancerous samples were obtained from TCGA and GTEX databases. In addition, we investigated pathological stages and the survival analysis of CDCA4 in pan-cancer across Gene Expression Profiling Interactive Analysis (GEPIA) database. Cox Proportional Hazards Model shows that high CDCA4 levels are associated with several vital indicators in oncology. On the one hand, we explored the correlation between CADA4 expression and tumor immune infiltration by the TIMER tool; On the other hand, we utilized the methods of CIBERSORT and ESTIMATE computational to evaluate the proportion of tumor infiltrating immune cells (TIIC) and the amounts of stromal and immune components based on TCGA database. The use of antineoplastic drugs and the expression of CDCA4 also showed a high correlation *via* linear regression. Protein–Protein Interaction analysis was performed in the GeneMANIA database, and enrichment analysis was performed and predicted signaling pathways were identified by using Gene Ontology and Kyoto Encyclopedia of Genes. The correlation between CDCA4 expression with Copy number variations (CNV) and methylation is detailed, respectively. Molecular biology experiments including Western blotting, flow cytometry, EDU staining, Transwell and Wound Healing assay to validate the cancer promoting role of CDCA4 in hepatocellular carcinoma (HCC).

**Results:**

Most tumors highly expressed CDCA4. Elevated CDCA4 expression was associated with poor OS and DFS. There was a significant correlation between CDCA4 expression and TITCs. Moreover, markers of TIICs exhibited distinct patterns of CDCA4 associated immune infiltration. In addition, we pay attention to the association between the expression of CDCA4 and the use of the anti-tumor drugs. CDCA4 is related to biological progress (BP), cellular component (CC) and molecular function (MF). Dopaminergic Synapse, AMPK, Sphingolipid, Chagas Disease, mRNA Surveillance were significantly enriched pathways in positive and negative correlation genes with CDCA4. CNV is thought to be a positive correlation with CDCA4 expression. Conversely, methylation is negative correlation with CDCA4 expression. Molecular biology experiments confirm a cancer promoting role for CDCA4 in HCC

**Conclusion:**

CDCA4 may serve as a biomarker for cancer immunologic infiltration and poor prognosis, providing a new way of thinking for cancer treatment.

## Introduction

CDCA4 (Cell division cycle-associated protein 4), also known as HEPP/SEI3/TRIPBr3, was discovered to be a gene expressed only in hematopoietic progenitor cells rather than hematopoietic stem cells (1). The chenodeoxycholic acid (CDCA) family genes were shown to be tightly co-expressed with known cell cycle genes such as CDC2 (cell division cycle 2 gene), CDC7 (cell division cycle 7 gene), and cyclins, according to gene-based research (2). CDCA4 has been studied from the perspectives of physiology and clinical pathology in several species, and it was thought to have a critical role in cell cycle regulation (3). There was a plethora of cell or animal experiment-based data linking CDCA4 to several forms of malignancies. CDCA4 was shown to be upregulated in breast cancer (3), non-small cell lung cancer (4), osteosarcoma (5), head and neck squamous cell carcinoma (6), and ovarian cancer (6, 7). CDCA4 is now a potential biomarker and a key mediator in a variety of human malignancies, although the link between CDCA4 function and carcinogenesis remains unclear.

Despite the fact that numerous oncogenes and tumor suppressor genes have been found and linked to signaling pathways that govern cell growth or death in order to produce anti-tumor agents and therapeutic techniques, cancer treatment has hit a roadblock (8). Due to its capacity to lead to a paradigm change in the treatment of many advanced cancers, immunotherapy is seen as a route out of the bottleneck. Combination treatments employing metabolic inhibitors in conjunction with immune checkpoint blockade (ICB), chemotherapy, and radiation are increasingly being explored as potential cancer treatment options. The tumor microenvironment (TME), according to a new theory, plays a critical role in the beginning and progression of human malignancies (8–10). However, given the setting of the complicated TME, it is unclear how to appropriately use these tactics (11). Oncogene-driven alterations in tumor cell metabolism might affect the TME, limiting immune responses and posing hurdles to cancer treatment, implying that the TME is one of the most important elements influencing immunotherapy success. In order to better understand the dynamic regulatory mechanism of matrix and immunological components in TME, further research is required. As a result, elucidating the immunophenotypes of tumor-immune interactions and validating novel immune-related therapeutic targets in malignancies is critical.

Based on several databases, we investigated the expression of CDCA4 and its association with tumor infiltrating immune cells (TIICs) and associated immunological markers, as well as the prognosis of multiple cancers. Our data imply that CDCA4 interacts with TIICs to alter cancer patient prognosis. CDCA4 has a carcinogenic impact on pan-cancer, and raising CDCA4 expression may decrease human cancer patient survival time. Moreover, we carried out molecular biology verification in HCC to further confirm the cancer promoting role of CDCA4. In conclusion, CDCA4 is a prospective and promising therapeutic target for cancer, as well as a marker of immune infiltration and poor prognosis.

## Materials and Methods

### Data Collection and Processing

Pan-cancer sequencing data from The Cancer Genome Atlas (TCGA) and Broad Institute Cancer Cell Line Encyclopedia (CCLE), as well as data linked to liver hepatocellular carcinoma (LIHC) from the International Cancer Genome Consortium (ICGC), were extracted for analysis through their portal websites (12–14). Using the rma function in the R package (R studio version: 1.2.1335, R version: 3.6.1) (http://www.r-project.org/ https://www.rstudio.com/), the whole data set was filtered, deleting missing and duplicated results, and transformed by log2(TPM +1). Patients’ age, sex, tumor stages, and clinical stages were all retrieved from the portal websites, along with other clinical data. Furthermore, tumor mutation burden (TMB) and microsatellite instability (MSI) were downloaded data that was only available from the TCGA database. TMB was determined by counting the number of insertion or deletion events in repetitive gene sequences, whereas MSI was determined by counting the overall mutation occurrences per million base pair.

### Survival Analysis and Relationship With Clinical Stage

The Gene Expression Profiling Interactive Analysis (GEPIA) database (http://gepia.cancer-pku.cn) (15) is an online platform that uses a common processing technique to examine RNA sequencing expression data from the TCGA and The Genotype-Tissue Expression (GTEx) projects. The GEPIA “Survival” module was used to examine the relationship between CDCA4 expression and cancer prognosis. GEPIA also has interactive features including profiling based on pathological stages (15).

### Cox Regression Analysis and Survival Analysis

In the R environment, Cox regression analysis was used to look at the relationship between CDCA4 expression and patients’ overall survival (OS) and disease free survival (DFS) in each cancer type using the TCGA databases. After separating patients into high and low CDCA4 expression groups through the best separation method, the Kaplan–Meier method was used to create the survival curves of patients in each cancer type. The survival were investigated using survival ROC and survival in the R package (rdocumentation.org/packages/survival) (16). The difference between curves was examined using the log-rank test, with a P-value of less than 0.05 considered significant.

### Immune Cell Infiltration Enrichment

Tumor Immune Estimation Resource (TIMER) is a database-driven web application that calculates immune cell infiltration scores for six main immune cell types, including B cells, CD4+, T cells, CD 8+, T cells, macrophages, neutrophils, and dendritic cells (17, 18). TIMER has previously computed and saved the immune cell infiltration scores of pan-cancer data from the TCGA database. The infiltration data were retrieved and examined to see if there was a link between CDCA4 expression and infiltration.

### CDCA4 and Drug Response

CellMiner (http://discover.nci.nih.gov/cellminer/) suggested a link between CDCA4 expression and drug response. CellMiner is generated by the Genomic and Pharmacology Facilit, DTB, CCR, NCI, NIH. It is a database and query tool designed for the cancer research community to facilitate integration and study of molecular and pharmacological data for the NCI-60 cancerous cell lines. The NCI-60, a panel of 60 diverse human cancer cell lines used by the Developmental Therapeutics Program of the U.S. National Cancer Institute to screen over 100,000 chemical compounds and natural products (since 1990).

### Protein–Protein Interaction Network Construction

GeneMANIA (http://www.genemania.org) is an interactive and user-friendly website for building a protein-protein interaction (PPI) network, which provides gene function prediction hypotheses and identifies genes with comparable roles (19, 20). Physical interaction, co-expression, colocalization, gene enrichment analysis, genetic interaction, and website prediction are among the bioinformatics approaches used in this network integration algorithm. GeneMANIA was used to analyze CDCA4 PPI in this investigation.

### CDCA4-Related Gene Enrichment Analysis

We used a single protein name (“CDCA4”) and organism (“Homo sapiens”) to search the STRING database (https://string-db.org/). Following that, we set the following main parameters: the minimum required interaction score [“Low confidence (0.150)”], the meaning of network edges (“evidence”), the maximum number of interactors to display (“no more than 50 interactors” in the first shell), and active interaction sources (“experiments”). Finally, the CDCA4-binding proteins that have been experimentally determined were retrieved. Furthermore, we integrated the two sets of data to undertake KEGG pathway analysis (Kyoto encyclopedia of genes and genomes). We collected the data for the functional annotation chart by uploading the gene lists to DAVID (Database for annotation, visualization, and integrated discovery) with the parameters of chosen identifier (“OFFICIAL_GENE_SYMBOL”) and species (“Homo sapiens”). Finally, the enriched pathways were displayed using the R packages “tidyr” and “ggplot2.” In addition, we used the R package “clusterProfiler” to run GO.

### CDCA4 CNV Profile in Pan-Cancer Based on GSCA

Gene Set Cancer Analysis (GSCA) (21) platform is a web server that integrates multiomics data based on the TCGA database (http://bioinfo.life.hust.edu.cn/web/GSCA/). Copy number variation (CNV), methylation, pathway activity, and immunological infiltrates are among the studies offered by GSCA. Correlation between mRNA expression quantity of CDCA4 and CNV in different tumors analyzed by GSCA website.

### CDCA4 Methylation Profile in Pan-Cancer Based on GSCA

The correlation between the amount of mRNA expression and the degree of methylation of CDCA4 in different tumors was analyzed by GSCA website.

### Specimen Collection

The liver tissues were collected from patients undergoing surgery at The First Affiliated Hospital of Anhui Medical University. All patients received a histopathological diagnosis of HCC based on World Health Organization criteria. The tissues were immediately snap-frozen in liquid nitrogen after surgical removal and stored at −80°C. The study was complied with the standards approved by the Ethics Committee of Health Medical Research of Anhui Medical University which was in accord with the Helsinki Declaration. All participants signed patients’ informed consent.

### Cell Lines and Cell Culture

Human HCC cell lines (HepG2 and Huh-7 cells) were obtained from the Center for Excellence in Molecular Cell Science (Shanghai, China). All cells were stored in liquid nitrogen and cultured in modified Eagle’s Medium (DMEM, Gibco BRL, USA) supplemented with 1% antibiotics (100 U/ml penicillin and 100 ug/ml streptomycin sulfates, Sigma, USA) and 10% heat-inactivated fetal bovine serum (FBS, Gibco, USA) in a humidified incubator (5% CO2 at 37°C).

### Plasmid Construction and Cell Transfection

Overexpression plasmid of CDCA4 (PEX-3-CDCA4) was constructed and stored in our laboratory (Hefei, China). The PEX-3 vector was used as an internal control. PEX-3-CDCA4 and PEX-3 were transfected in human HCC cells by applying LipofectamineTM2000 according to the manufacturer’s protocol.

### RNA Interference Analysis

Small interfering RNA (siRNA) oligonucleotides targeting CDCA4 genes and scramble siRNA were designed and synthesized by the Gene Pharma Corporation (Shanghai, China) and contained the following sequences:

CDCA4-siRNA: F:5′-GGUGUGUUUUCUUUUGUGCTT-3′, R:5′-GCACAAAAGAAAACACACCTT-3′

Negative control siRNA: F: 5′-UUCUCCGAACGUGUCACGUTT-3′, R: 5′-ACGUGACACGUUCGGAGAA TT-3′.

Briefly, Human HCC cells (HepG2 and Huh-7 cells) in the logarithmic phase were inoculated into 6-well plates with antibiotic-free DMEM. The cell density per well was 6×10^5^ cells. LipofectamineTM2000 kit was used for transfection. Immediately, cells were cultured in a humidified incubator (5% CO2 at 37°C) for 24h and then collected.

### Immunohistochemistry (IHC)

After fixation, decalcification, dehydration, transparency, paraffin embedding, dewaxing and dehydration, human HCC slides were infiltrated in the preheated cell transparent liquid including PBS, Triton and 30% Hydrogen Peroxide (H2O2) for 30 min. Then, human HCC slides were incubated in citric acid buffer for 15 min for antigen retrieval. After incubating with 0.3% H2O2 and blocking with 5% goat serum, the slides were incubated with rabbit polyclonal antibody against CDCA4 (1:100, Abcam, UK) overnight. Results were visualized by 3,3’-diaminobenzidine tetrahydrochloride (DBA) staining. Subsequently, the slides were redyed with hematoxylin for 5 min. After washed, dehydrated, transparent and fixed with a gel, the microscope was utilized to detect the immune complexes. All experiments were performed in triplicate.

### Western Blotting Analysis

Total proteins were extracted by using radioimmunoprecipitation assay buffer reagent (RIPA, Beyotime, China) and phenylmethanesulfonyl fluoride (PMSF, Sigma, USA). A Bicinchoninic acid (BCA) protein assay kit (Beyotime, China) was used to measure the protein concentrations. Proteins from each sample were separated by 10% sodium dodecyl sulfate polyacrylamide gel electrophoresis (SDS-PAGE) and then transferred onto the Polyvinylidene fluoride (PVDF) membrane (Millipore, USA). The lysates of tissues or cells with equal weight were separated as aforementioned. After blocking in the 5% skim milk with tris buffered saline tween (TBST, Boster, China), the transferred membranes were incubated in the primary antibodies at 4°C overnight and then washed by TBST (TBS+Tween) for three times for 15 minutes each. Subsequently, the transferred membranes were incubated with secondary antibodies at RT for 1h. After extensive washing in TBST, immunoblotting was visualized using the enhanced chemiluminescence (ECL) development kit (Thermo Scientific, USA) and analyzed with Quantity-one software (Bio-Rad, USA). Antibodies used: rabbit anti-MMP-2, rabbit anti-MMP-9, rabbit anti-Bax and rabbit anti-Bcl-2 were purchased from Abcam UK.

### Flow Cytometry Assay

For cell cycle assay, cells were first harvested by trypsinization and then fixed in 75% ice-cold ethanol in phosphate-buffered saline. The cells were added Bovine pancreatic RNase (2 μg/ml, Sigma) and propidium iodide (10 μg/ml, Invitrogen). And then, the cells were incubated for 30 min at RT and protected from light meanwhile. The flow cytometer (BD biosciences, NJ, USA) was used to analyze the cell distribution. For cell apoptosis assay, specific operating procedures refer to the manufacturer’sprotocols. Cell apoptosis was then measured by using the flow cytometer (BD biosciences, NJ, USA). In graph, the four quadrant respectively stands for necrotic cells, viable cells, early stage apoptotic cells and late stage apoptotic cells.

### Transwell Assay

Human HepG2 and Huh-7 cells were inoculated into the upper chamber with a serum-free medium. Each well was a density of 2 × 10^6^ cells. The bottom chamber is filled with 500 µl of 20% fetal bovine serum (FBS) culture medium. After incubating in a 5% (v/v) CO2 incubator at RT for 2 d, removing the non-invasive cells and matrigel in the upper chamber, and then the cells were fixed on the lower surface with 10% neutral buffered formalin solution and 0.1% crystal violet staining. Count invading cells in five randomly selected microscope fields.

### Wound Healing Assay

Cells in the logarithmic growth phase at passages 3-5 were treated with 1×10^6^/ml cell number were seeded in six well plates. The plates were incubated in the incubator for 24 h, and cells were selected when they reached ~ 70% confluency. Cells were treated with 10μL autoclaved pipette tip on the bottom surface of the adherent cells and gently make a horizontal scratch. The cells were gently washed 2 times with phosphate buffered saline (PBS) to remove detached cells. The plates were incubated in an incubator for 24 h. Cells were fixed using methanol solution. Crystal violet staining. The cell scratch healing was observed using an inverted microscope and photographed and recorded

### Ethynyl-2-Deoxyuridine (EdU) Incorporation Assay

An EdU Apollo DNA *in vitro* kit (RIBOBIO, Guangzhou, China) was used to determine the cell proliferation. The entire operation process followed the manufacturer’s instructions. Exponentially growing human HepG2 and Huh-7 cells were placed on 13 mm glass coverslips, respectively. Then, CDCA4-siRNA and the negative control (NC) were transfected into human HCC cells with LipofectamineTM2000, respectively. After cultured 24 h, the cells were added to 50 μM EdU labeling media and then incubated for 2 hours at RT under 5% CO2. Subsequently, cells were treated with 4% paraformaldehyde, glycine, 1% Triton X-100. Apollo and Hoechst staining were performed for 30 min respectively. The images were taken by fluorescence microscopy (Olympus, Tokyo, Japan).

### Statistics Analysis

For bioinformatic validation, The link between CDCA4 expression and targets of interest, such as immune cell infiltration scores (as mentioned in the preceding section for six immune cell types), TMB, MSI, mismatch repair (MMR) genes, and methylation transferase genes, was assessed using the Spearman Correlation test. Depending on whether the samples were paired or not, paired t tests or the t test were used to compare CDCA4 expression levels across groups or between tumor and normal tissues. Significant was defined as a P-value of less than 0.05. The R tools ggplot2 and forestplot were used to create all of the graphs.

For molecular biology verification, all statistical calculations were performed using the Statistical Product and Service Solutions (SPSS) software. A two-tailed student’s t-test was used to evaluate differences between two groups. One-factor Analysis of Variance (One-way ANOVA) analyses of variance was used to calculate statistical significance more than two groups. Data are reported as means ± SD. Differences were defined as statistically significance if P value <0.05.

## Result

### CDCA4 Expression Levels Across Various Normal and Cancer Tissues

**Figure 1:**
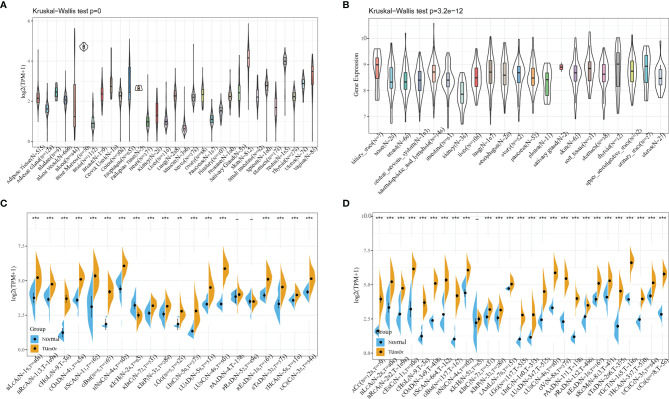
**(A)** Using data from the GTEx database, mRNA expression levels of CDCA4 in multiple organs of healthy persons; **(B)** From the data of CCLE database, CDCA4 expression levels are not only higher universally in diverse cancer cell lines, but also in tighter ranges when compared to the range of expression in normal tissues; **(C)** Taking TCGA data alone, CDCA4 expression differences between tumor and normal tissues for 20 cancers; **(D)** Combining the data from TCGA and GTEx, CDCA4 expression differences between tumor and normal tissues for 27 cancers. (*** represents p < 0.001).

The mRNA expression levels of CDCA4 were shown to be comparable across all organs (**Figure 1A**), with the exception of bone marrow, using data from the GTEx database, which included diverse tissues from healthy persons. More importantly, CDCA4 expression levels are not only higher universally in diverse cancer cell lines from the cancer cell line encyclopedia (CCLE) database, but also in tighter ranges when compared to the range of expression in normal tissues (**Figure 1B**). A study of relatively normal tissues and cancers revealed that CDCA4 was strongly expressed in most tumors, with the exception of the kidney chromophobe (KICH), which revealed the opposite finding with significance. Taking TCGA data alone, in 18 of the 20 cancer types studied, the expression difference was significant (the exceptions were prostate adenocarcinoma (PRAD) and pancreatic adenocarcinoma (PAAD), while differences were significant for 26 of the 27 cancers when the data from TCGA and GTEx were combined. The expression levels of tumor and normal tissues were comparable in the kidney renal clear cell carcinoma (KIRC), however CDCA4 expression was lower in the KICH when compared to normal tissues (**Figures 1C, D**).

### CDCA4 Expression Was Positively Correlated With Advanced Stages of Cancers

**Figure 2:**
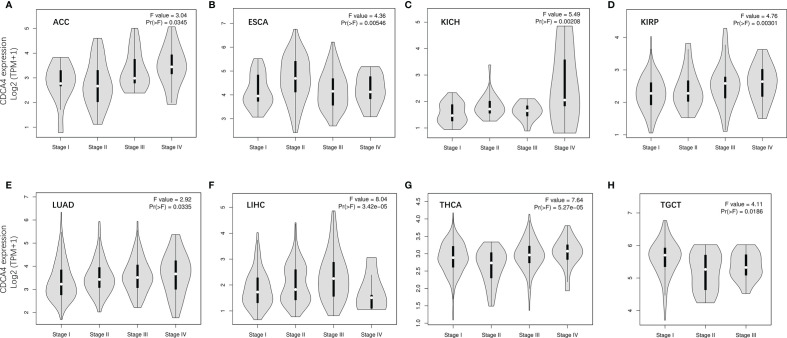
The correlation between CDCA4 expression and the pathological stages of cancers, including ACC **(A)**, ESCA **(B)**, KICH **(C)**, KIRP **(D)**, LUAD **(E)**, LIHC **(F)**, THCA **(G)**, TGCT **(H)** using HEPIA2’s “Pathological Stage Plot” module.

We used GEPIA2’s “Pathological Stage Plot” module to examine the correlation between CDCA4 expression and the pathological stages of cancers. Adrenocortical carcinoma (ACC), Esophageal carcinoma (ESCA), KICH, Kidney renal papillary cell carcinoma (KIRP), Lung adenocarcinoma (LUAD), LIHC, Thyroid carcinoma (THCA), Testicular Germ Cell Tumors (TGCT) were all significantly associated with tumor stage (**Figure 2**, all P<0.05).

### Analysis of Link Between CDCA4 Expression Level and Prognosis

**Figure 3:**
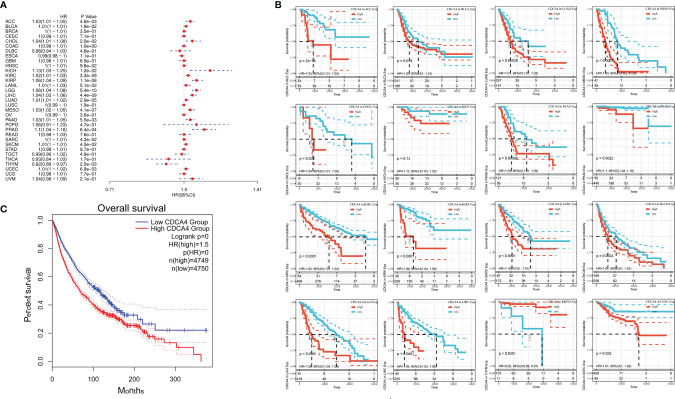
**(A)** The relationship between CDCA4 expression levels and OS in various cancer types through single variate Cox regression analysis using data from the TCGA database; **(B)** The survival analyses for median expression value in various cancer types using data from the TCGA database; **(C)** A high CDCA4 expression level was associated with a poorer OS when compared to a low expression level in the 16 cancer types (HR = 1.5) using data from the TCGA database.

Using data from the TCGA database, we examined the relationship between CDCA4 expression levels and OS in various cancer types through single variate Cox regression analysis. The hazard ratios for CDCA4 were significant for Pheochromocytoma and Paraganglioma (PCPG), ACC, KIRP, Cholangiocarcinoma (CHOL), KICH, KIRC, Acute Myeloid Leukemia (LAML), Brain Lower Grade Glioma (LGG), Bladder Urothelial Carcinoma (BLCA), LUAD, Mesothelioma (MESO), PAAD, PRAD, Skin Cutaneous Melanoma (SKCM), LIHC and uveal melanoma (UVM), among which CDCA4 had the highest risk effect in KICH (**Figure 3A**). The subsequent survival analyses, which used patient data dichotomized for optimal cut off value in each cancer type (**Figure 3B**), demonstrate that survival differences in OS-related cancer types were all significant, indicating that patients with high CDCA4 expression had poorer outcomes (**Figure 3B**). Following that, a high CDCA4 expression level was associated with a poorer OS in the TCGA cancer types shown in **Figure 3B** (HR = 1.5, **Figure 3C**) when compared to a low expression level.

**Figure 4:**
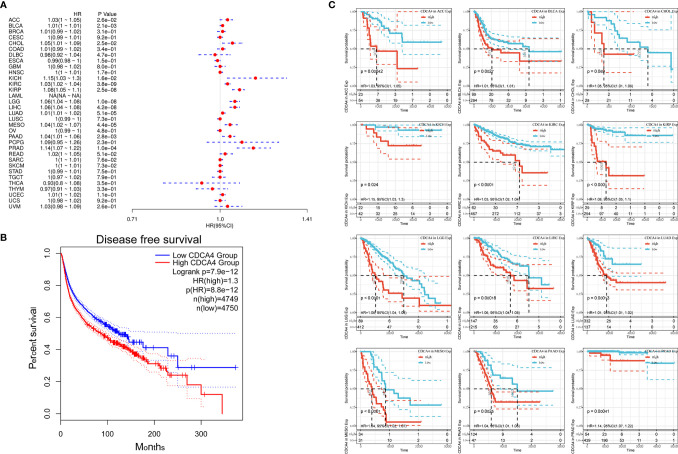
**(A)** Correlation between CDCA4 expression and DFS analyzed by Cox regression using data from the TCGA database; **(B)** The survival analysis for CDCA4 expression and prognosis in various cancer types; **(C)** A high expression level of CDCA4 was concerned to have a correlation with a worse DFS in the 12 cancer types (HR = 1.3) using data from the TCGA database.

Next, correlation between CDCA4 expression and disease free survival (DFS) was also analyzed by Cox regression analysis. The results of the Cox regression analysis gave similar results to those correlating to OS. Differences included identification of a significant risk effect for Breast invasive carcinoma (BRCA), Colon adenocarcinoma (COAD), Uterine Corpus Endometrial Carcinoma (UCEC) (in addition to the formerly mentioned 16 types of cancers). Besides, LAML is lack of the significant risk effect and an inability to calculate a hazard ratio for CDCA4 in LAML due to lack of related data (**Figure 4A**). In the following survival analysis, cancer types with high CDCA4 expression again exhibited a worse prognosis in comparison with the low expression groups (**Figure 4B**). Similarly, a high expression level of CDCA4 was also concerned to have a correlation with a worse DFS in the TCGA cancer types, which are exhibited in **Figure 4B** (HR = 1.3, **Figure 4C**).

### Correlation Analysis of Prognosis

**Figure 5:**
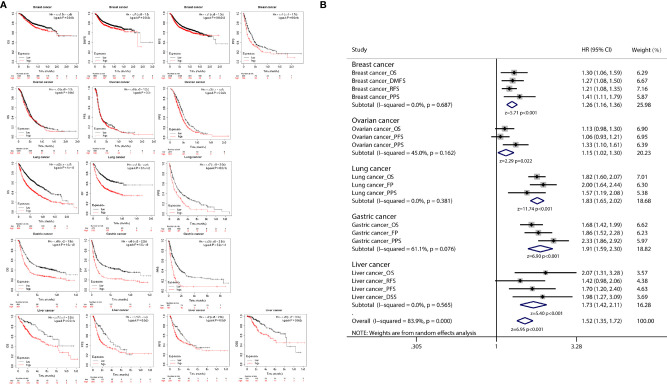
**(A)** K-M plots showed that different CDCA4 levels were associated with pathological stages of OS, PFS, DFS, RFS, DMFS, PSS, DSS and FP events; **(B)** The results of survival analysis of multiple cancers were integrated into the meta-analysis, and the combined results showed that high expression of CDCA4 in cancer patients was significantly associated with poor prognosis.

K-M plot was used to further analyze the correlation between CDCA4 expression and cancer prognosis, and the results of the correlation analysis indicated that CDCA4 expression was associated with the prognosis of various cancer types, including breast, ovarian, lung, gastric and liver cancer (**Figure 5A**). Then we integrated the results of the survival analysis in a meta-analysis, and the integrated results showed that high expression of CDCA4 was significantly associated with poor prognosis in cancer patients (**Figure 5B**).

### Correlation Between CDCA4 Expression and Immune Infiltrating Level in Cancers

**Figure 6:**
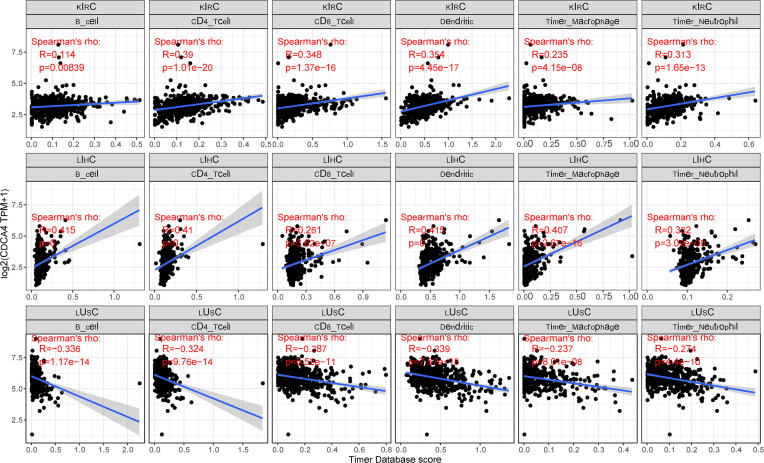
A strong connection between CDCA4 expression and the degree of immune cell infiltration in multiple malignancies using the infiltration scores of six immune cell types (B cell, CD4+ T cell, CD8+ T cell, neutrophil, macrophage, and dendritic cell) accessible in the TIMER database and obtained from TCGA.

CDCA4 is known to contribute to the preservation of the Golgi architecture in tumor cells, suggesting that it may influence immune cell differentiation through the serotonin pathway. To determine if this pathway has an effect on the immunological milieu of tumors, we examined the connection between CDCA4 expression and the degree of immune cell infiltration in each cancer type. We indeed found a strong association in multiple malignancies using the infiltration scores of six immune cell types (B cell, CD4+ T cell, CD8+ T cell, neutrophil, macrophage, and dendritic cell) accessible in the Tumor Immune Estimation Resource (TIMER) database and obtained from TCGA. KIRC, LIHC, and lung squamous cell carcinoma (LUSC) were the top three tumor groups. The accompanying linear regression graphs from KIRC and LIHC indicate that high CDCA4 expression is associated with increased amount of immune cell infiltration. It is worth noting that there is a stronger association between CDCA4 expression and the amount of immune cell infiltration in LIHC. On the contrary, a negative connection exists between CDCA4 expression and immune cell infiltration in LUSC. It is worth mentioning that dendritic cells exhibited the greatest significant coefficients of all cell types in all three malignancies. Additionally, B cells are expected to have the highest coefficients in LIHC (**Figure 6**).

**Figure 7:**
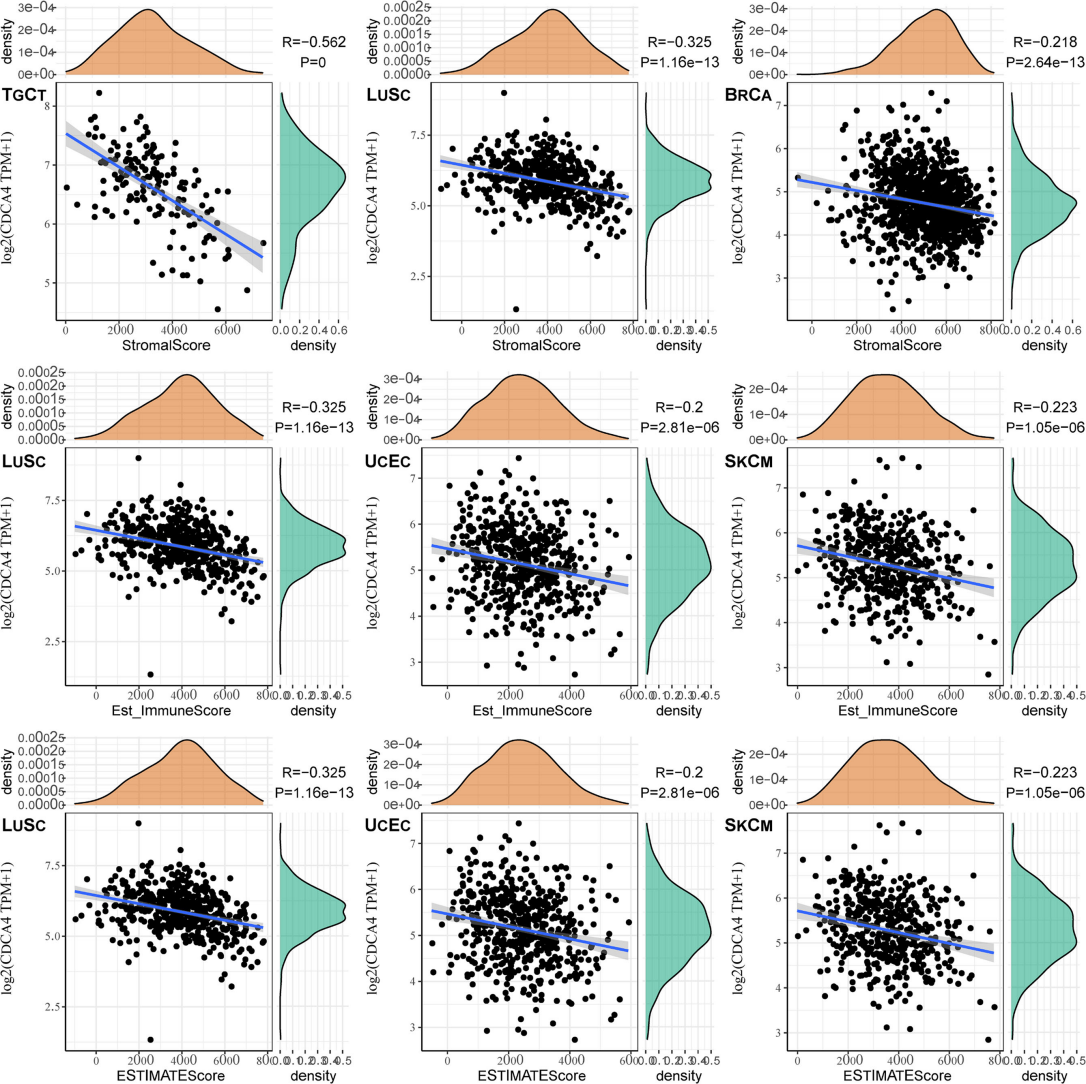
The top three tumors with the most significant correlation between the degree of immune infiltration and CDCA4 expression were TGCT, LUSC and BRCA (StromalScore); LUSC, UCEC and SKCM (ImmuneScore); LUSC, UCEC and SKCM (ESTIMATEScore), respectively.

The top three tumors most significantly correlated with expression of CDCA4 wereTGCT, LUSC and BRCA (StromalScore), LUSC, UCEC and SKCM (ImmuneScore), LUSC, UCEC and SKCM (ESTIMATEScore) respectively (**Figure 7**). Therefore, the results indicated that CDCA4 expression was tightly correlated with the extent of immune infiltration in cancers.

### Correlation of CDCA4 Expression With Expression of Some Immune Checkpoint Genes for Certain Cancers Implicates CDCA4 in the Tumor Immune Response

**Figure 8:**
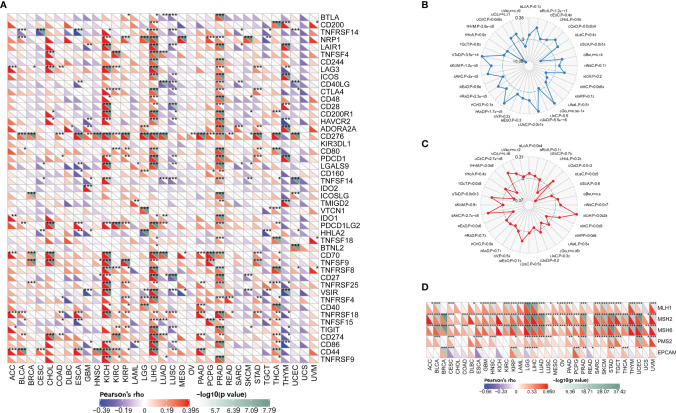
**(A)** CDCA4 expression was inversely linked with most immunological checkpoint molecules in HNSC, LUSC, SARC, SKCM, THYM, UCEC and UCS, though not to a substantial degree in some of them; **(B)** In BRCA, COAD, ESCA, LGG, LUAD, LUSC, PAAD, PRAD, SARC, SKCM, STAD, THYM and UCEC, CDCA4 expression was positively connected with TMB, whereas in ESCA, KIRC, THYM and UCEC, it was adversely correlated with TMB; **(C)** In DLBC and TGCT, CDCA4 expression was inversely connected with MSI, but it was positively correlated in COAD, KICH, MESO, SARC, STAD and UCEC; **(D)** CDCA4 expression considerably and strongly correlates with MMR gene expression in most cancer types (excluding CHOL, COAD, KICH, and READ); MLH1, MSH2, and MSH6 are all positively connected with CDCA4 in most cancer types. (* represents 0.01 < p < 0.05, ** represents 0.001 < p < 0.01, *** represents p < 0.001).

There are now multiple genes that are tightly linked to and recognized as immune response checkpoint components. We were able to determine whether there is a relationship between CDCA4 expression and the expression of such checkpoint genes using TCGA databases. In diverse cancer types, correlation analysis between CDCA4 and checkpoint gene expression indicated a high connection (P<0.05) with TNF-related immune genes including TNFRSF8, TNFRSF14, TNFRSF18, CD70, CD44, and CD276 (B7-H3). Furthermore, considerable co-expression of CDCA4 with more immune checkpoint genes was found in KICH, LIHC, and PRAD. The findings, particularly for KICH, LIHC, and PRAD, suggest that CDCA4 is involved in the regulation of the tumor immune response *via* immune checkpoint activity modulation. It’s also worth noting that CDCA4 expression was inversely linked with most immunological checkpoint molecules in head and Neck squamous cell carcinoma (HNSC), LUSC, Sarcoma (SARC), SKCM, human neural stem cells (THYM), UCEC and Uterine Carcinosarcoma (UCS), though not to a substantial degree in some of them (**Figure 8A**).

### CDCA4 Is Associated With the TMB and MSI in Some Cancers

In BRCA, COAD, ESCA, LGG, LUAD, LUSC, PAAD, PRAD, SARC, SKCM, STAD, THYM and UCEC, CDCA4 expression was positively connected with TMB, whereas in ESCA, KIRC, THYM and UCEC, it was adversely correlated with TMB (**Figure 8B**). In Diffuse Large B-cell Lymphoma (DLBC) and TGCT, CDCA4 expression was inversely connected with MSI, but it was positively correlated in COAD, KICH, MESO, SARC, Stomach adenocarcinoma (STAD) and UCEC (**Figure 8C**).

### CDCA4 Expression Is Strongly Related to MMR Defects in Different Cancers and May Interfere With Methylation After Transcription

Following the discovery of a connection between CDCA4 expression and the mutation markers TMB and MSI, further research into the relationship between CDCA4 expression and carcinogenesis processes, particularly a link with MMR deficiencies, was required. As a result, we looked at the link between CDCA4 expression and a few well-established MMR genes (MLH1, MSH2, MSH6, PMS2 and EPCAM). As a consequence, CDCA4 expression considerably and strongly correlates with MMR gene expression in all thirty-three cancer types (excluding CHOL, COAD, KICH, and READ); MLH1, MSH2, and MSH6 are all positively connected with CDCA4 in majority of these cancer types (See **Figure 8D**).

### CDCA4 and Drug Response

**Figure 9:**
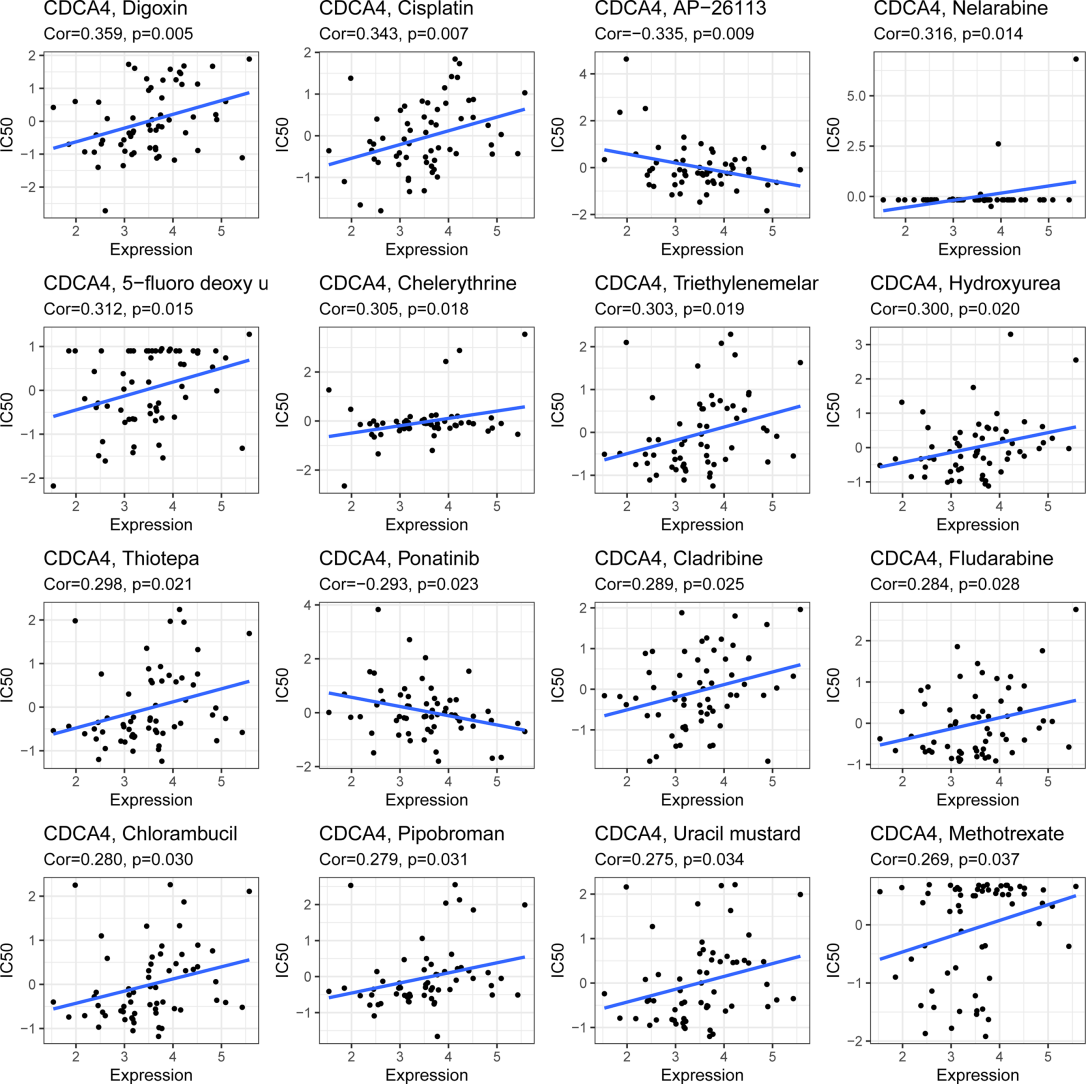
An illustration of the relationship between CDCA4 expression and expected medication response.

CDCA4 expression was positively connected with drug response in patients treated with Digoxin Cisplatin, Nelarabine, 5-fluorodeoxyuridine, Chelerythrine, Triethylenemelar, Hydroxyurea, Thiotepa, Cladribine, Fludarabine, Chlorambucil, Pipobroman, Uracil mustard and Methotrexate. Additionally, there is a negative connection between CDCA4 expression and the anticancer drug AP-26113, Ponatinib. An illustration of the relationship between CDCA4 expression and expected medication response can be found in **Figure 9**.

### PPI Network of CDCA4 in Cancers and Enrichment Analysis

**Figure 10:**
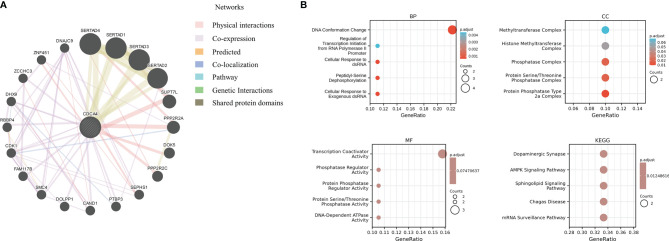
**(A)** a PPI network for CDCA4; **(B)** The biological processes (BP) enriched in this dataset were primarily those related to DNA Conformation Change/Regulation of Transcription Initiation from RNA Polymerase II Promoter/Cellular Response to dsRNA/Peptidyl-Serine Dephosphorylation/Cellular Response to Exogenous dsRNA, while the cellular components (CC) enriched were primarily those related to Phosphatase Complex/Protein Serine/Threonine Phosphatase Complex/Protein Phosphatase Type 2a Complex. Furthermore, the enriched molecular functions (MF) were linked to Transcription Coactivator Activity/Phosphatase Regulator Activity/Protein Phosphatase Regulator Activity/Protein Serine/Threonine Phosphatase Activity/DNA-Dependent ATPase Activity. The major enriched pathways were those connected with Dopaminergic Synapse, AMPK, Sphingolipid, Chagas Disease, and mRNA Surveillance, according to the KEGG analysis.

Next, we utilized the GeneMANIA online program to create a PPI network for CDCA4, which is displayed in **Figure 10A**, to investigate the probable processes by which CDCA4 played a role in cancer carcinogenesis. CDCA4 demonstrated significant physical interactions with SERTAD4, SERTAD1, SERTAD3 and SERTAD2, as illustrated in the figure. The biological processes (BP) enriched in this dataset were primarily those related to DNA Conformation Change/Regulation of Transcription Initiation from RNA Polymerase II Promoter/Cellular Response to dsRNA/Peptidyl-Serine Dephosphorylation/Cellular Response to Exogenous dsRNA, while the cellular components (CC) enriched were primarily those related to Phosphatase Complex/Protein Serine/Threonine Phosphatase Complex/Protein Phosphatase Type 2a Complex. Furthermore, the enriched molecular functions (MF) were linked to Transcription Coactivator Activity/Phosphatase Regulator Activity/Protein Phosphatase Regulator Activity/Protein Serine/Threonine Phosphatase Activity/DNA-Dependent ATPase Activity. The major enriched pathways were those connected with Dopaminergic Synapse, AMPK, Sphingolipid, Chagas Disease, and mRNA Surveillance, according to the KEGG analysis (**Figure 10B**).

### The Positive Correlation Between CDCA4 CNV and mRNA

**Figure 11:**
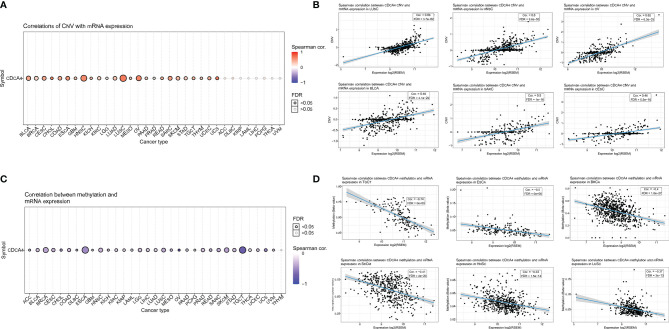
**(A)** A Spearman association between CDCA4 CNV and mRNA was performed in pan-cancer. In BLCA, HNSC, LUSC, OV, and SARC, there is a substantial positive connection between CDCA4 CNV and mRNA expression. On the contrary, this connection was not significant in ACC, DLBC, KIRP, LAML, LIHC, PCPG, THCA and UVM; **(B)** The top six with the highest correlation scores between CDCA4 CNV and mRNA; **(C)** With the exception of THYM, CDCA4 methylation was shown to be strongly linked with CDCA4 mRNA expression in most cancer types. Especially in BRCA, ESCA and TGCT, the relevance is particularly obvious; **(D)** The top six with the highest correlation scores between CDCA4 methylation and mRNA.

In pan-cancer, a Spearman association between CDCA4 CNV and mRNA was performed. In BLCA, Head and Neck squamous cell carcinoma (HNSC), LUSC, Ovarian serous cystadenocarcinoma (OV), and Sarcoma (SARC), there is a substantial positive connection between CDCA4 CNV and mRNA expression. On the contrary, this connection was not significant in Adrenocortical cancer (ACC), Diffuse Large B-cell Lymphoma (DLBC), KIRP, LAML, LIHC, PCPG, THCA, UVM (**Figure 11A**). **Figure 11B** show the top six with the highest correlation scores.

### CDCA4 Methylation Profile in Pan-Cancer Based on GSCA

CDCA4 methylation landscape in pan-cancer was also investigated. With the exception of Thymoma (THYM), CDCA4 methylation was shown to be strongly linked with CDCA4 mRNA expression in most cancer types. Especially in BRCA, ESCA and TGCT, the relevance is particularly obvious (**Figure 11C**). **Figure 11D** show the top six with the highest correlation scores.

### The Expression of CDCA4 in Human HCC

In the study, the expression level of CDCA4 was first analyzed in HCC tissues and adjacent tissues. IHC results showed that the expression level of CDCA4 was up-regulated in HCC tissues compared to the adjacent tissues (**Supplementary Figure S1A**). The results of Western blotting were consistent with IHC (**Supplementary Figure S1B**).

### Effect of CDCA4 on Apoptosis and Proliferation of HCC Cells

To detect the effect of CDCA4 in HCC, PEX-3-CDCA4 and CDCA4-siRNA were transfected respectively in HepG2 and Huh-7 cells to significantly increase and decrease the expression level of CDCA4. The flow cytometry results showed that CDCA4-siRNA significantly promoted HCC cells apoptosis (**Supplementary Figure S2A**). Meanwhile, the flow cytometry discovered that HCC cells transfected with CDCA4-siRNA displayed a larger G0/G1 population and arrested S and G2/M phase (**Supplementary Figure S2B**). EdU staining results showed that CDCA4-siRNA significantly reduced cell proliferation in HepG2 and Huh-7 cells (**Supplementary Figure S2C**). Furthermore, Western blotting result showed that PEX-3-CDCA4 inhibited the expression level of BCL2-associated X protein (Bax) and increased the expression level of B-cell lymphoma-2 (Bcl-2) and PCNA in HCC cells (**Supplementary Figure S2D** and S2E). Interestingly, CDCA4-siRNA remarkably increased the expression level of Bax and inhibited the expression level of Bcl-2 and Proliferating Cell Nuclear Antigen (PCNA) in HCC cells (**Supplementary Figures S2F, G**). All in all, these results confirmed that CDCA4 could inhibited cells apoptosis and promoted cells proliferation in HepG2 and Huh-7 cells.

### Effect of CDCA4 on Migration and Invasion of HCC Cells

To further observe the potential roles of CDCA4 in regulating the ability of HCC cells to invasion and migration. Transwell and Wound Healing results revealed that silence of CDCA4 could inhibit the ability of invasion and migration. However, the ability of invasion and migration was promoted by PEX-3-CDCA4 (**Supplementary Figures S3A, B**). Certainly, the protein expression of MMP-2 and MMP-9 was further verified the previous data (**Supplementary Figures S3C–F**). Collectively, we could conclude that CDCA4 may promote the migration and invasion of HCC cells *in vitro*.

## Discussion

CDCA4 is a nuclear factor activated by the Early 2 factor (E2F) transcription factor family that controls E2F-dependent transcriptional activation and cell proliferation (22). When overexpressed in mammalian cell lines, CDCA4 was found to have the same biological function as the SEI-3 protein, which has recently been shown to interact with CBP (CREB-binding protein) and stimulate the transactivating function of tumor suppressor p53 as well as induce p53-independent growth inhibition (23). CDCA4 differential expression was also shown to be greater in a number of human malignancies, including breast cancer (3), non-small cell lung cancer (4), osteosarcoma (5), HNSC (6), and ovarian cancer (6, 7). CDCA4 has been shown to influence cell proliferation and death in the MCF7/ADM human breast cancer cell line *via* downregulating genes in the Nrf2 signaling pathway (3). Furthermore, studies have also shown that CDCA4 inhibited EMT, migration, and invasion of NSCLC *via* interacting with Coactivator-associated arginine methyltransferase 1 (CARM1) to control autophagy (4).

High CDCA4 expression was predominantly related with cell division and cell cycle activities, according to enrichment analysis. From prometaphase onwards, CDCA4 is involved in spindle organization. CDCA4 may perform a distinct function as a midzone factor engaged in chromosomal segregation or cytokinesis when anaphase starts (24). Uncontrolled growth and invasion were considered as the most dangerous pathological alterations underneath the intricacy and idiopathy of every malignancy (25). CDCA4, Nuclear Mitotic Apparatus (NuMA)-like proteins were identified as important players to interact with microtubules, MAPs (microtubule associated proteins), motors, and other factors involved in the dynamic functions of the spindle at different stages of mitosis, with their symbolic translocation from the nucleus to the mitotic apparatus. CDCA4 is a really new nuclear mitotic apparatus (NuMAP) that has a role in both metaphase spindle structure and anaphase spindle activity. At the moment, the most effective treatment is to stop the cancer cells from reproducing. The prevention of mitotic spindle assembly, which causes cancer cell division and death, is regarded to be the most effective among them (26). The significance of CDCA4 in cycle control is noteworthy. That is why we are concentrating on this gene. CDCA4 is broadly distributed throughout tissues, with a particular high level of expression in bone marrow, according to our findings. It’s probable that high CDCA4 levels are required for high cell proliferation and turnover, which would explain the increased expression in bone marrow. CDCA4 expression was higher in distinct cancer types when compared to matching normal tissues, and this high expression was linked to poorer OS and DFS in those cancer types. KICH was an exception to the norm, since it had much lower amounts of CDCA4.

Under normal conditions, the immune system is capable of recognizing and eliminating tumor cells from the TME. However, tumor cells may use a variety of survival and growth methods, escaping the immune system. Tumor immunotherapy, which includes monoclonal antibody class immune checkpoint inhibitors, cancer vaccines, therapeutic antibodies, and cell treatment, can restore the body’s natural antitumor immune response. TIICs have a clinical effect on the outcome of patients with a variety of malignancies (27). We compiled a list of over 40 frequent immune checkpoint genes and estimated the correlation between their expression and that of CDCA4. In cancer patients, increased expression of PD-1 and PD-L1 by TIICs was associated with a worse prognosis and histological grade (28). CDCA4 expression was shown to correlate favorably with tumor purity but negatively with TIICs. Therefore, it is not hard to understand that CDCA4 overexpression was associated with a poor outcome in KIRC, LIHC, and LUSC. The data suggested that CDCA4 expression was linked with tumor infiltration levels. MSI was related with an increased risk of cancer with particular clinicopathological characteristics, including increased TMB and lymphocytes entering the tumor. Especially, TMB was a latent biomarker for predicting ICB response (29). In addition, Thomas et al. revealed that TMB could predict breast cancer patients’ immune-related survival outcomes (30). Therefore, in the future, on the one hand, we can estimate the effect of immunotherapy by detecting the expression level of CDCA4; On the other hand, we can develop targeted therapy for CDCA4 to combine with traditional immunotherapy to improve its efficacy.

We also concentrate on intracellular signaling regulation and regulatory factor activity, which is regarded to be important (31, 32). The relationships between CNV, methylation, and CDCA4 expression were all investigated in depth. In addition, we also explored the role of CDCA4 in HCC by molecular biological methods. Immunohistochemistry and Western blotting confirmed that CDCA4 expression was up-regulated in HCC tissues. Edu staining confirmed that CDCA4 promoted the proliferation of HCC cells. Flow cytometry sorting revealed that the proportion of cells in G0/G1 phase was elevated after CDCA4 knockdown, confirming the pro proliferative effect of CDCA4. Moreover, flow cytometry also found that CDCA4 was able to inhibit the apoptosis of HCC cells. Transwell assay confirmed that CDCA4 promoted the invasion and migration of HCC cells. These results corroborate the correctness and reliability of the pan cancer bioinformatics analysis results in HCC, and we will perform similar molecular biological validation in more cancers in the future.

However, even though we explored and incorporated information from several databases, there were still some limitations in the present study. To begin with, while the bioinformatic analysis offered us some important insights of CDCA4 in malignancies, and we have also verified the cancer promoting effect of CDCA4 in HCC through molecular biology methods, further biological experiments *in vitro* or *in vivo* are required to validate our results and increase therapeutic usefulness. Moreover, despite the fact that CDCA4 expression was linked to immunity and clinical survival in human malignancies, we were unsure if CDCA4 affected clinical survival *via* the immune route. Overall, our findings revealed the critical involvement of CDCA4 in tumorigenesis and metastasis, as well as a proposed mechanism by which CDCA4 influences tumor immunology, metabolic activity, and Epithelial-Mesenchymal Transition (EMT) in malignancies. Future prospective research concentrating on CDCA4 expression and the tumor immune milieu would be useful in providing a conclusive answer, allowing for the development of an immuno-based anti-cancer therapy.

## Data Availability Statement

The original contributions presented in the study are included in the article/**Supplementary Material**. Further inquiries can be directed to the corresponding authors.

## Ethics Statement

All experiments were performed according to the institutional ethical guidelines for laboratory human care and use of the First Affiliated Hospital of Anhui Medical University. The patients/participants provided their written informed consent to participate in this study.

## Author Contributions

HF, SS, and WK contributed to the conception of the study. BC, JW, DM, and XinW contributed materials and performed the experiment. SG, LZ, CL, TZ, XH, XingW and RL performed the data analyses. YH, YL, WD, XL, HX and WX contributed significantly in writing the manuscript. All authors contributed to the article and approved the submitted version.

## Funding

This study was supported by Youth Training Program of the First Affiliated Hospital of Anhui Medical University (project: 2021kj24), the National Natural ScienceFoundation of China (Youth Program) (81900554), and the Major Program for Supporting Outstanding Talents in Colleges of Ministry of Human Resources and Social Security of the People’s Republic of Anhui (gxyqZD2020013).

## Conflict of Interest

The authors declare that the research was conducted in the absence of any commercial or financial relationships that could be construed as a potential conflict of interest.

## Publisher’s Note

All claims expressed in this article are solely those of the authors and do not necessarily represent those of their affiliated organizations, or those of the publisher, the editors and the reviewers. Any product that may be evaluated in this article, or claim that may be made by its manufacturer, is not guaranteed or endorsed by the publisher.

## Supplementary Material

Supplementary Figure 1
**(A)** IHC results showed that the expression level of CDCA4 was down-regulated in HCC tissues compared to the adjacent tissues; **(B)** The results of Western blotting showed that the expression level of CDCA4 was down-regulated in HCC tissues compared to the adjacent tissues.

Supplementary Figure 2
**(A)** Flow cytometry results showed that CDCA4-siRNA significantly promoted HCC cell apoptosis; **(B)** Flow cytometry results showed that HCC cells transfected with CDCA4-siRNA displayed a larger G0/G1 population and arrested S and G2/M phase; **(C)** Edu staining showed that the CDCA4-siRNA significantly reduced HCC cell proliferation; **(D)** Western blotting results showed that PEX-3-CDCA4 inhibited the expression level of Bax, increased the expression level of Bcl-2 and PNCA in HepG2 cells; **(E)** Western blotting results showed that CDCA4-siRNA remarkably increased the expression level of Bax and inhibited the expression level of Bcl-2 and PNCA in HepG2 cells. **(F)** Western blotting results showed that PEX-3-CDCA4 inhibited the expression level of Bax, increased the expression level of Bcl-2 and PNCA in Huh-7 cells; **(G)** Western blotting results showed that CDCA4-siRNA remarkably increased the expression level of Bax and inhibited the expression level of Bcl-2 and PNCA in Huh-7 cells.

Supplementary Figure 3
**(A)**The results of Transwell analysis indicated that the ability of invasion in HCC cells was enhanced after transfection with PEX-3-CDCA4; **(B)** The results of Wound Healing analysis indicated that the ability of migration in HCC cells was enhanced after transfection with PEX-3-CDCA4. **(C)** Western blotting results showed that CDCA4-siRNA inhibited the expression level of Bax, increased the expression level of MMP2 and MMP9 in HepG2 cells; **(D)** Western blotting results showed that PEX-3-CDCA4 remarkably increased the expression level of Bax and inhibited the expression level of MMP2 and MMP9 in HepG2 cells. **(E)** Western blotting results showed that CDCA4-siRNA inhibited the expression level of Bax, increased the expression level of MMP2 and MMP9 in Huh7 cells; **(F)** Western blotting results showed that PEX-3-CDCA4 remarkably increased the expression level of Bax and inhibited the expression level of MMP2 and MMP9 in Huh7 cells.

## Glossary

**Table:**

ACC	Adrenocortical carcinoma
AMPK	Adenosine 5’-monophosphate (AMP)-activated protein kinase
Bax	BCL2-associated X protein
BCA	Bicinchoninic acid
Bcl-2	B-cell lymphoma-2
BLCA	Bladder Urothelial Carcinoma
BP	Biological progress
BRCA	Breast invasive carcinoma
CARM1	Coactivator-associated arginine methyltransferase 1
CBP	CREB-binding protein
CC	Cellular component
CCLE	Cancer cell line encyclopedia
CDC2	Cell division cycle 2 gene
CDC7	Cell division cycle 7 gene
CDCA	Chenodeoxycholic acid
CHOL	Cholangiocarcinoma
CNV	Copy number variation
COAD	Colon adenocarcinoma
DBA	3,3’-diaminobenzidine tetrahydrochloride
DFS	Disease free survival
DLBC	Diffuse Large B-cell Lymphoma
E2F	Early 2 factor
ECL	Enhanced chemiluminescence
EMT	Epithelial-Mesenchymal Transition
ESCA	Esophageal carcinoma
FBS	Fetal bovine serum
GEPIA	Gene Expression Profiling Interactive Analysis
GSCA	Gene Set Cancer Analysis
GTEx	Genotype-Tissue Expression
H2O2	Hydrogen Peroxide
HCC	Hepatocellular carcinoma
HNSC	Head and Neck squamous cell carcinoma
ICB	Immune checkpoint blockade
ICGC	International Cancer Genome Consortium
IHC	Immunohistochemistry
KICH	Kidney chromophobe
KIRC	Kidney renal clear cell carcinoma
KIRP	Kidney renal papillary cell carcinoma
LAML	Acute Myeloid Leukemia
LGG	Lower Grade Glioma
LIHC	Liver Hepatocellular carcinoma
LUAD	Lung Adenocarcinoma
LUSC	Lung squamous cell carcinoma
MAPs	Microtubule associated proteins
MF	Molecular function
MMR	Mismatch repair
MSI	Microsatellite instability
NC	Negative control
NuMAP	Nuclear mitotic apparatus
One-way ANOVA	One-factor Analysis of Variance
OS	Overall survival
OV	Ovarian serous cystadenocarcinoma
PAAD	Pancreatic Aadenocarcinoma
PBS	Phosphate buffered saline
PCNA	Proliferating Cell Nuclear Antigen
PCPG	Pheochromocytoma and Paraganglioma
PMSF	Phenylmethanesulfonyl fluoride
PPI	Protein-protein interaction
PRAD	Prostate Adenocarcinoma
PVDF	Polyvinylidene fluoride
SARC	Sarcoma
SDS-PAGE	Sodium dodecyl sulfate polyacrylamide gel electrophoresis
SiRNA	Small interfering RNA
SKCM	Skin Cutaneous Melanoma
SPSS	Statistical Product and Service Solutions
STAD	Stomach adenocarcinoma
TBST	TBS+Tween
TCGA	The Cancer Genome Atlas
TGCT	Testicular Germ Cell Tumors
THCA	Thyroid carcinoma
THYM	Thymoma
TIIC	Tumor infiltrating immune cells
TIMER	Tumor Immune Estimation Resource
TMB	Tumor Mutational Burden
TME	Tumor microenvironment
UCEC	Uterine Corpus Endometrial Carcinoma
UCS	Uterine Carcinosarcoma
UVM	Uveal Melanoma
