# Supplementary figures and images for: A Pan-Cancer Analysis of the Oncogenic Role of Cell Division Cycle-Associated Protein 4 (CDCA4) in Human Tumors
Source: Front Immunol. 2022 Feb 17;13:826337. doi: 10.3389/fimmu.2022.826337 (PMC8891459; doi:10.3389/fimmu.2022.826337)

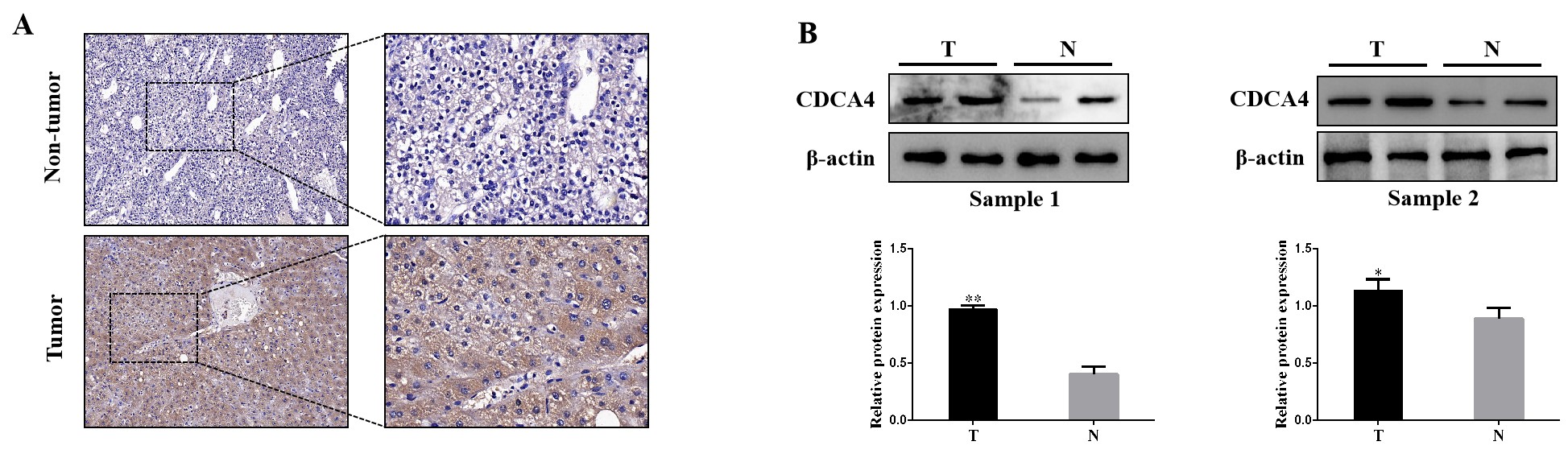

Supplement: Supplementary Figure 1 — (A) IHC results showed that the expression level of CDCA4 was down-regulated in HCC tissues compared to the adjacent tissues; (B) The results of Western blotting showed that the expression level of CDCA4 was down-regulated in HCC tissues compared to the adjacent tissues. [file Image_1.jpeg]

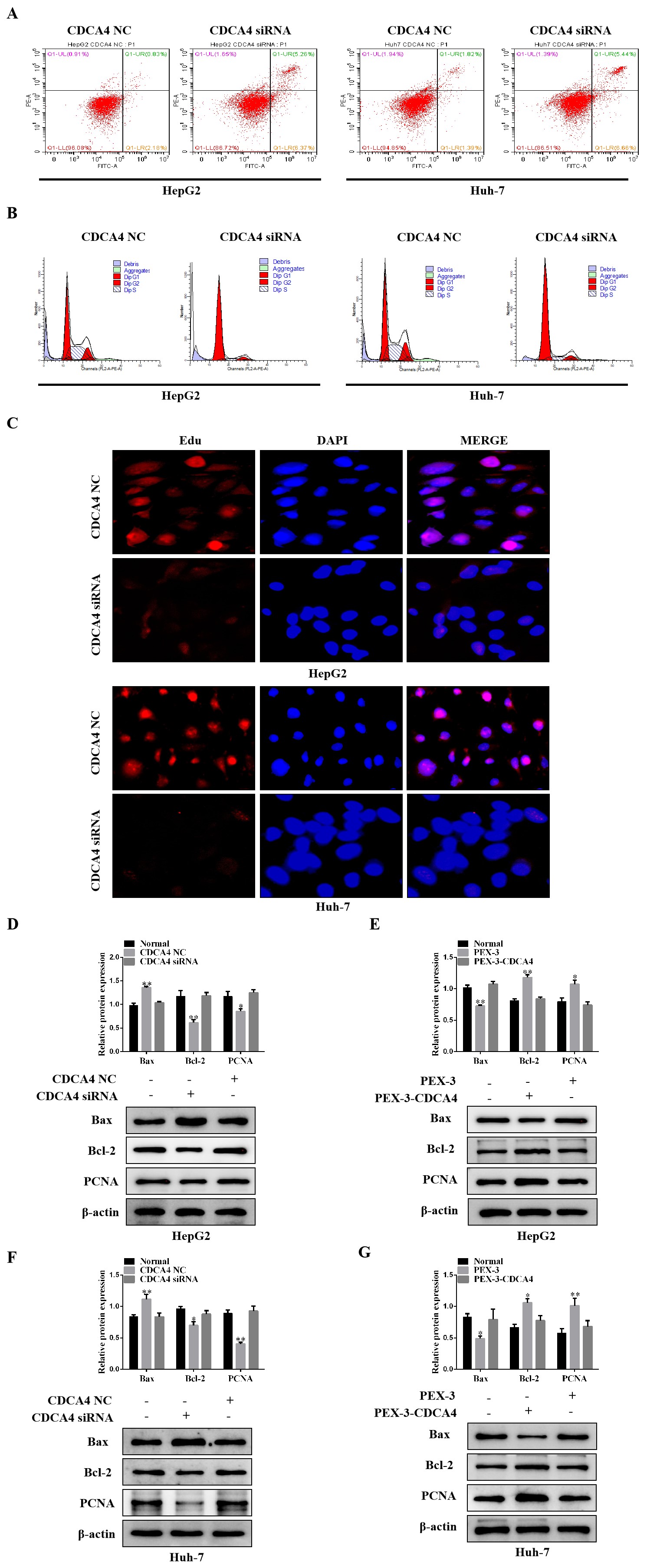

Supplement: Supplementary Figure 2 — (A) Flow cytometry results showed that CDCA4-siRNA significantly promoted HCC cell apoptosis; (B) Flow cytometry results showed that HCC cells transfected with CDCA4-siRNA displayed a larger G0/G1 population and arrested S and G2/M phase; (C) Edu staining showed that the CDCA4-siRNA significantly reduced HCC cell proliferation; (D) Western blotting results showed that PEX-3-CDCA4 inhibited the expression level of Bax, increased the expression level of Bcl-2 and PNCA in HepG2 cells; (E) Western blotting results showed that CDCA4-siRNA remarkably increased the expression level of Bax and inhibited the expression level of Bcl-2 and PNCA in HepG2 cells. (F) Western blotting results showed that PEX-3-CDCA4 inhibited the expression level of Bax, increased the expression level of Bcl-2 and PNCA in Huh-7 cells; (G) Western blotting results showed that CDCA4-siRNA remarkably increased the expression level of Bax and inhibited the expression level of Bcl-2 and PNCA in Huh-7 cells. [file Image_2.jpeg]

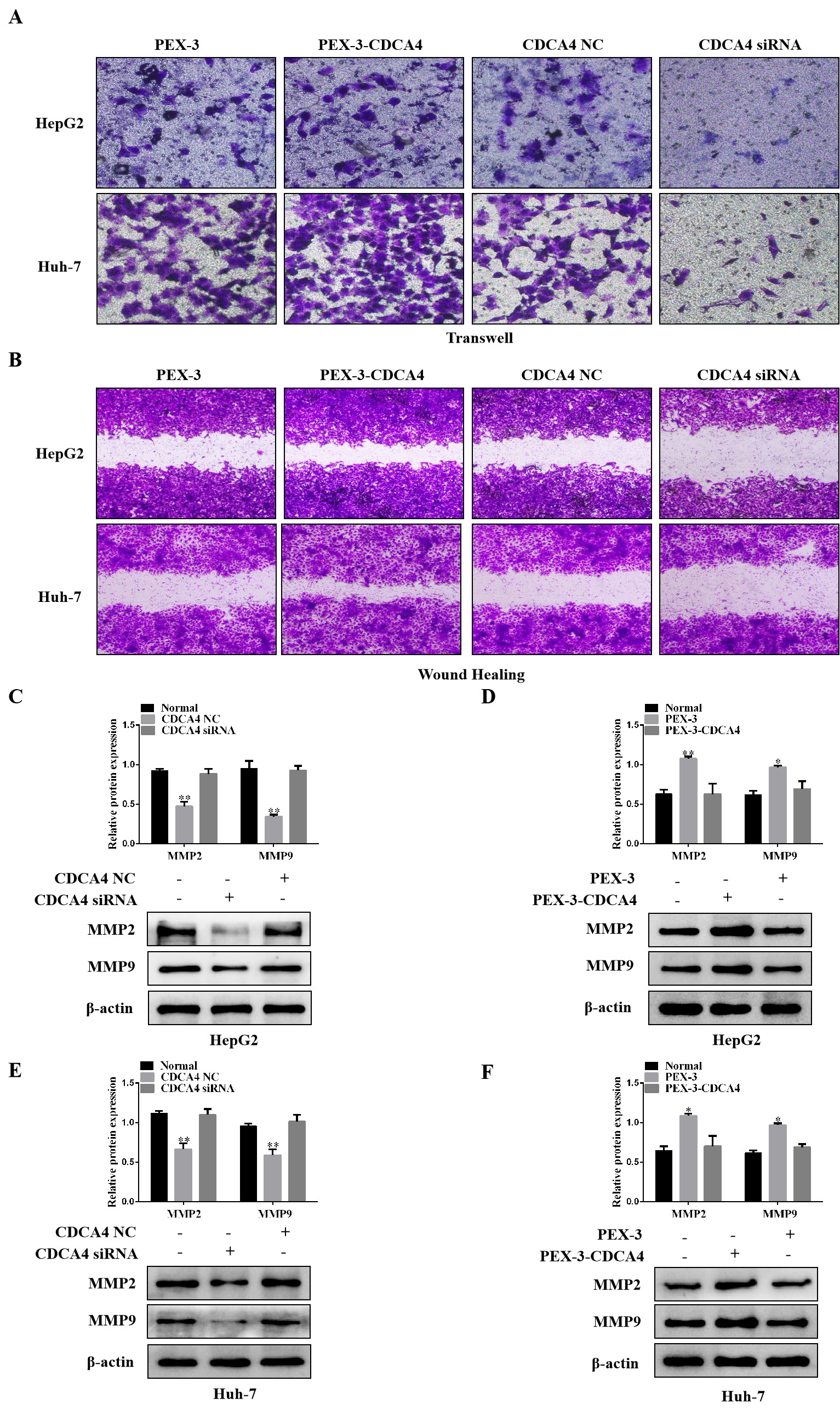

Supplement: Supplementary Figure 3 — (A)The results of Transwell analysis indicated that the ability of invasion in HCC cells was enhanced after transfection with PEX-3-CDCA4; (B) The results of Wound Healing analysis indicated that the ability of migration in HCC cells was enhanced after transfection with PEX-3-CDCA4. (C) Western blotting results showed that CDCA4-siRNA inhibited the expression level of Bax, increased the expression level of MMP2 and MMP9 in HepG2 cells; (D) Western blotting results showed that PEX-3-CDCA4 remarkably increased the expression level of Bax and inhibited the expression level of MMP2 and MMP9 in HepG2 cells. (E) Western blotting results showed that CDCA4-siRNA inhibited the expression level of Bax, increased the expression level of MMP2 and MMP9 in Huh7 cells; (F) Western blotting results showed that PEX-3-CDCA4 remarkably increased the expression level of Bax and inhibited the expression level of MMP2 and MMP9 in Huh7 cells. [file Image_3.jpeg]

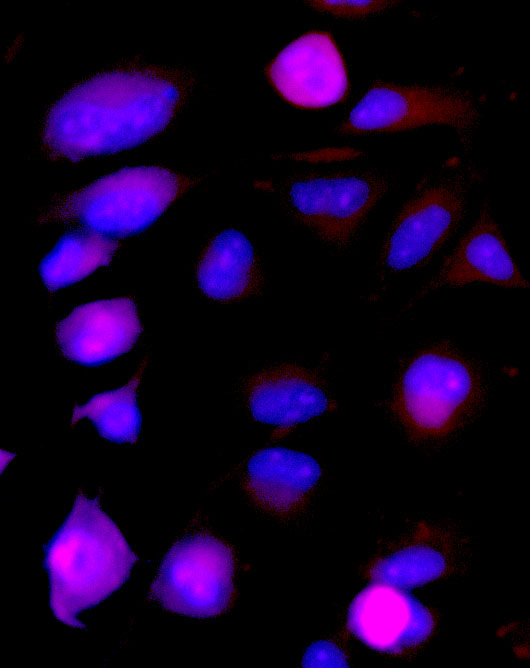

Supplement: Supplementary file 5 [file DataSheet_2.zip › Original Data/Edu/1.jpg]

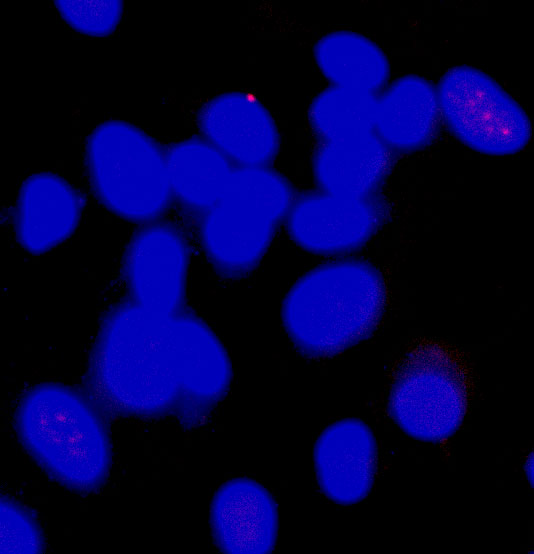

Supplement: Supplementary file 5 [file DataSheet_2.zip › Original Data/Edu/10.jpg]

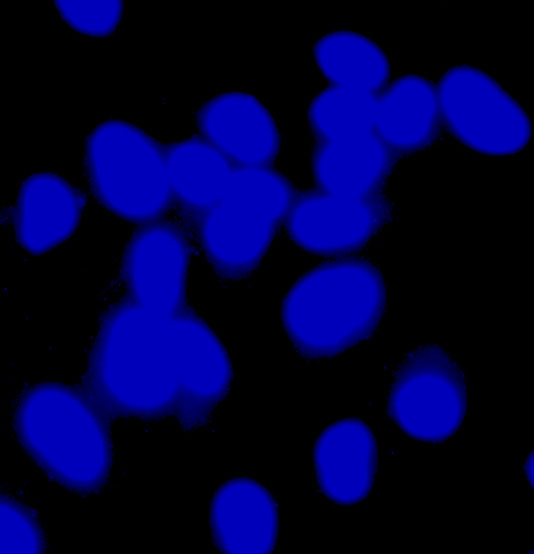

Supplement: Supplementary file 5 [file DataSheet_2.zip › Original Data/Edu/11.jpg]

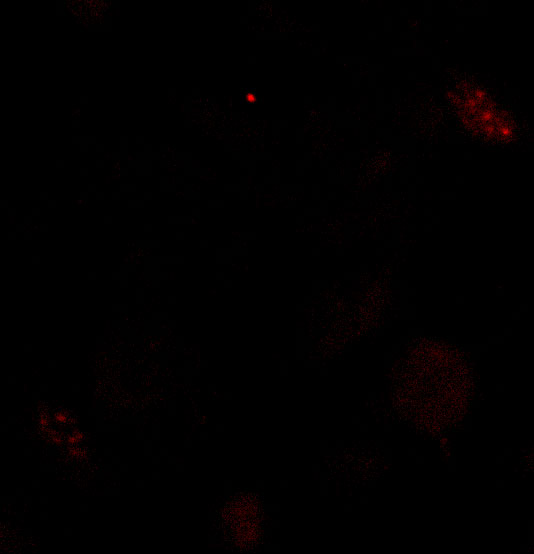

Supplement: Supplementary file 5 [file DataSheet_2.zip › Original Data/Edu/12.jpg]

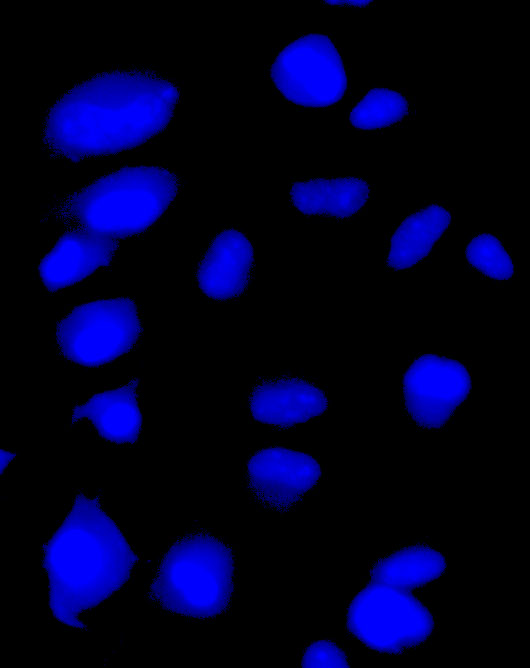

Supplement: Supplementary file 5 [file DataSheet_2.zip › Original Data/Edu/2.jpg]

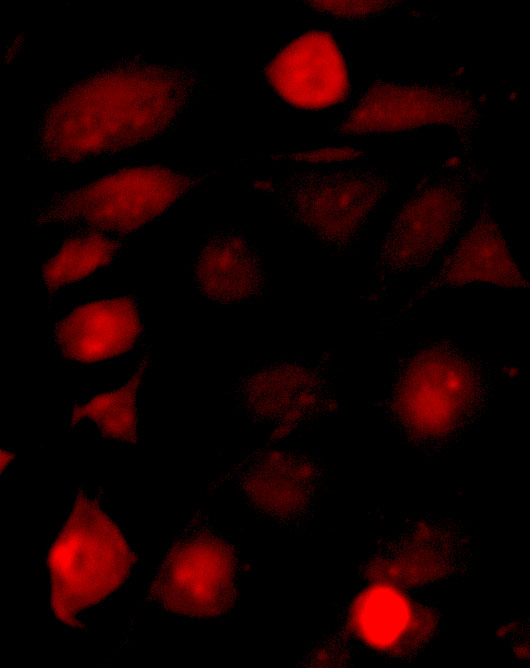

Supplement: Supplementary file 5 [file DataSheet_2.zip › Original Data/Edu/3.jpg]

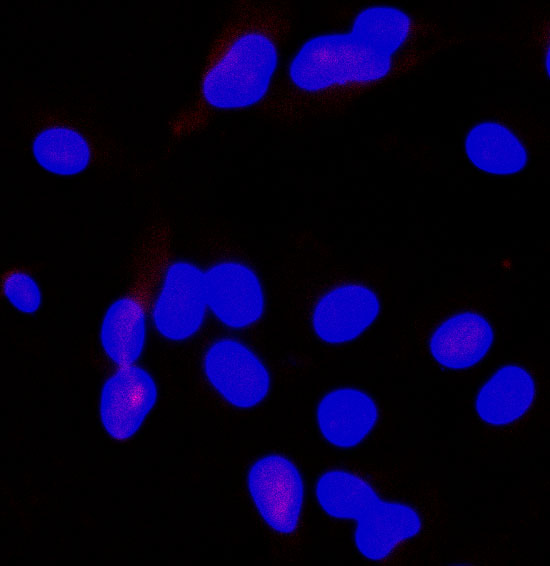

Supplement: Supplementary file 5 [file DataSheet_2.zip › Original Data/Edu/4.jpg]

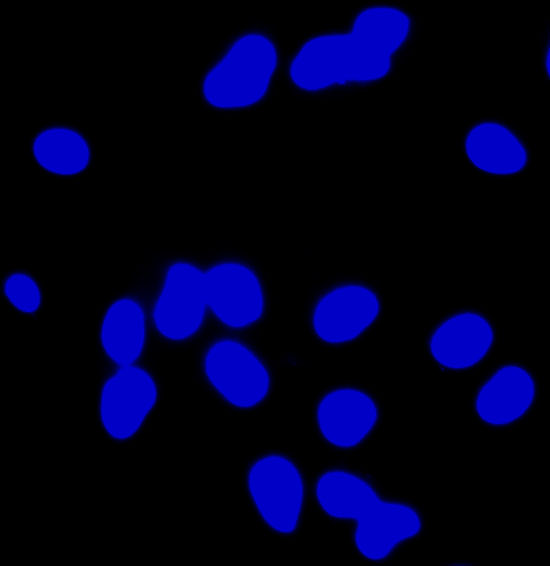

Supplement: Supplementary file 5 [file DataSheet_2.zip › Original Data/Edu/5.jpg]

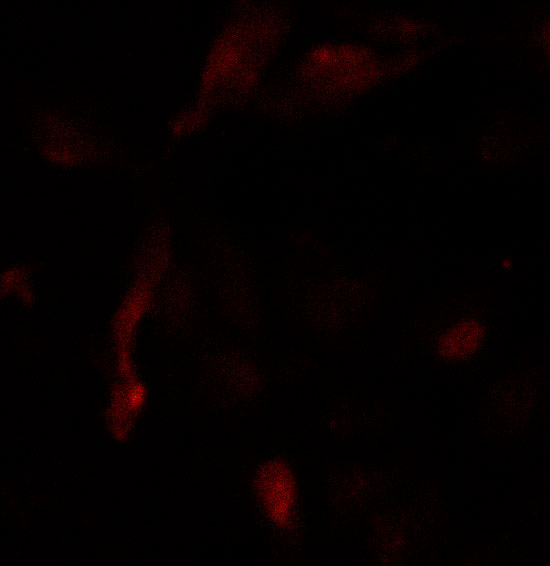

Supplement: Supplementary file 5 [file DataSheet_2.zip › Original Data/Edu/6.jpg]

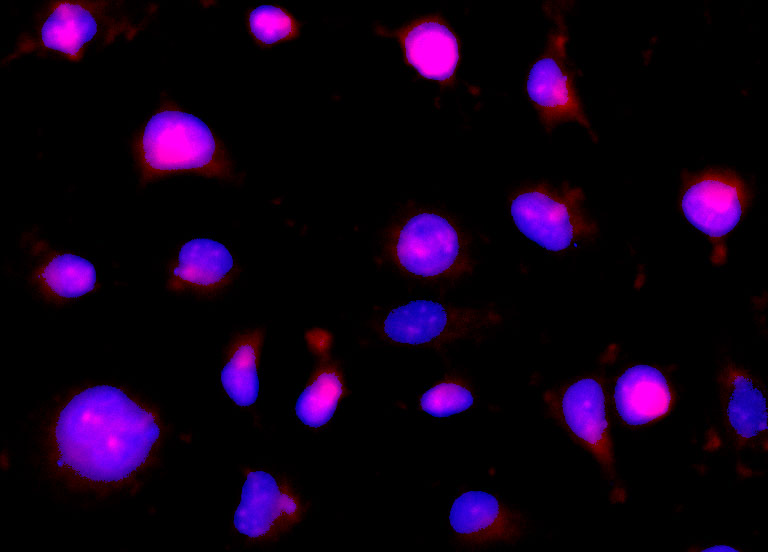

Supplement: Supplementary file 5 [file DataSheet_2.zip › Original Data/Edu/7.jpg]

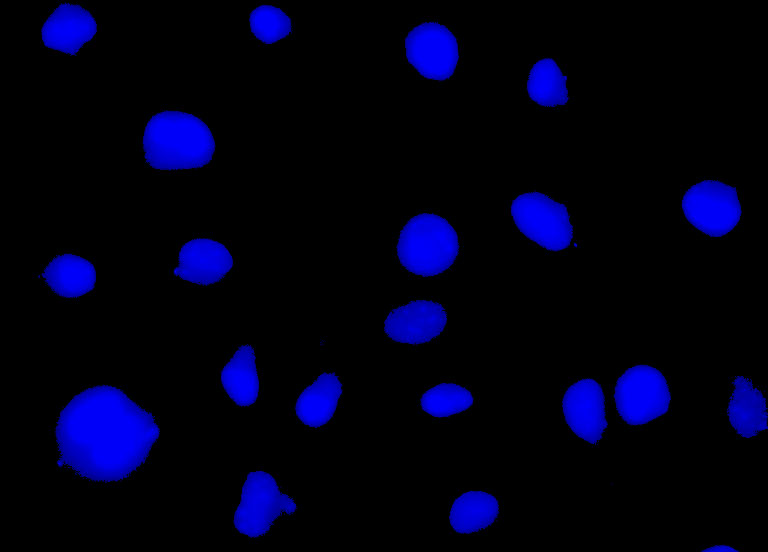

Supplement: Supplementary file 5 [file DataSheet_2.zip › Original Data/Edu/8.jpg]

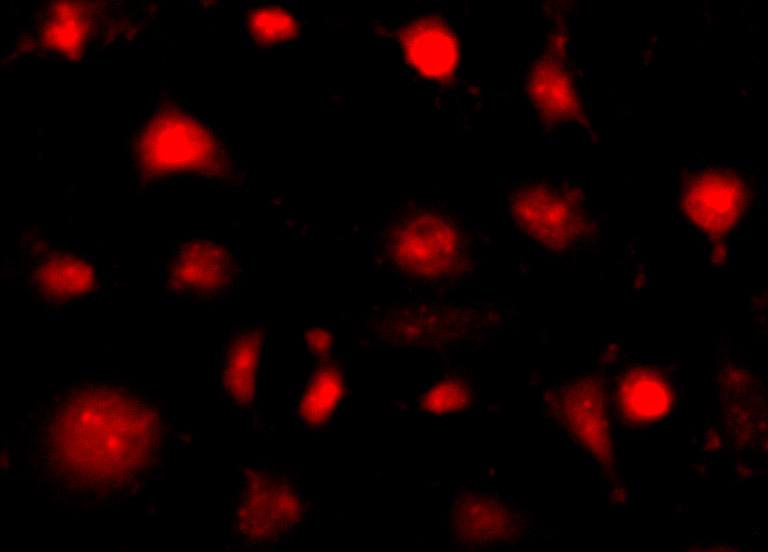

Supplement: Supplementary file 5 [file DataSheet_2.zip › Original Data/Edu/9.jpg]

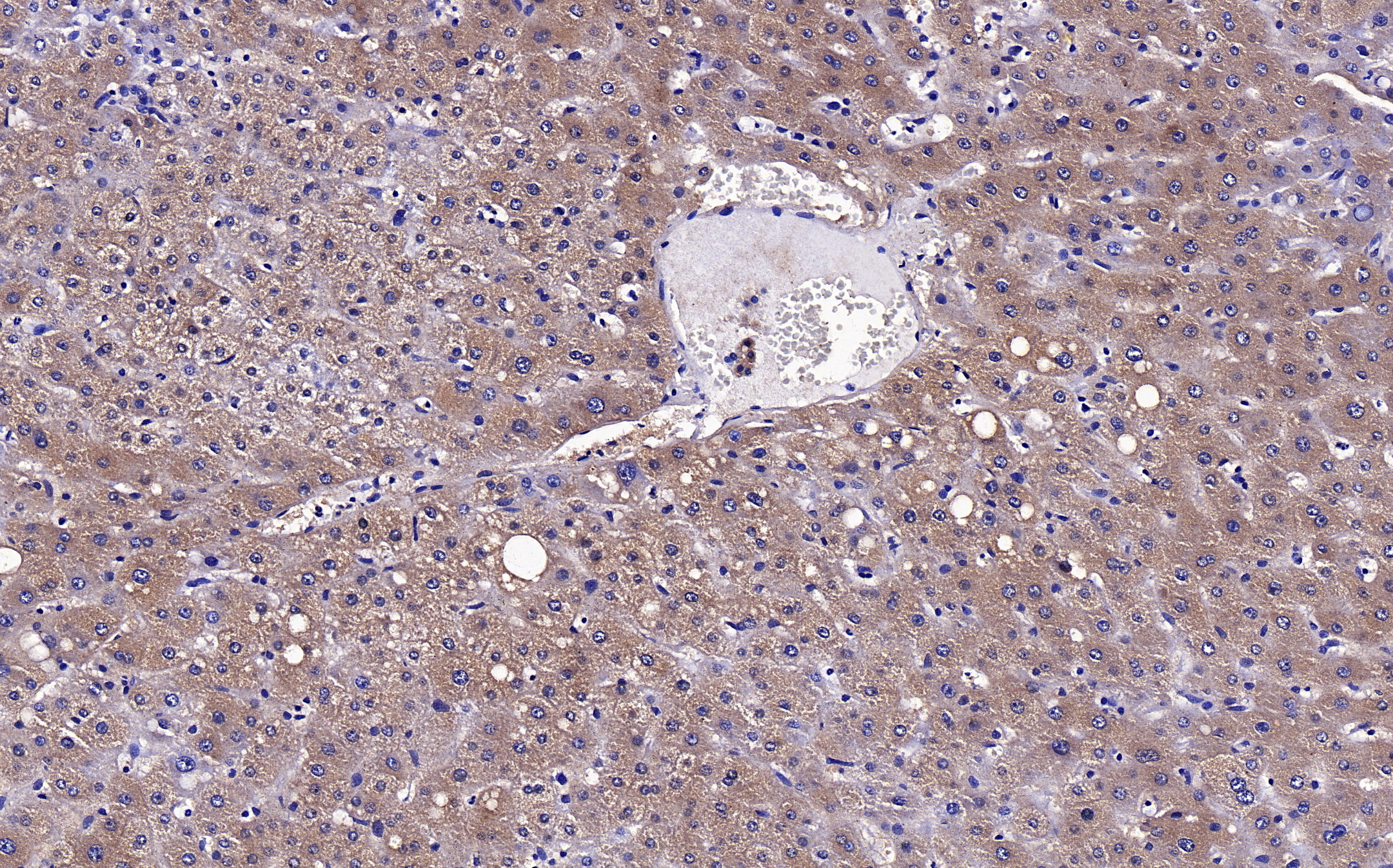

Supplement: Supplementary file 5 [file DataSheet_2.zip › Original Data/IHC(CDCA4)/1.jpg]

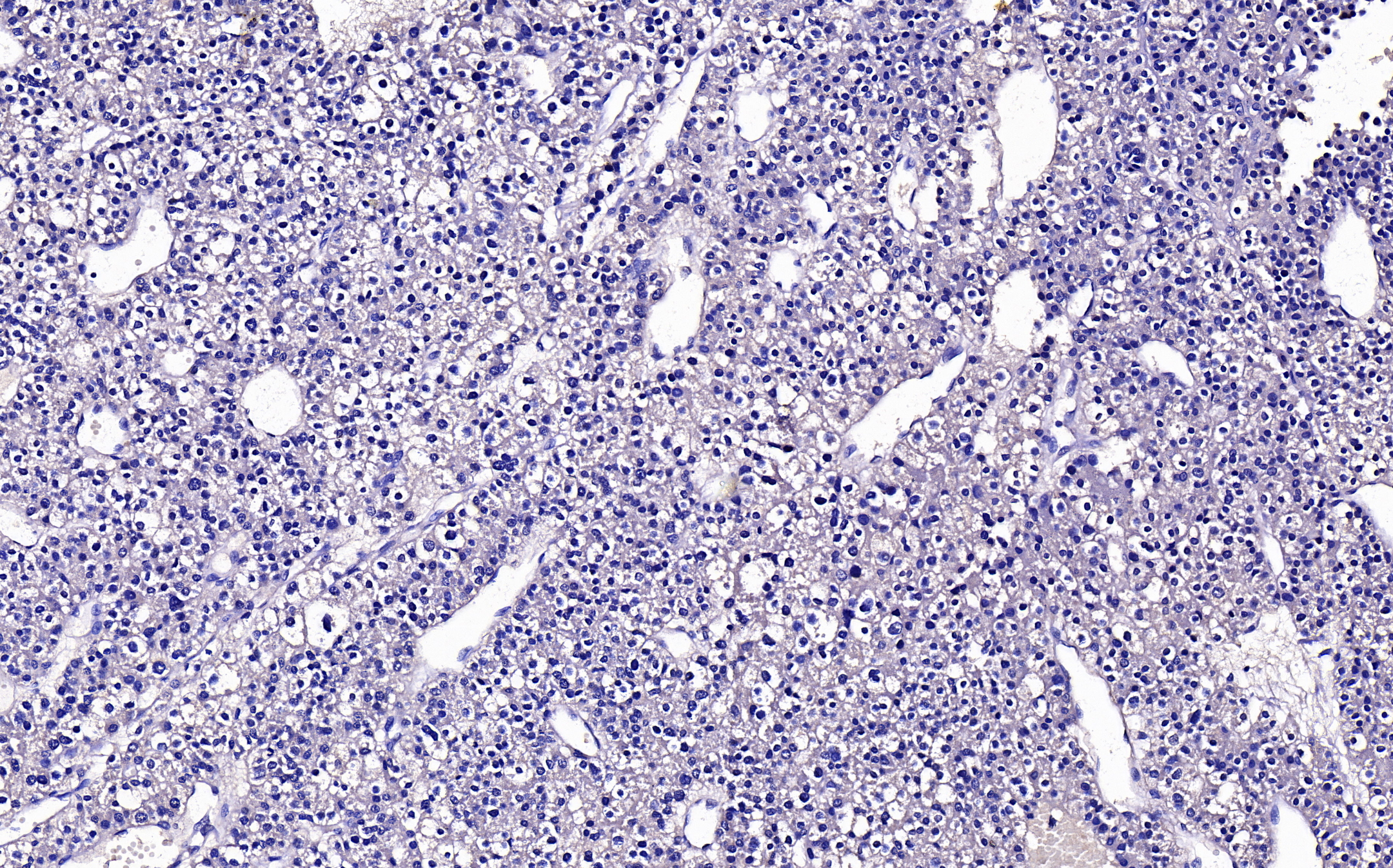

Supplement: Supplementary file 5 [file DataSheet_2.zip › Original Data/IHC(CDCA4)/2.jpg]

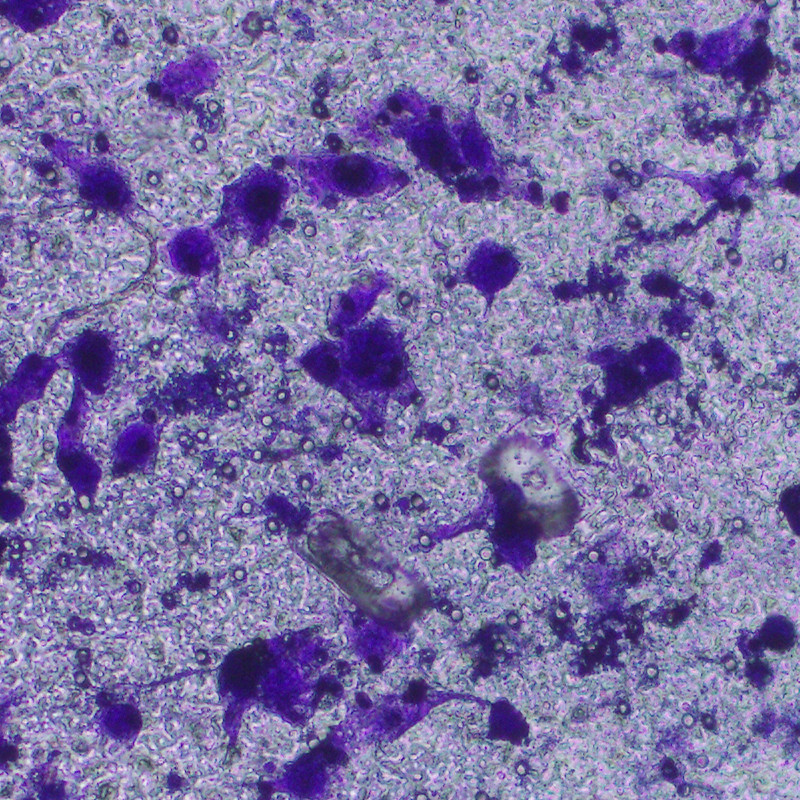

Supplement: Supplementary file 5 [file DataSheet_2.zip › Original Data/Transwell/1.jpg]

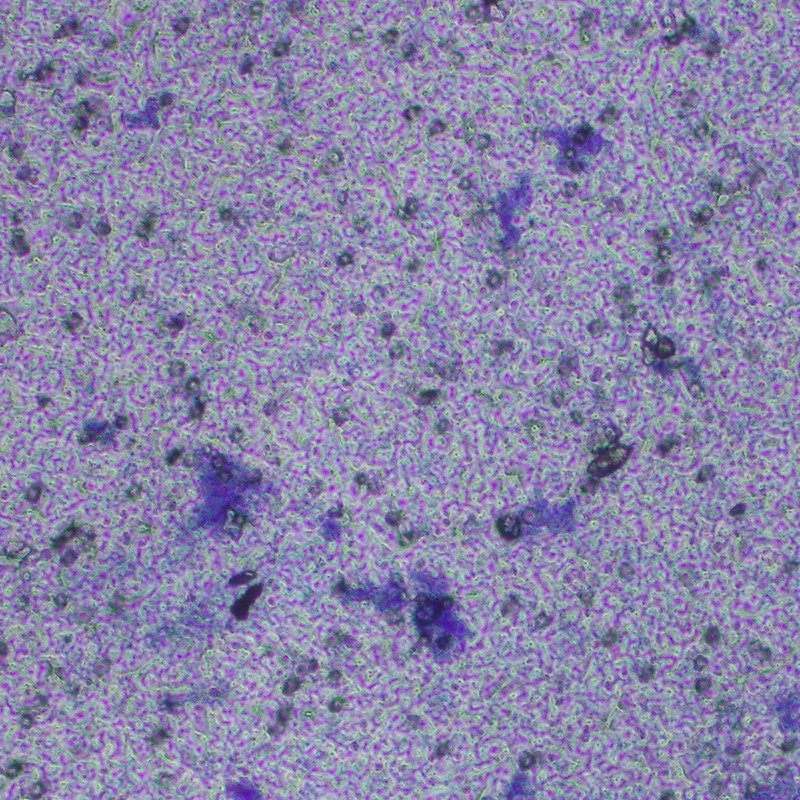

Supplement: Supplementary file 5 [file DataSheet_2.zip › Original Data/Transwell/2.jpg]

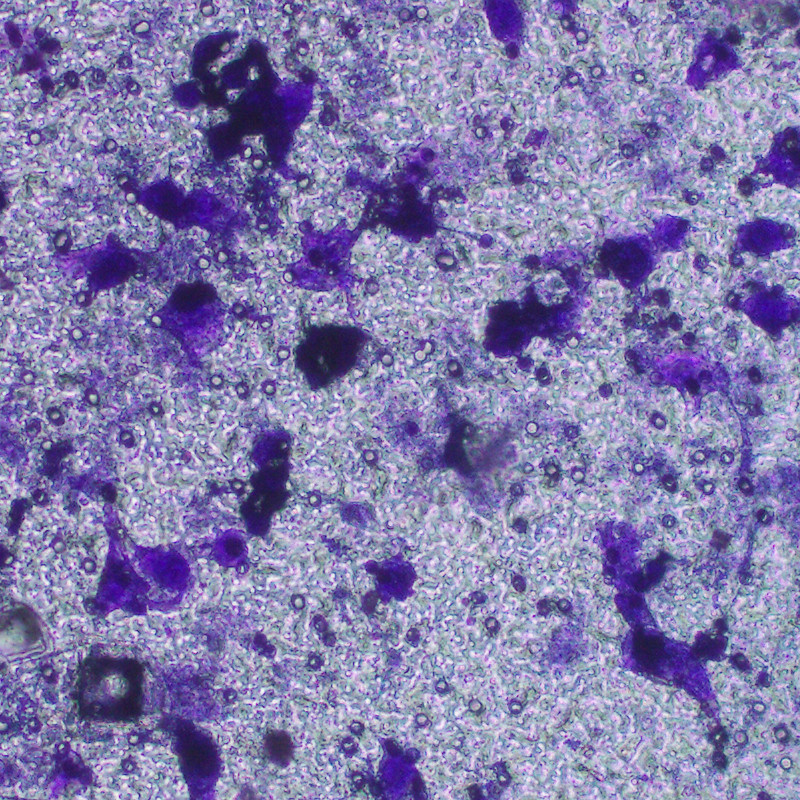

Supplement: Supplementary file 5 [file DataSheet_2.zip › Original Data/Transwell/3.jpg]

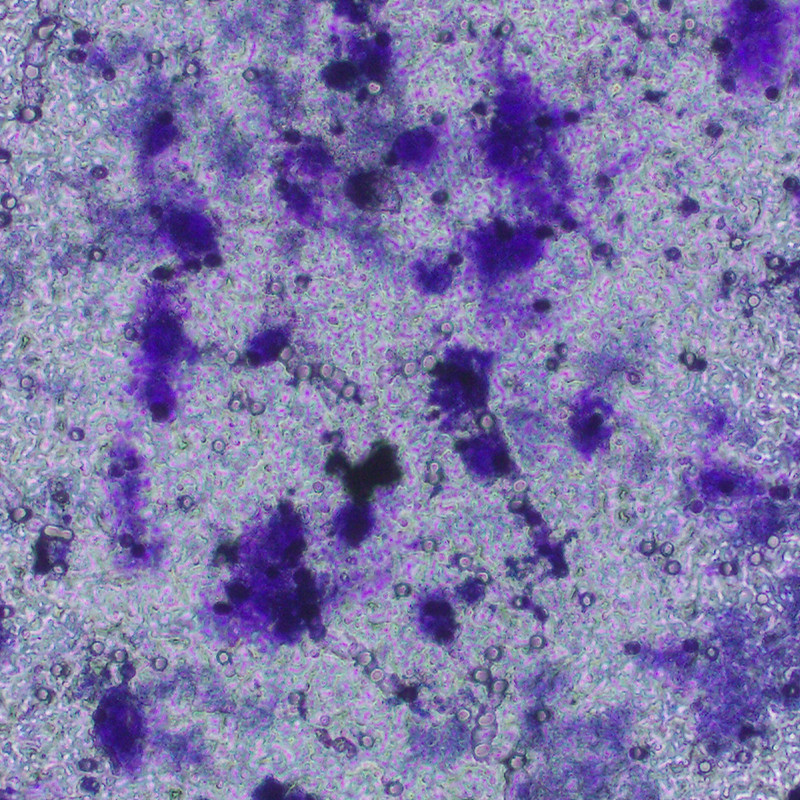

Supplement: Supplementary file 5 [file DataSheet_2.zip › Original Data/Transwell/4.jpg]

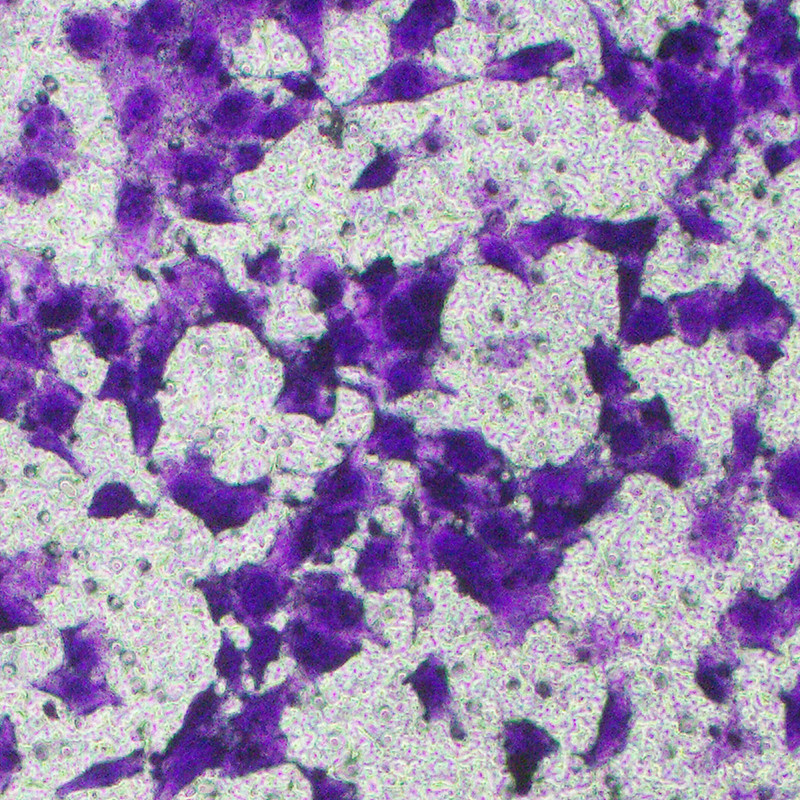

Supplement: Supplementary file 5 [file DataSheet_2.zip › Original Data/Transwell/5.jpg]

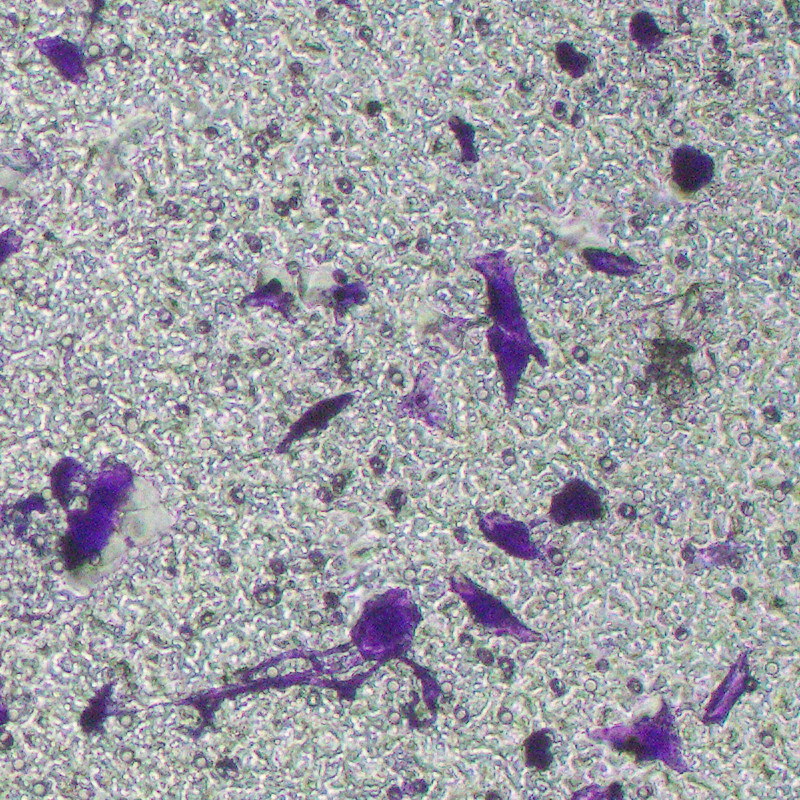

Supplement: Supplementary file 5 [file DataSheet_2.zip › Original Data/Transwell/6.jpg]

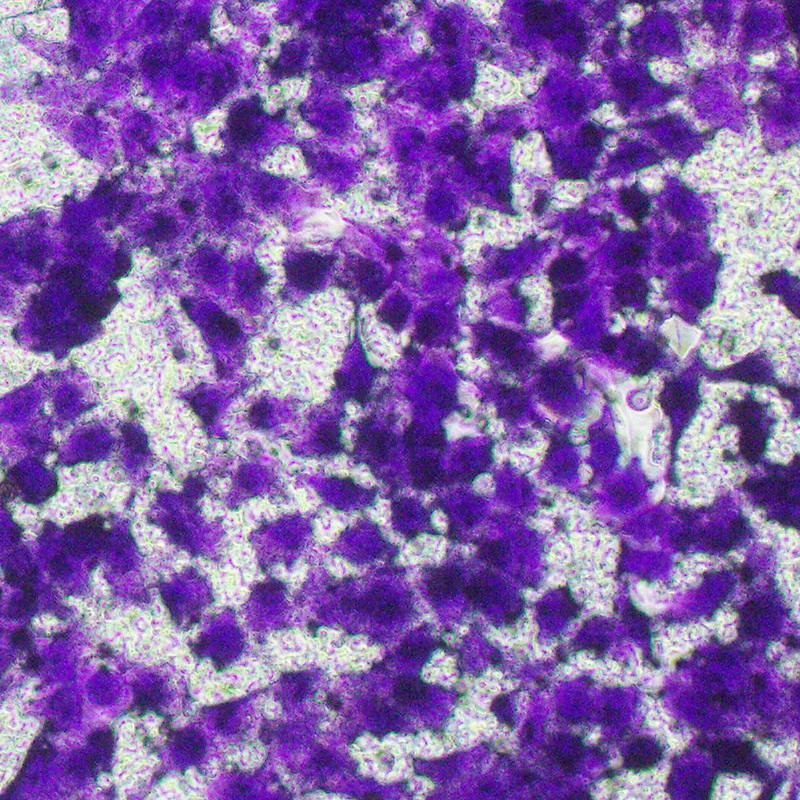

Supplement: Supplementary file 5 [file DataSheet_2.zip › Original Data/Transwell/7.jpg]

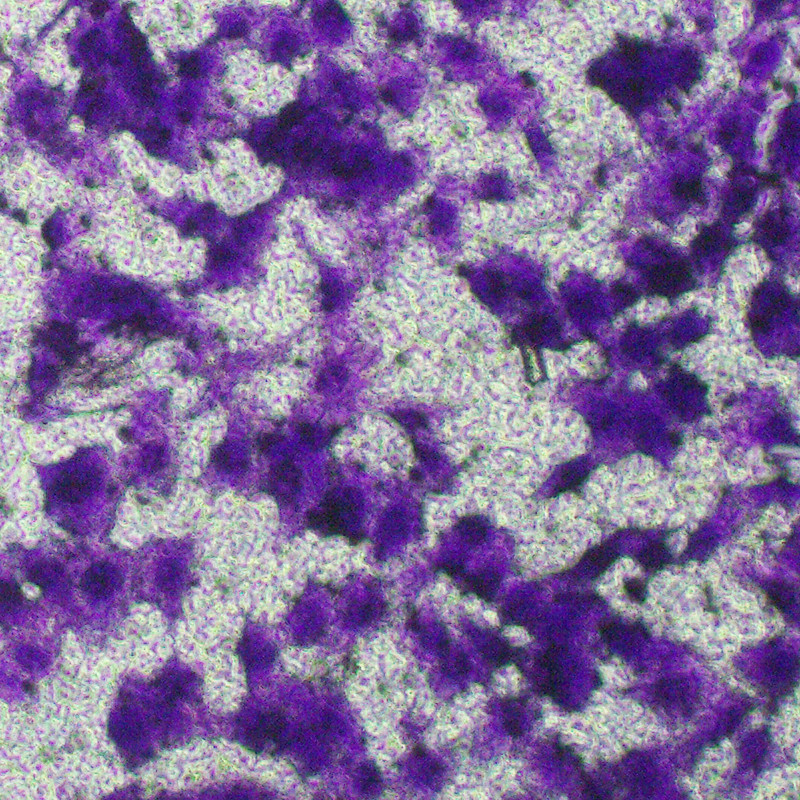

Supplement: Supplementary file 5 [file DataSheet_2.zip › Original Data/Transwell/8.jpg]

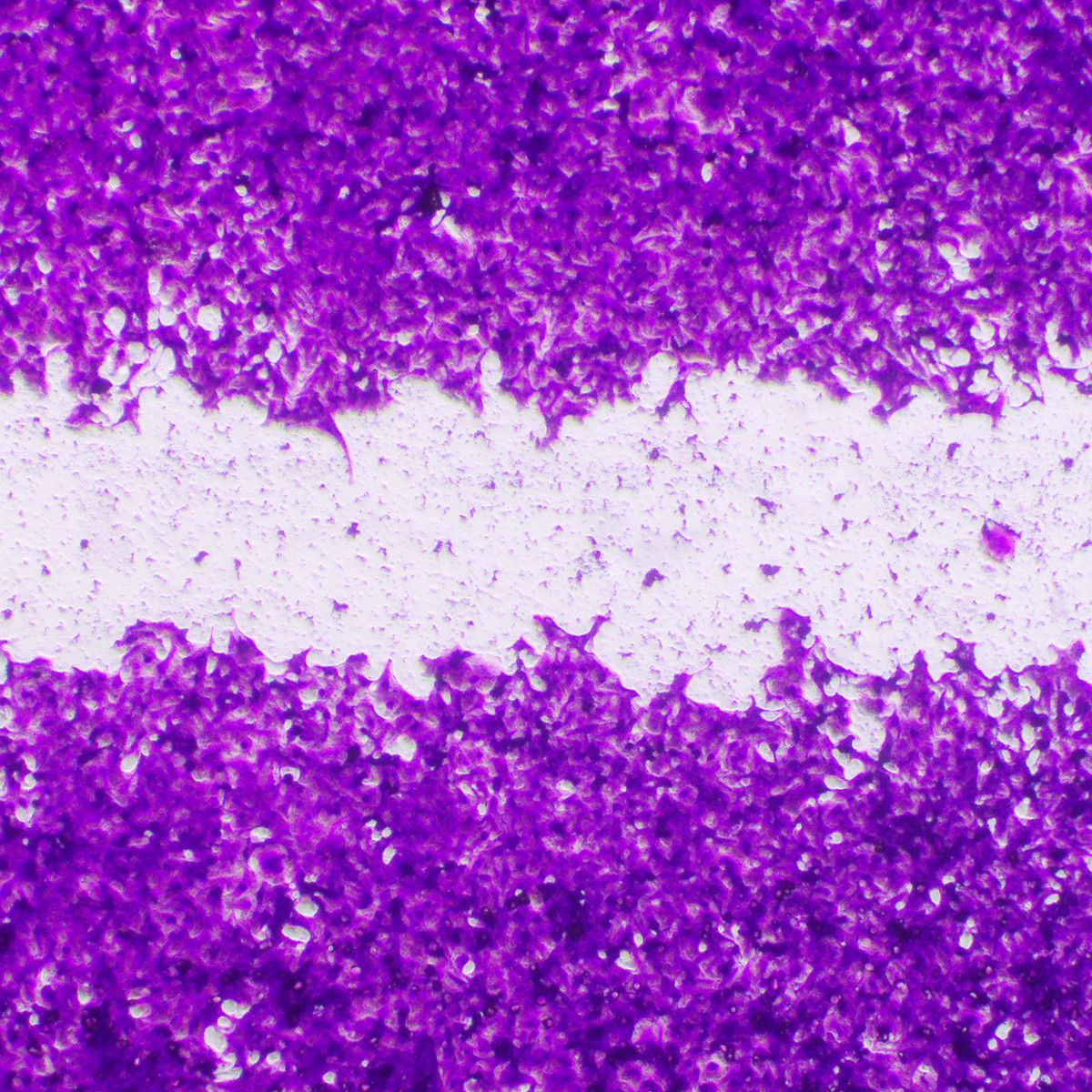

Supplement: Supplementary file 5 [file DataSheet_2.zip › Original Data/Wound Healing/1.jpg]

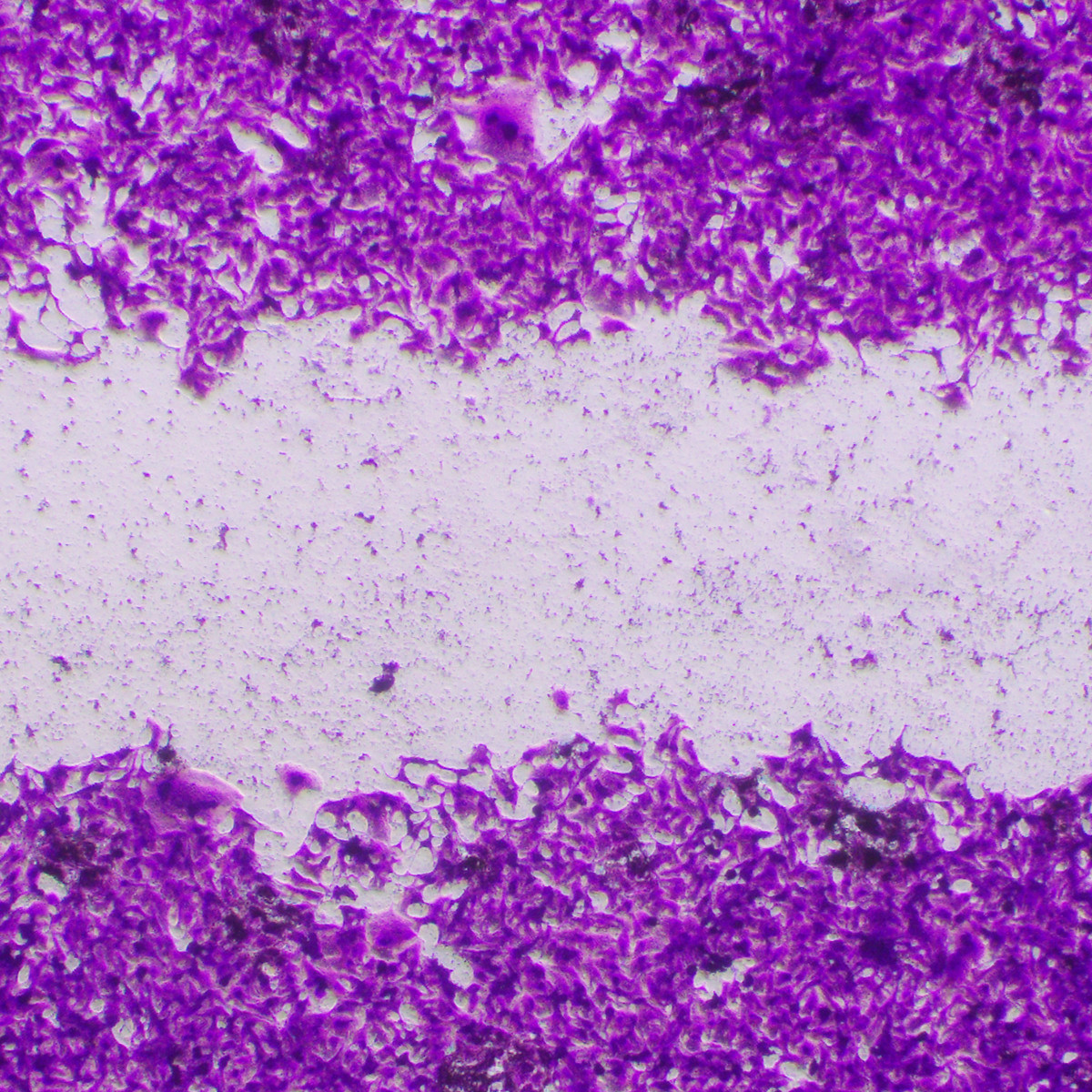

Supplement: Supplementary file 5 [file DataSheet_2.zip › Original Data/Wound Healing/2.jpg]

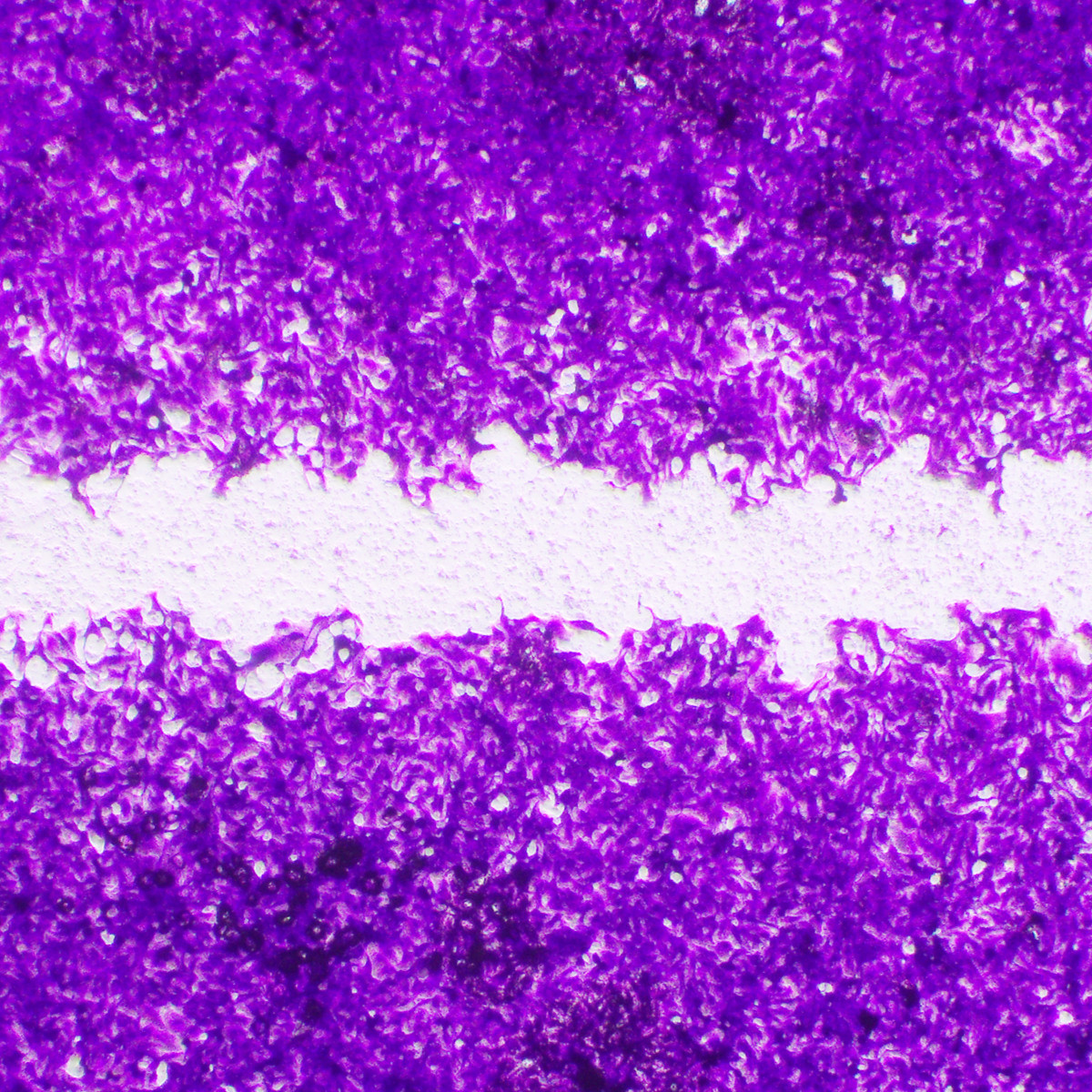

Supplement: Supplementary file 5 [file DataSheet_2.zip › Original Data/Wound Healing/3.jpg]

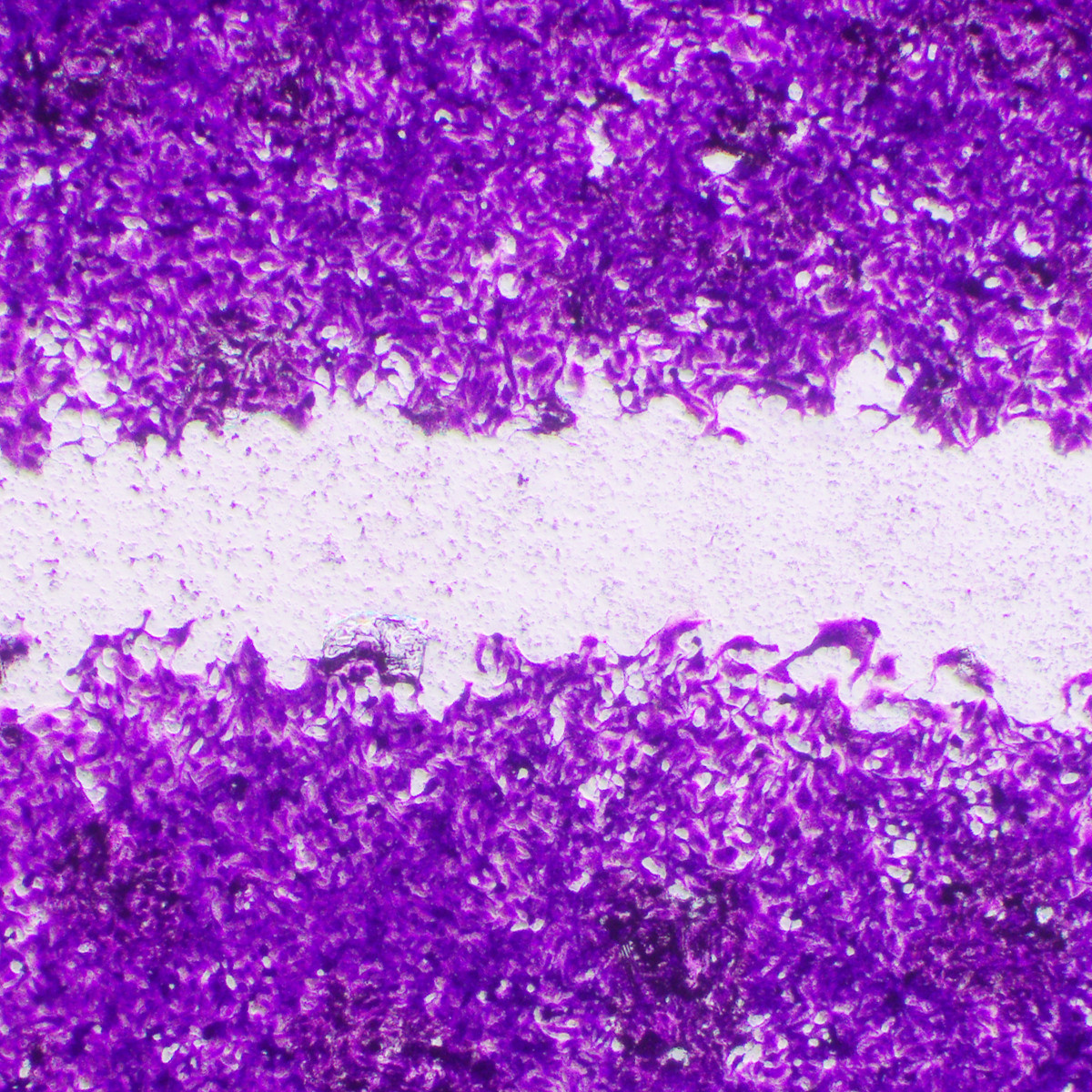

Supplement: Supplementary file 5 [file DataSheet_2.zip › Original Data/Wound Healing/4.jpg]

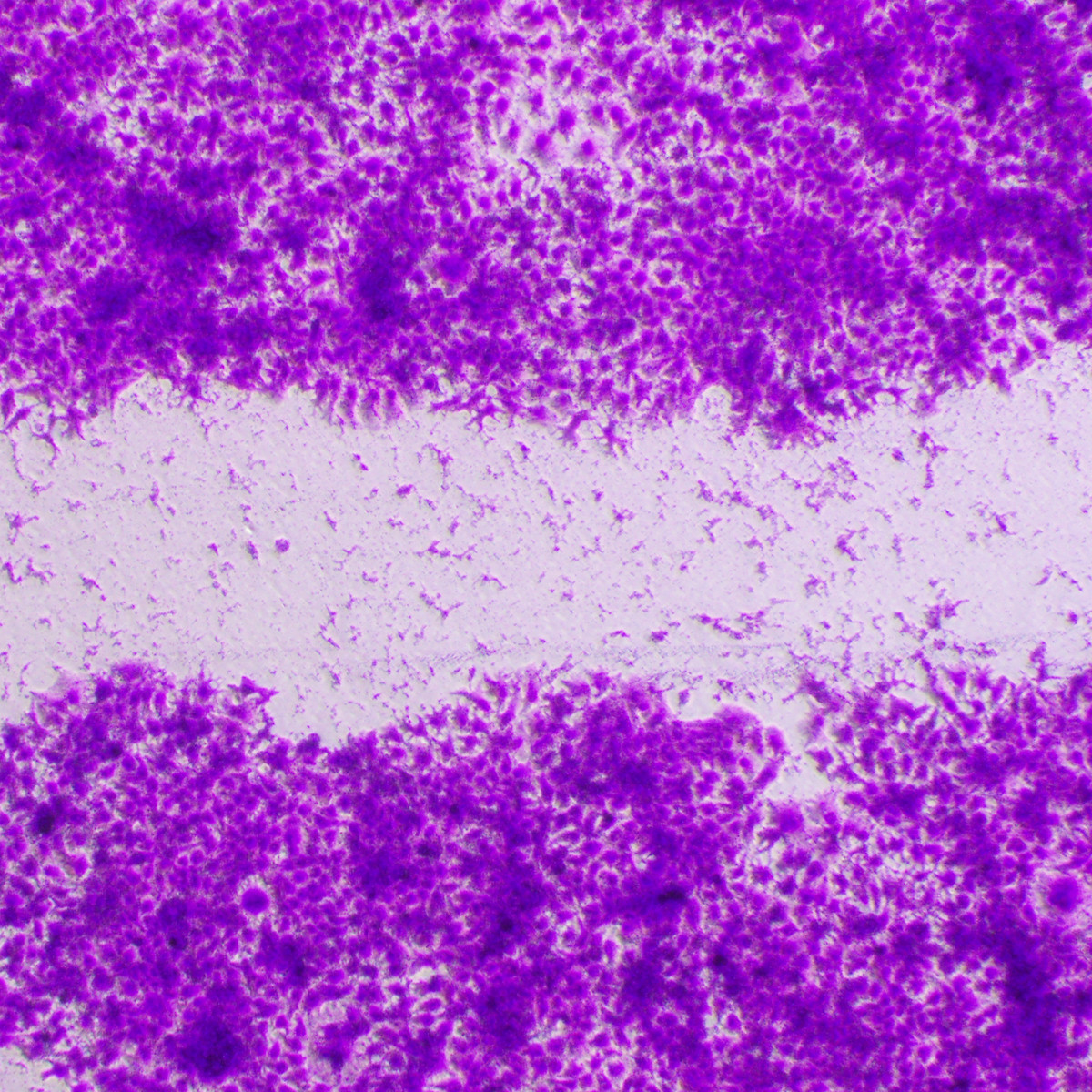

Supplement: Supplementary file 5 [file DataSheet_2.zip › Original Data/Wound Healing/5.jpg]

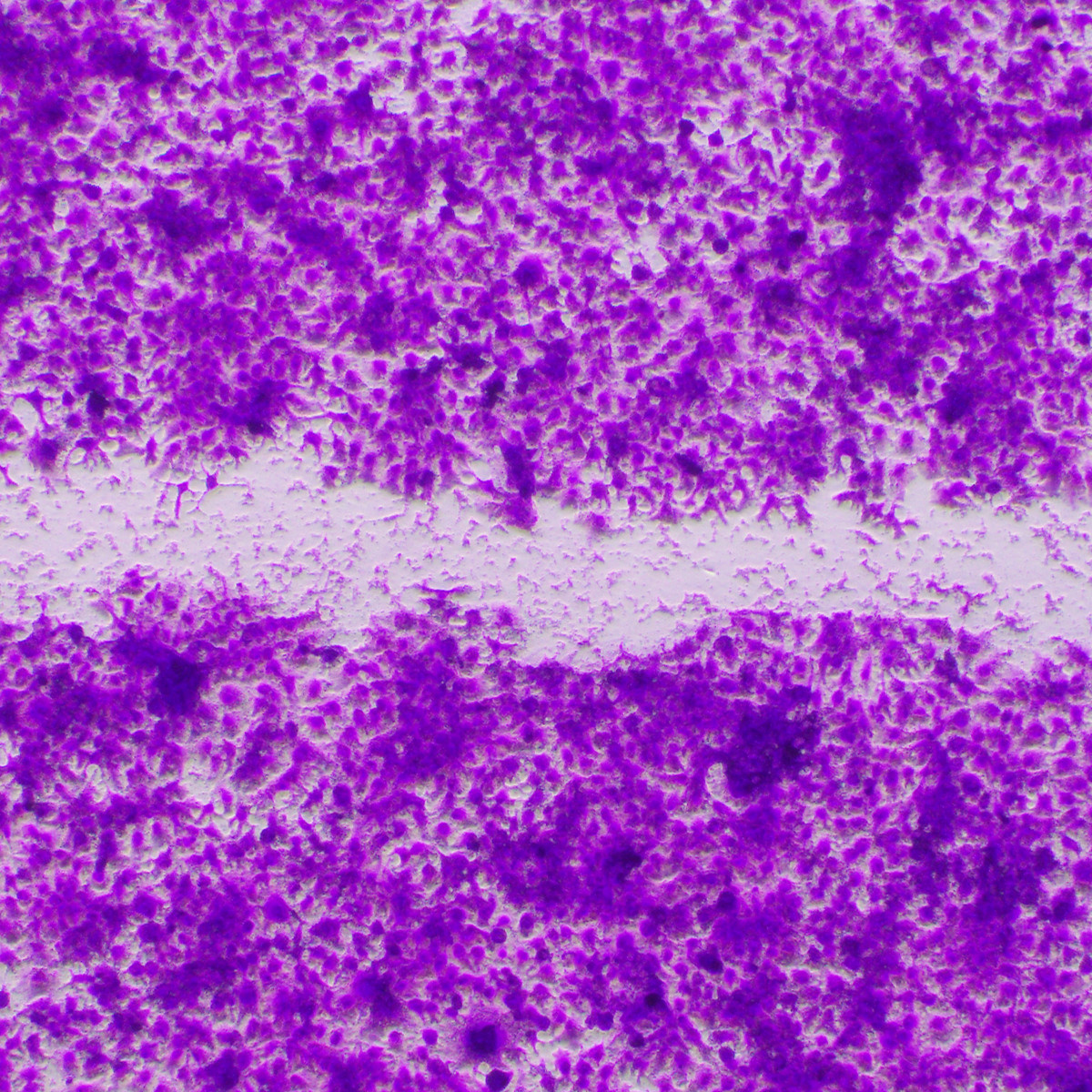

Supplement: Supplementary file 5 [file DataSheet_2.zip › Original Data/Wound Healing/6.jpg]

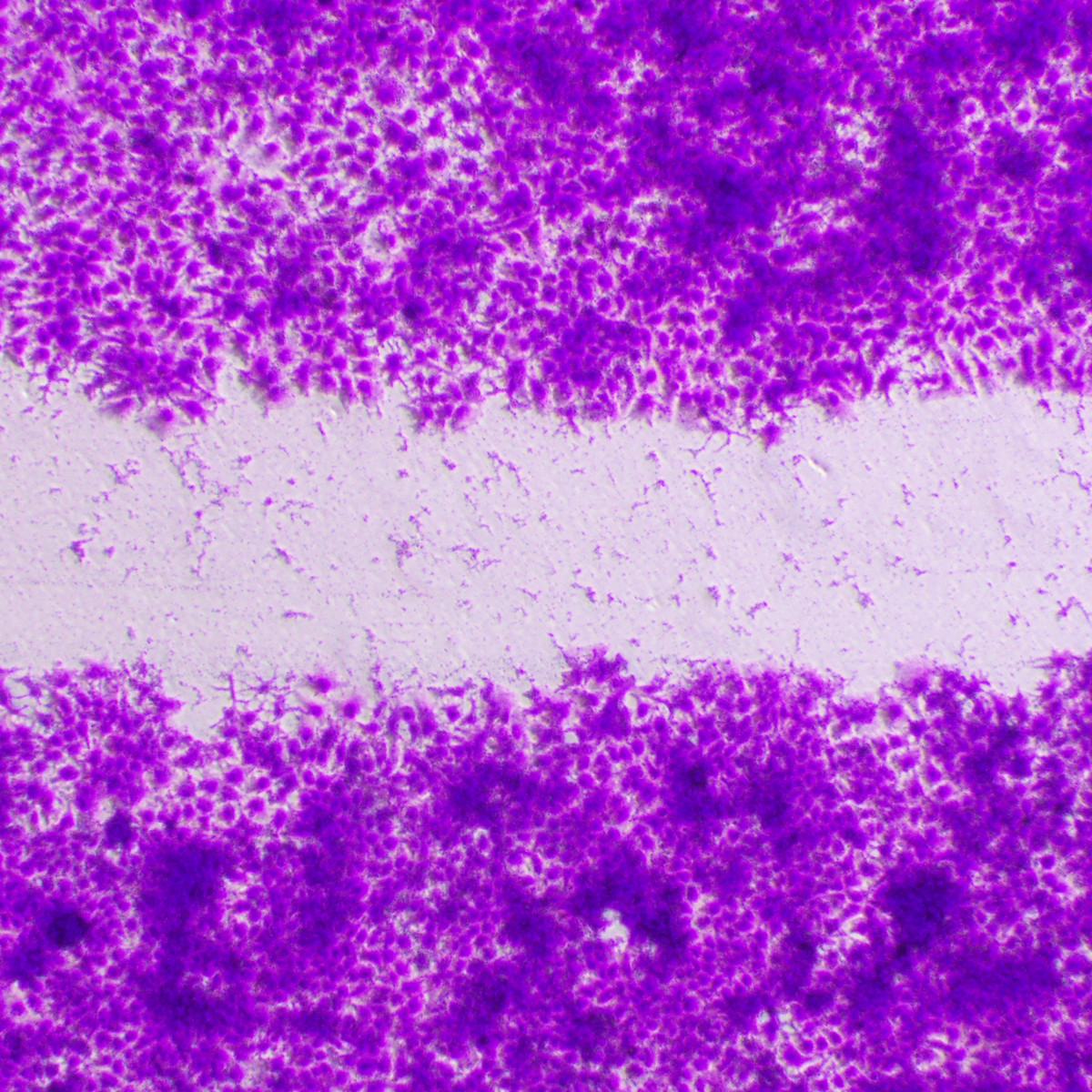

Supplement: Supplementary file 5 [file DataSheet_2.zip › Original Data/Wound Healing/7.jpg]

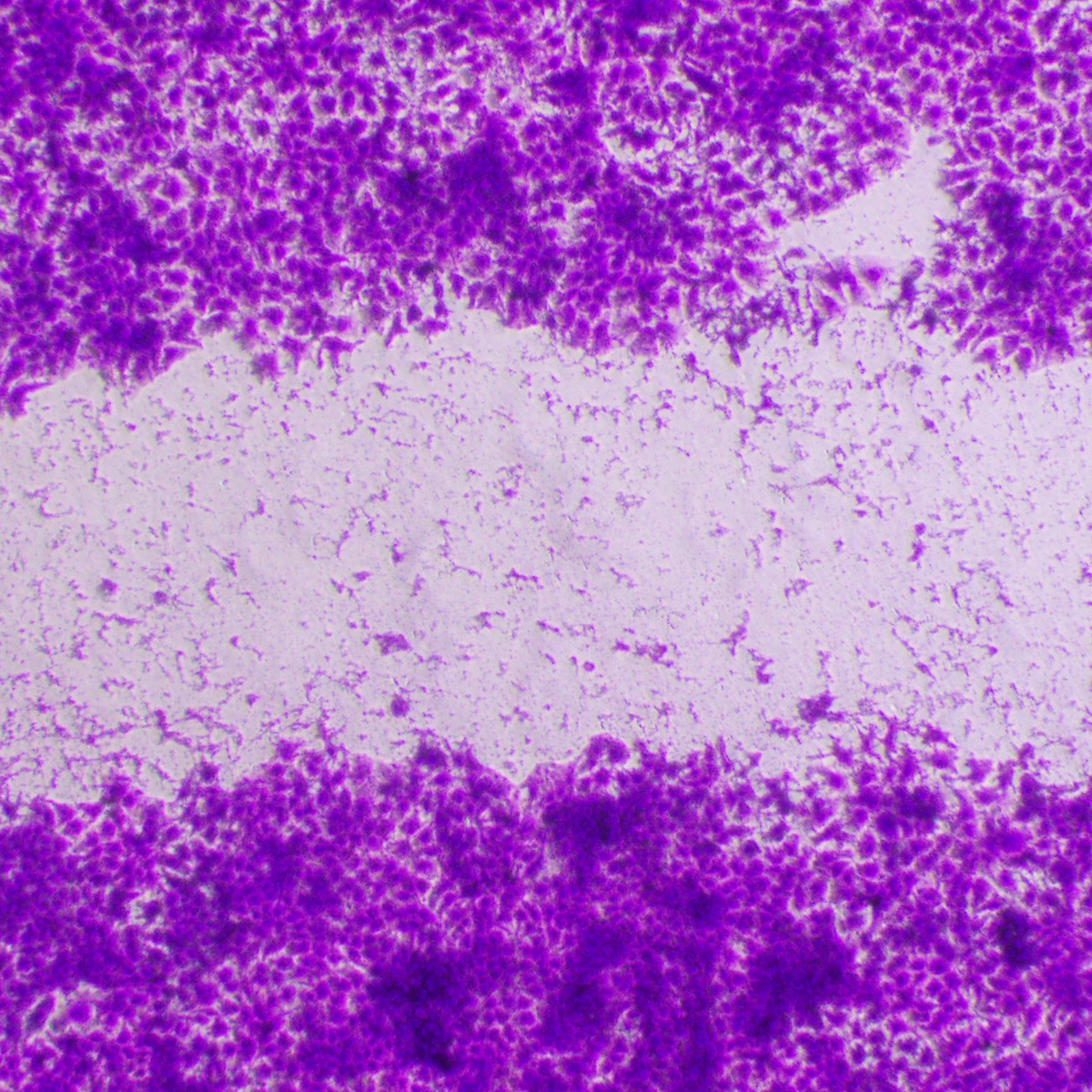

Supplement: Supplementary file 5 [file DataSheet_2.zip › Original Data/Wound Healing/8.jpg]

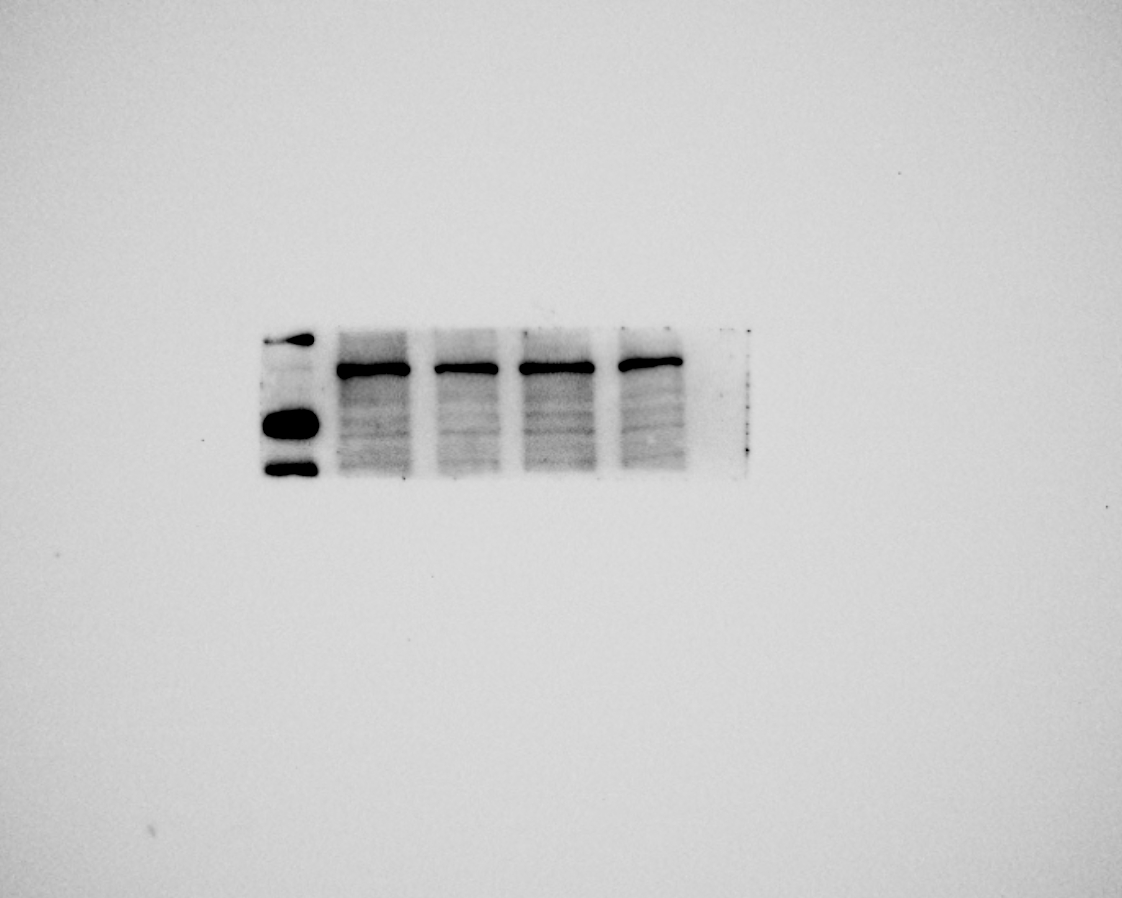

Supplement: Supplementary file 6 [file DataSheet_3.zip › Original Image of Western Blot/1.jpg]

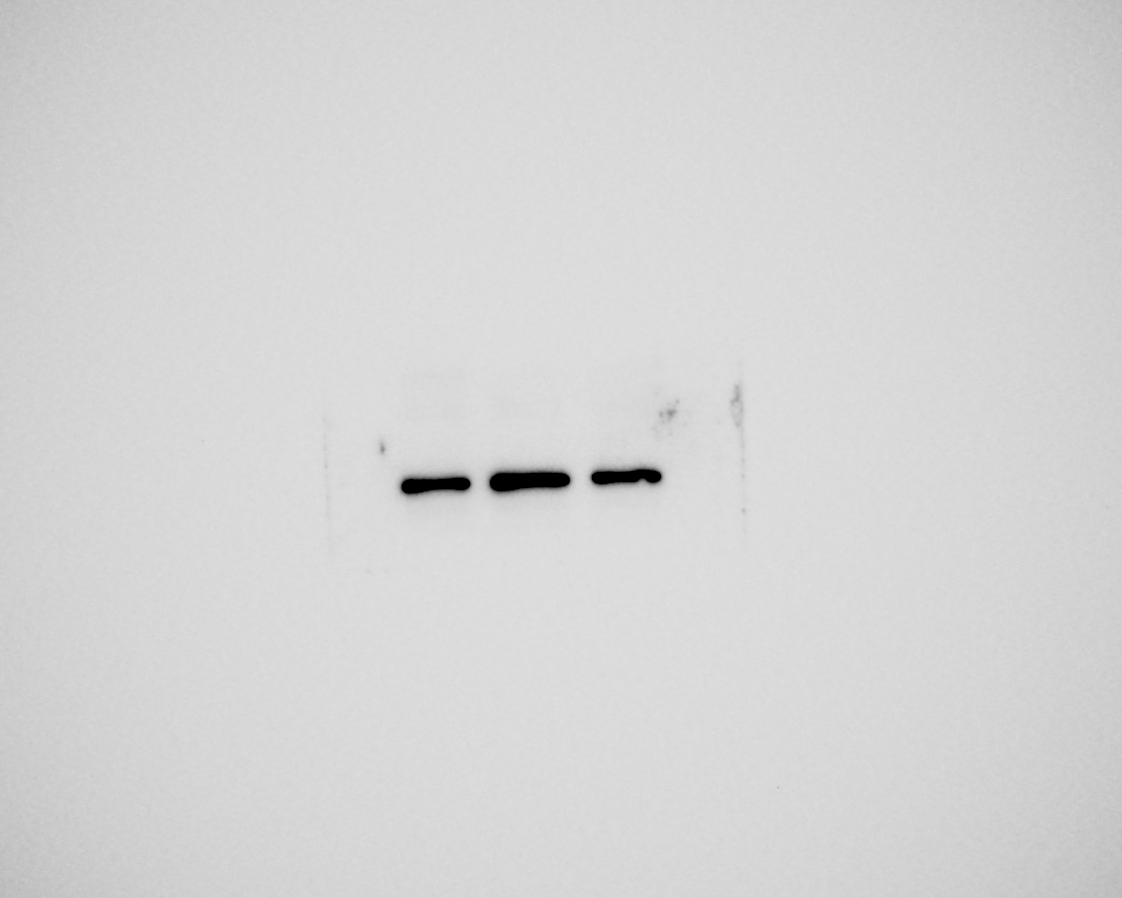

Supplement: Supplementary file 6 [file DataSheet_3.zip › Original Image of Western Blot/10.jpg]

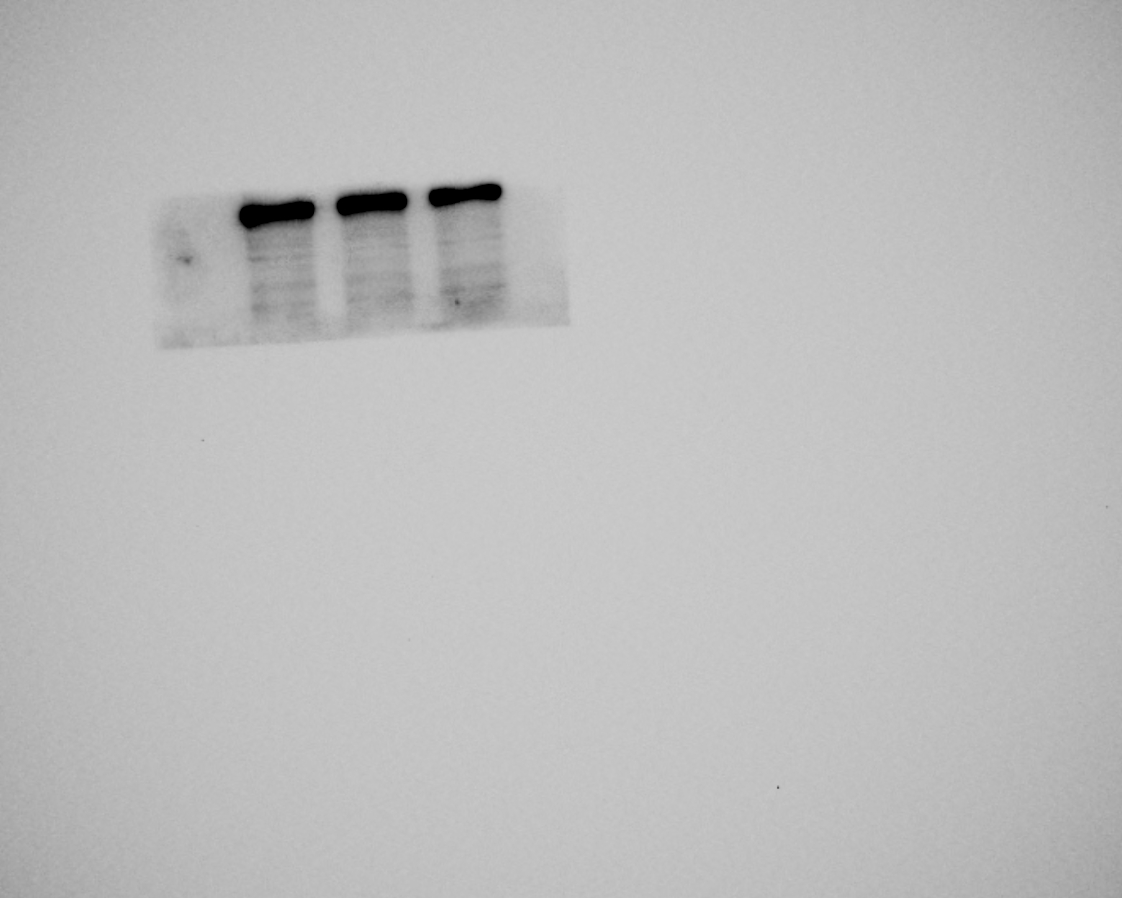

Supplement: Supplementary file 6 [file DataSheet_3.zip › Original Image of Western Blot/11.jpg]

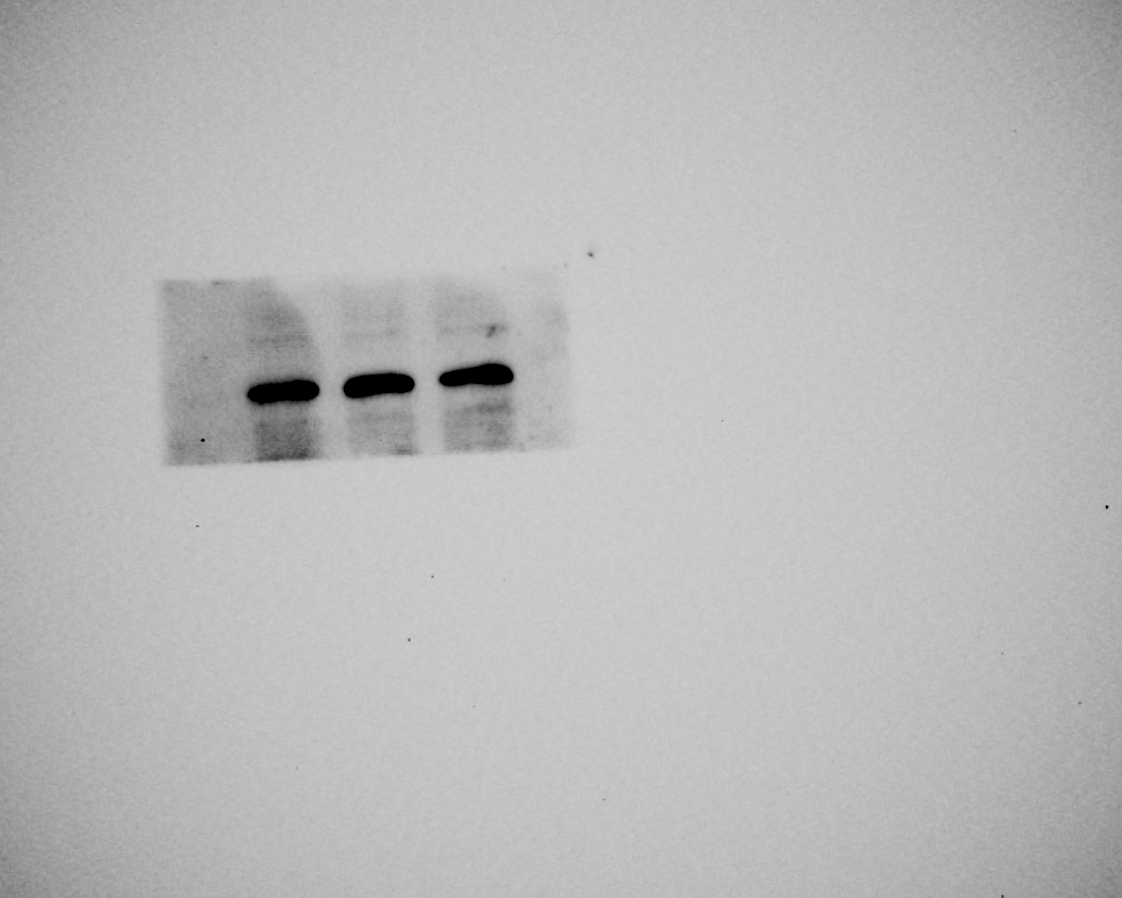

Supplement: Supplementary file 6 [file DataSheet_3.zip › Original Image of Western Blot/12.jpg]

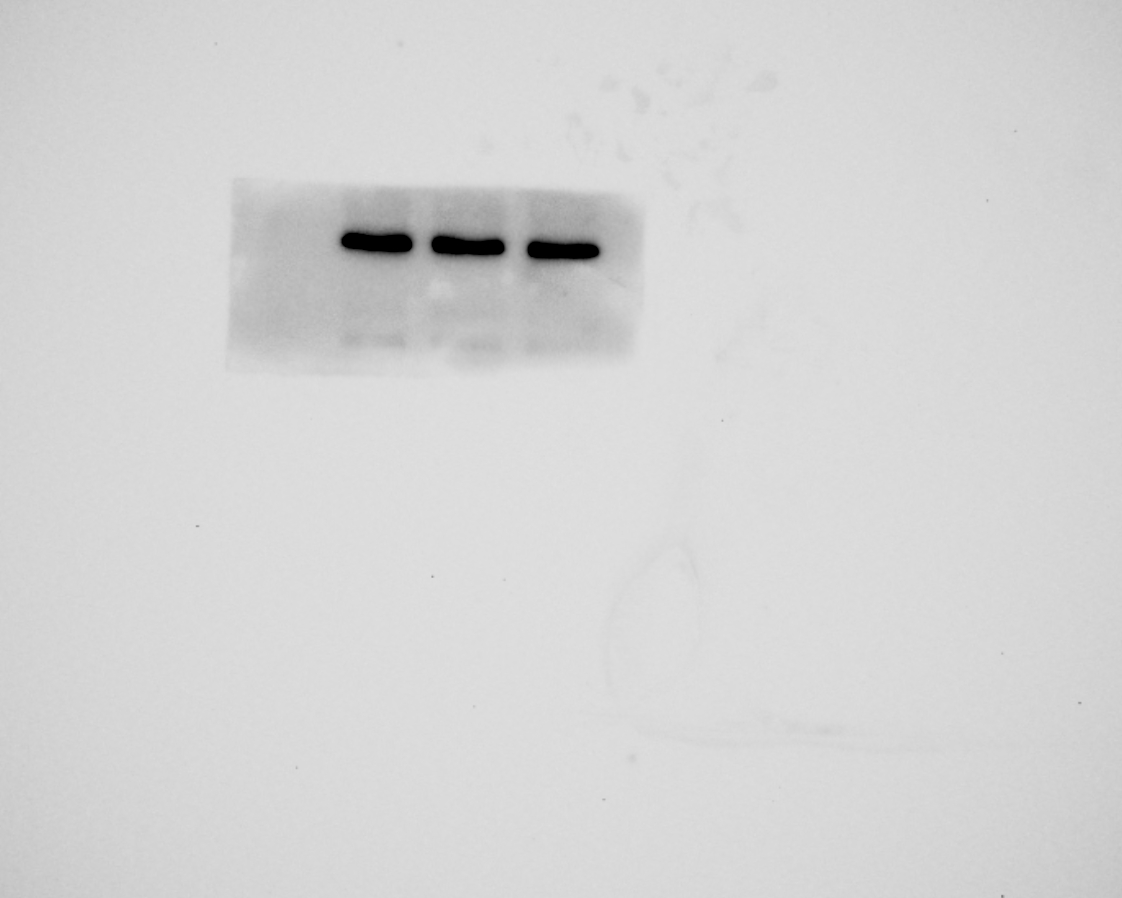

Supplement: Supplementary file 6 [file DataSheet_3.zip › Original Image of Western Blot/13.jpg]

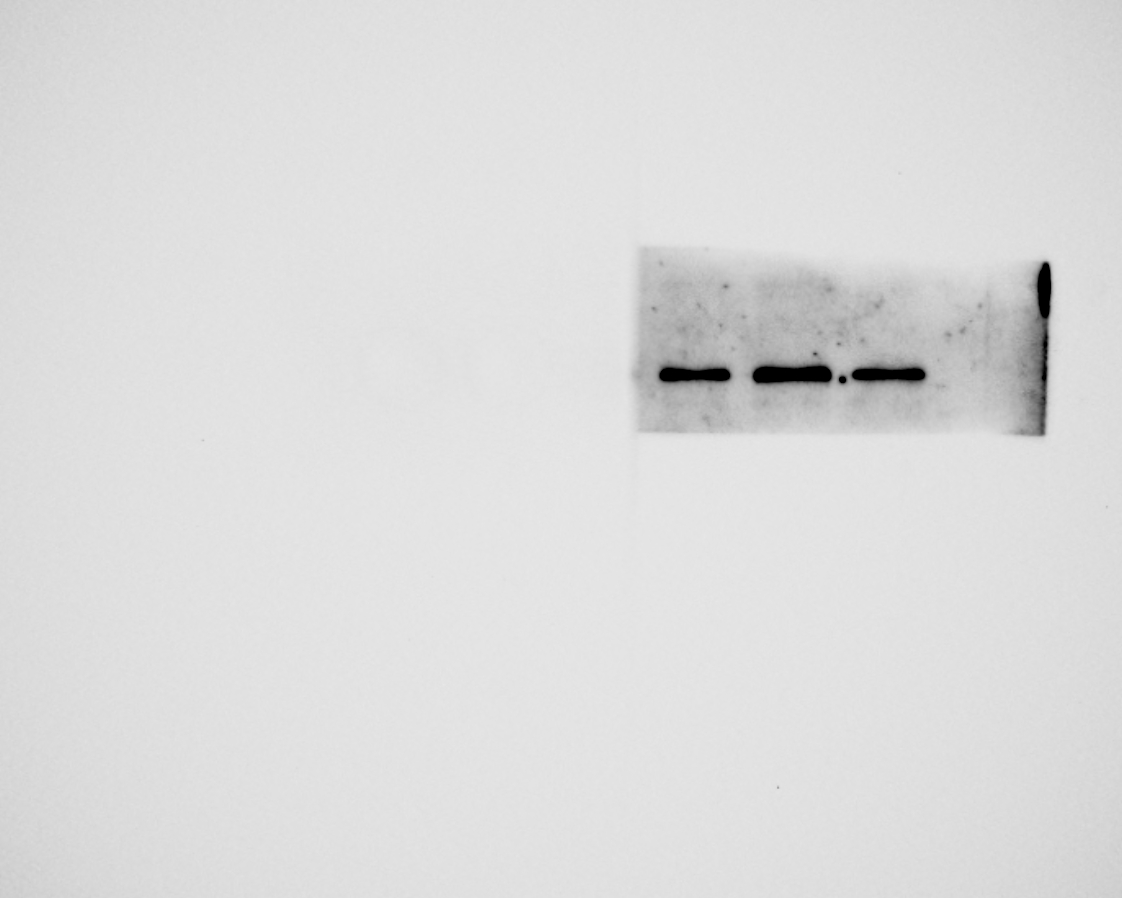

Supplement: Supplementary file 6 [file DataSheet_3.zip › Original Image of Western Blot/14.jpg]

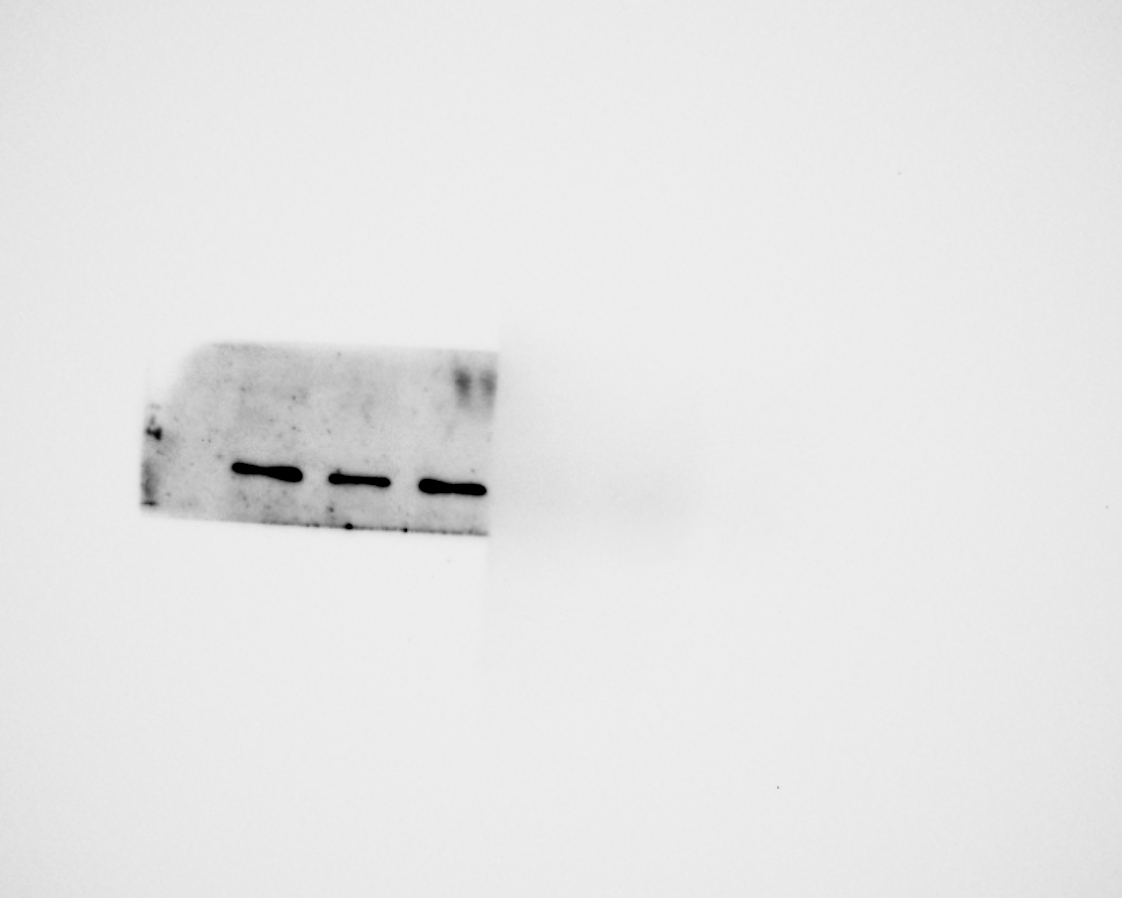

Supplement: Supplementary file 6 [file DataSheet_3.zip › Original Image of Western Blot/15.jpg]

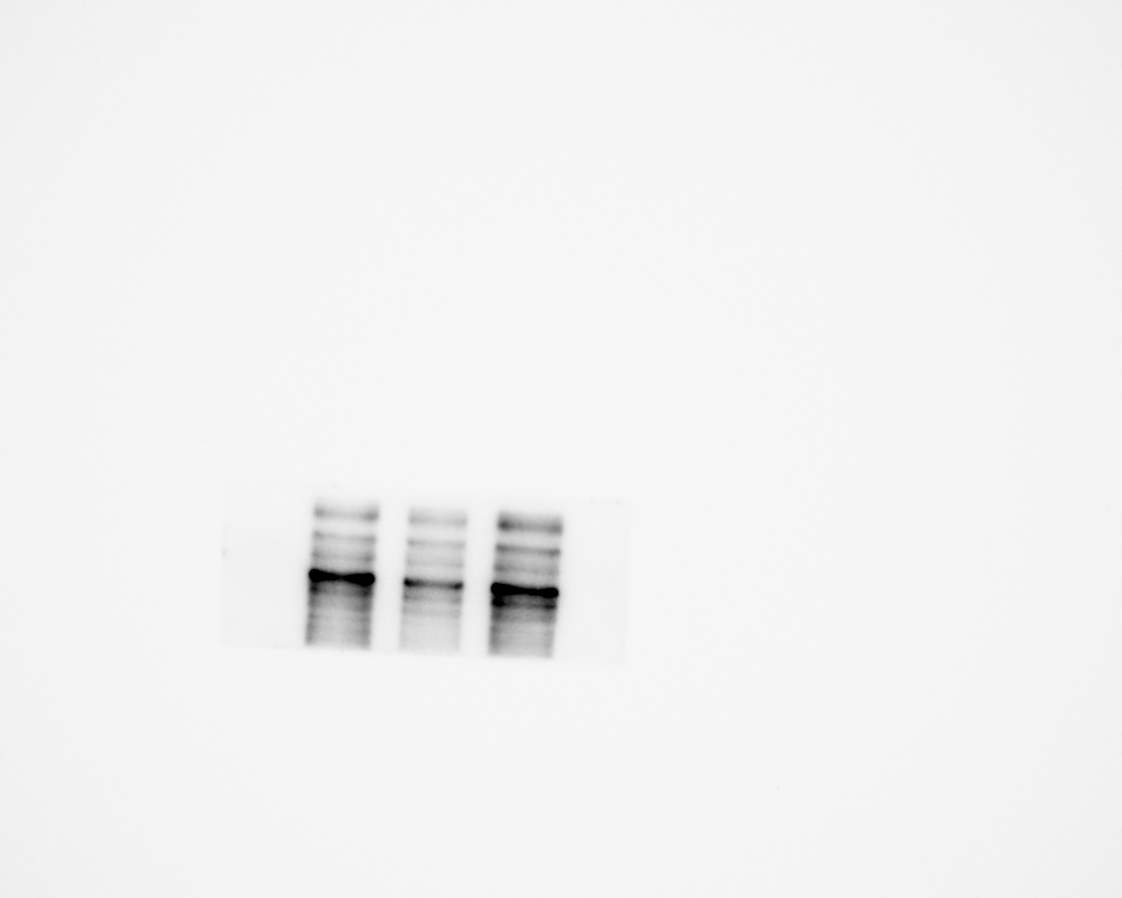

Supplement: Supplementary file 6 [file DataSheet_3.zip › Original Image of Western Blot/16.jpg]

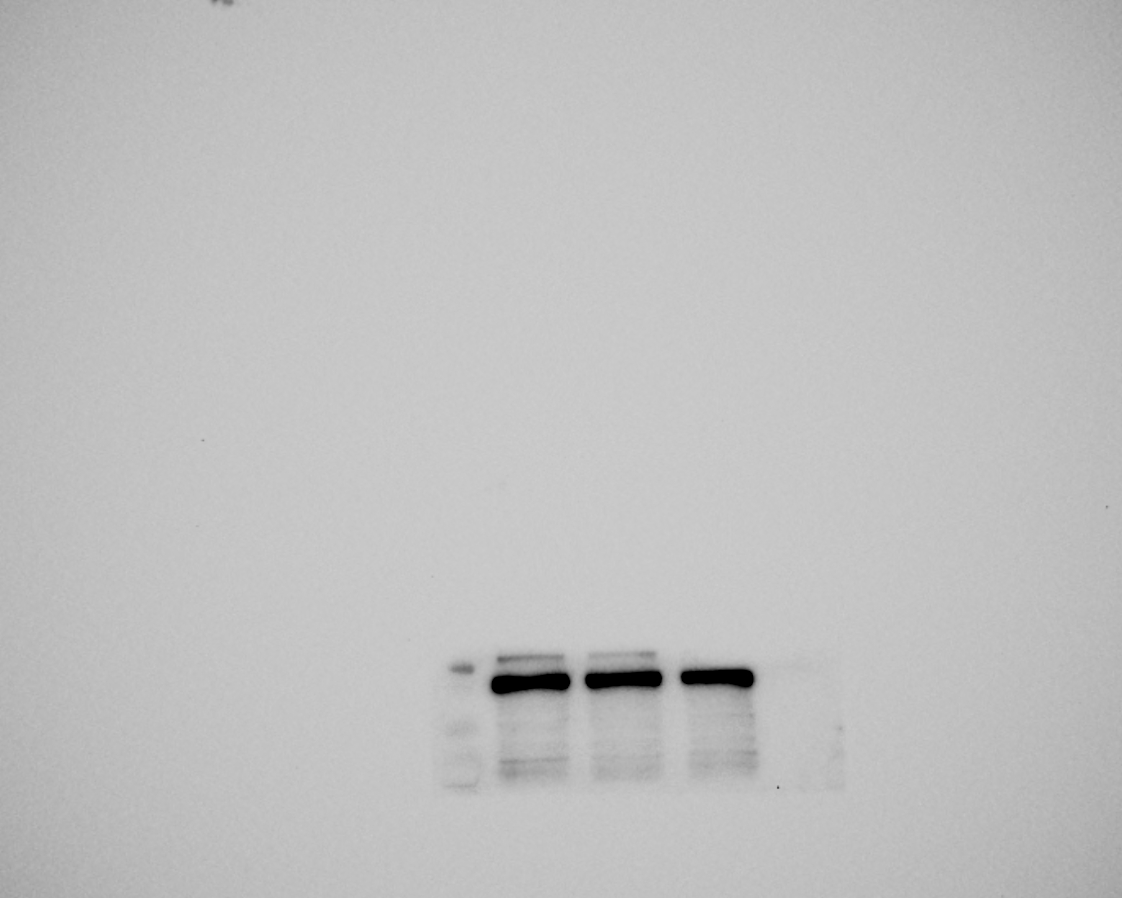

Supplement: Supplementary file 6 [file DataSheet_3.zip › Original Image of Western Blot/17.jpg]

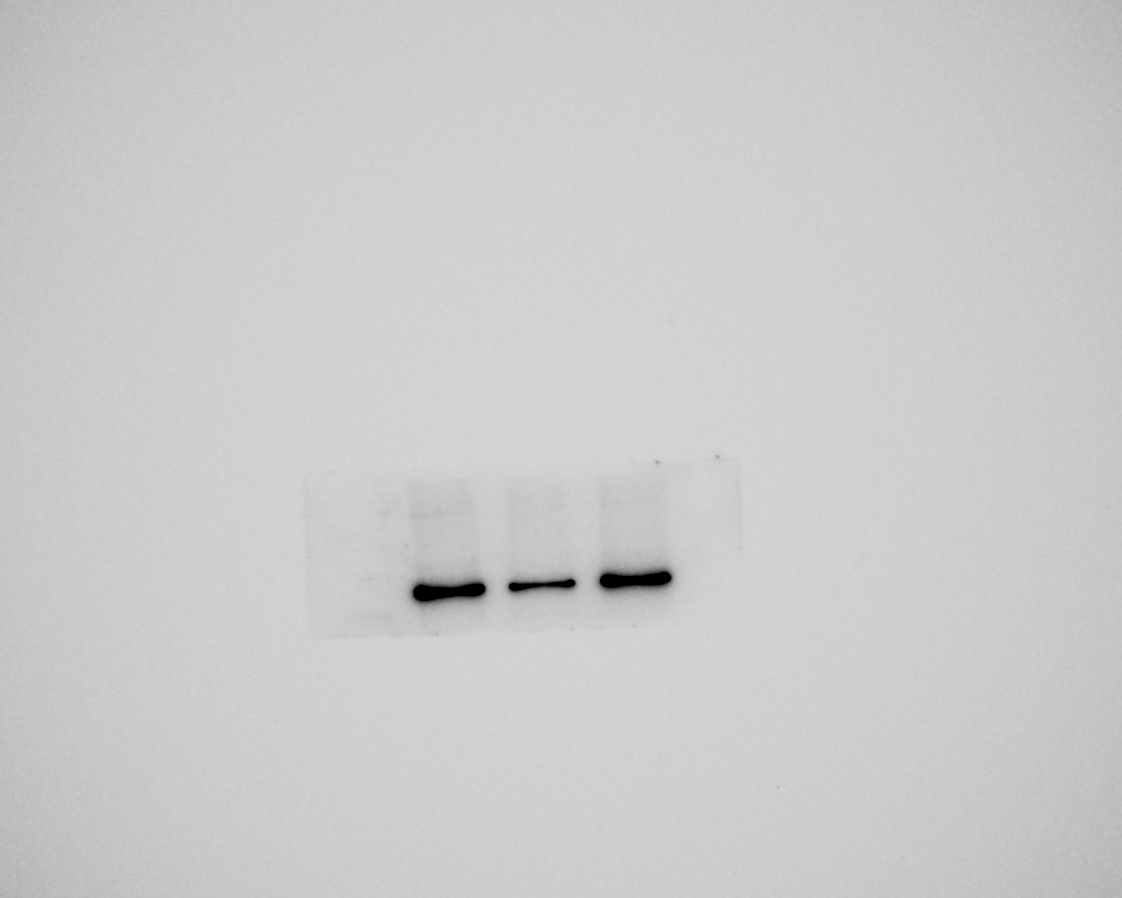

Supplement: Supplementary file 6 [file DataSheet_3.zip › Original Image of Western Blot/18.jpg]

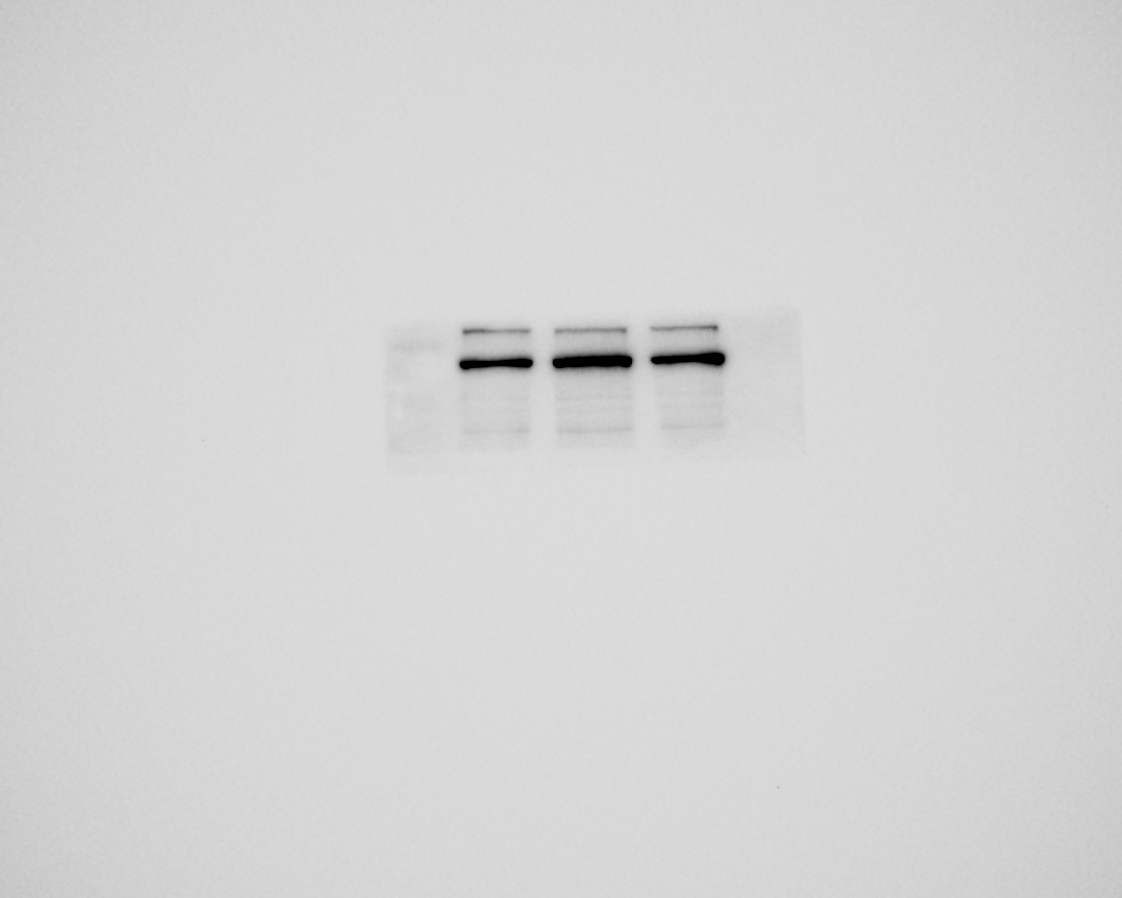

Supplement: Supplementary file 6 [file DataSheet_3.zip › Original Image of Western Blot/19.jpg]

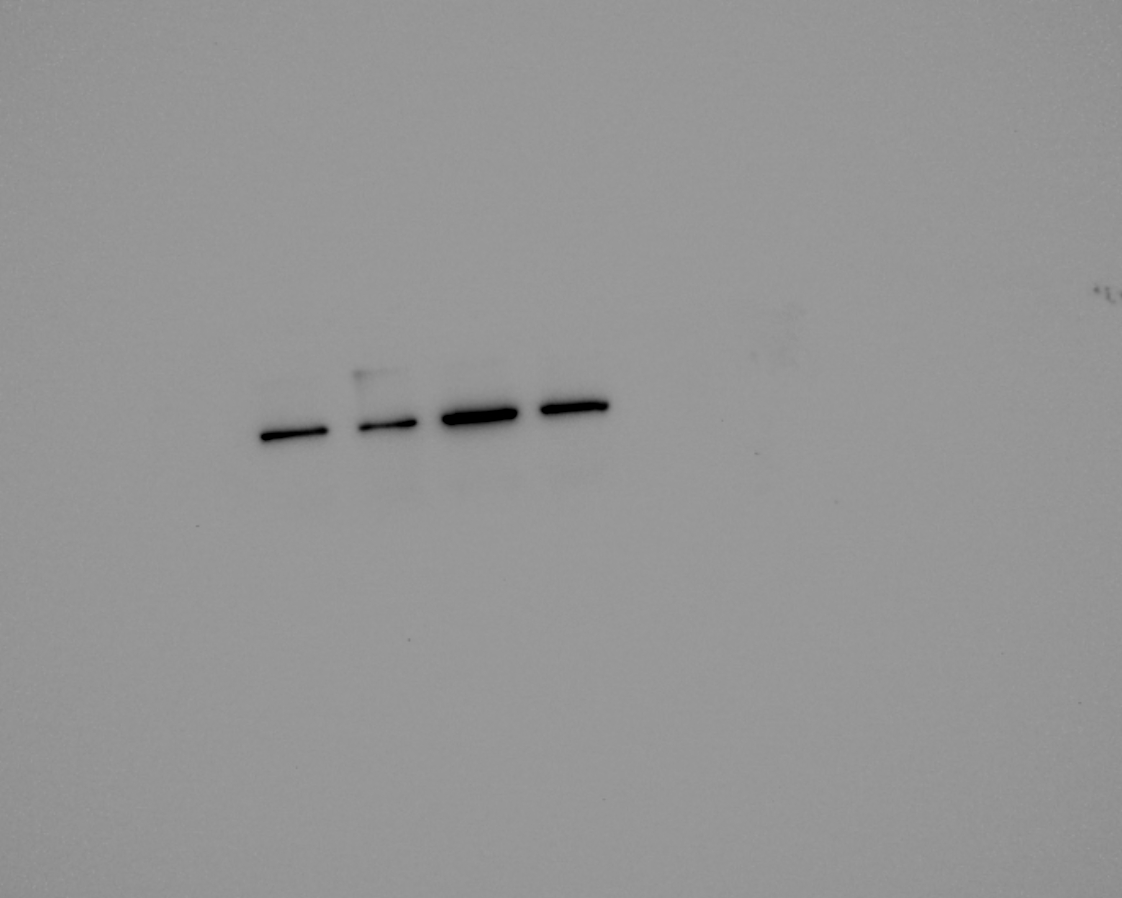

Supplement: Supplementary file 6 [file DataSheet_3.zip › Original Image of Western Blot/2.jpg]

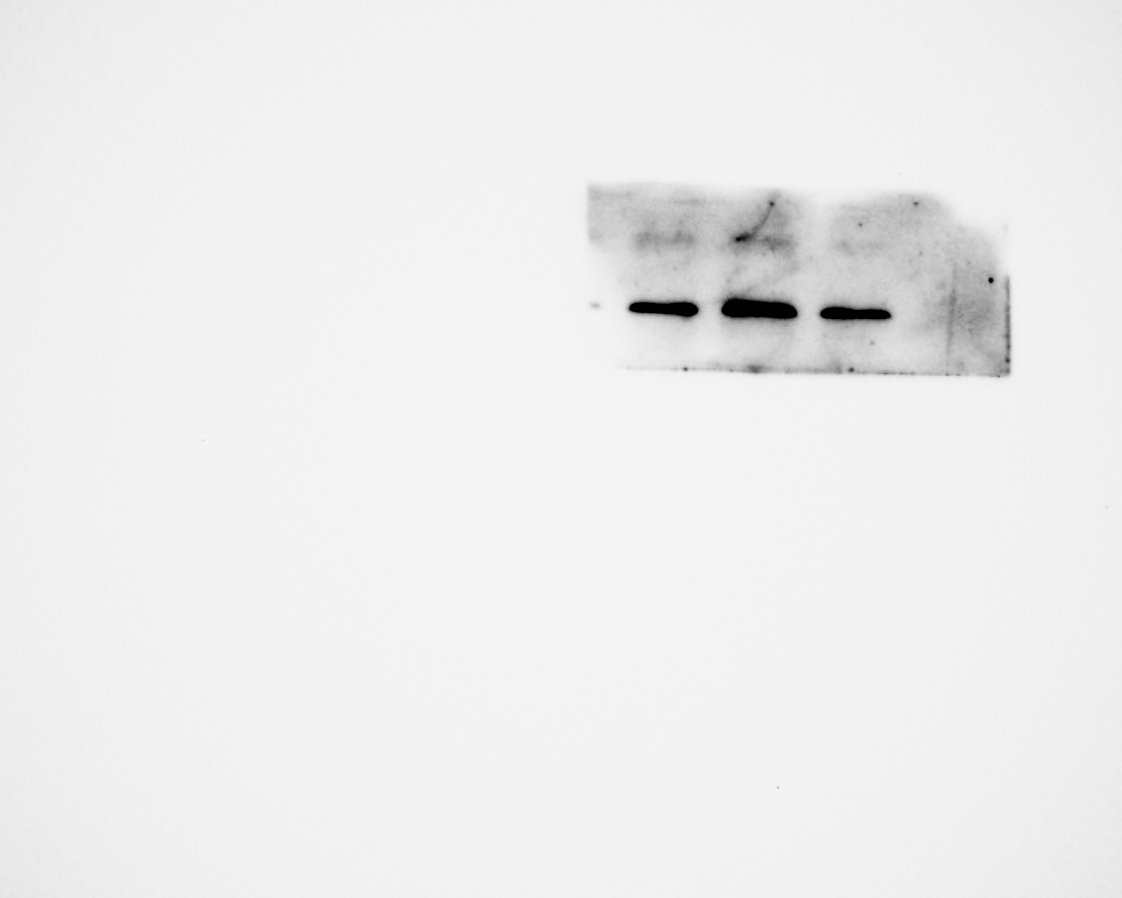

Supplement: Supplementary file 6 [file DataSheet_3.zip › Original Image of Western Blot/20.jpg]

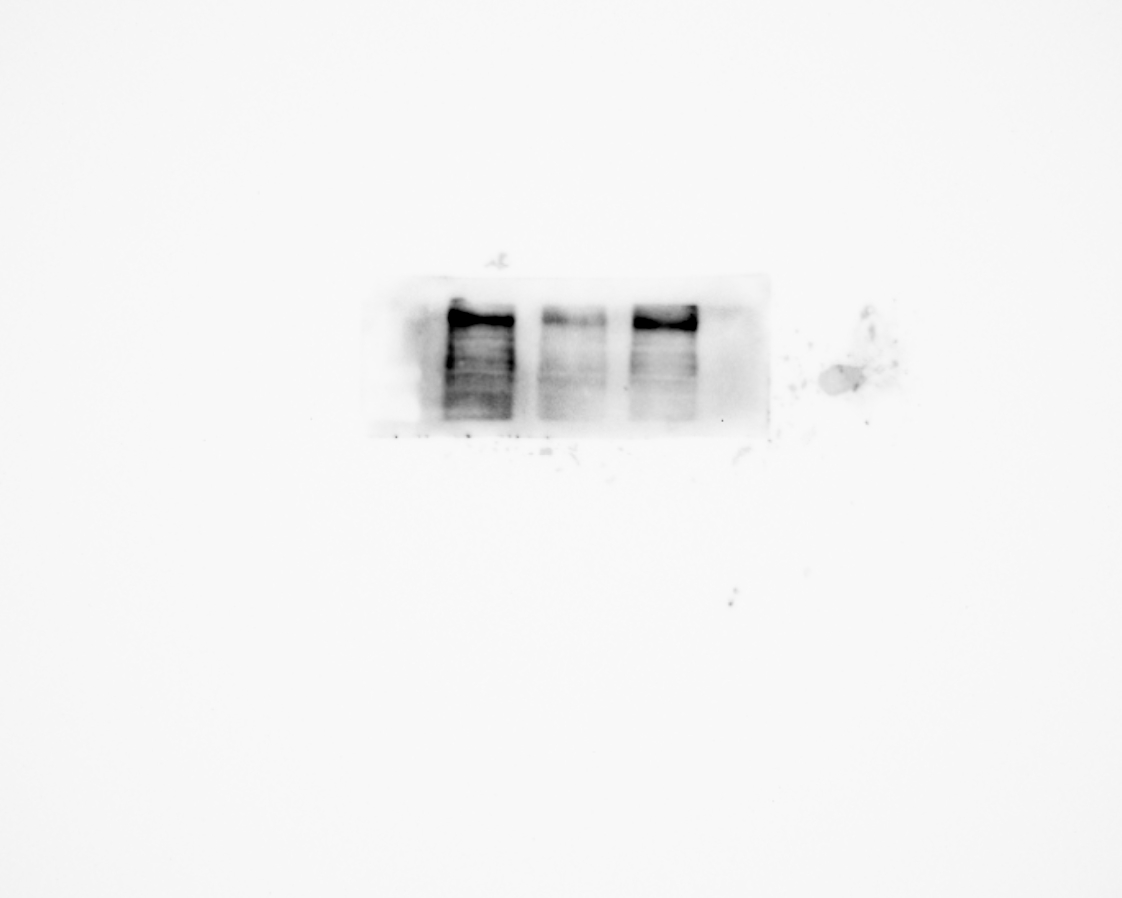

Supplement: Supplementary file 6 [file DataSheet_3.zip › Original Image of Western Blot/21.jpg]

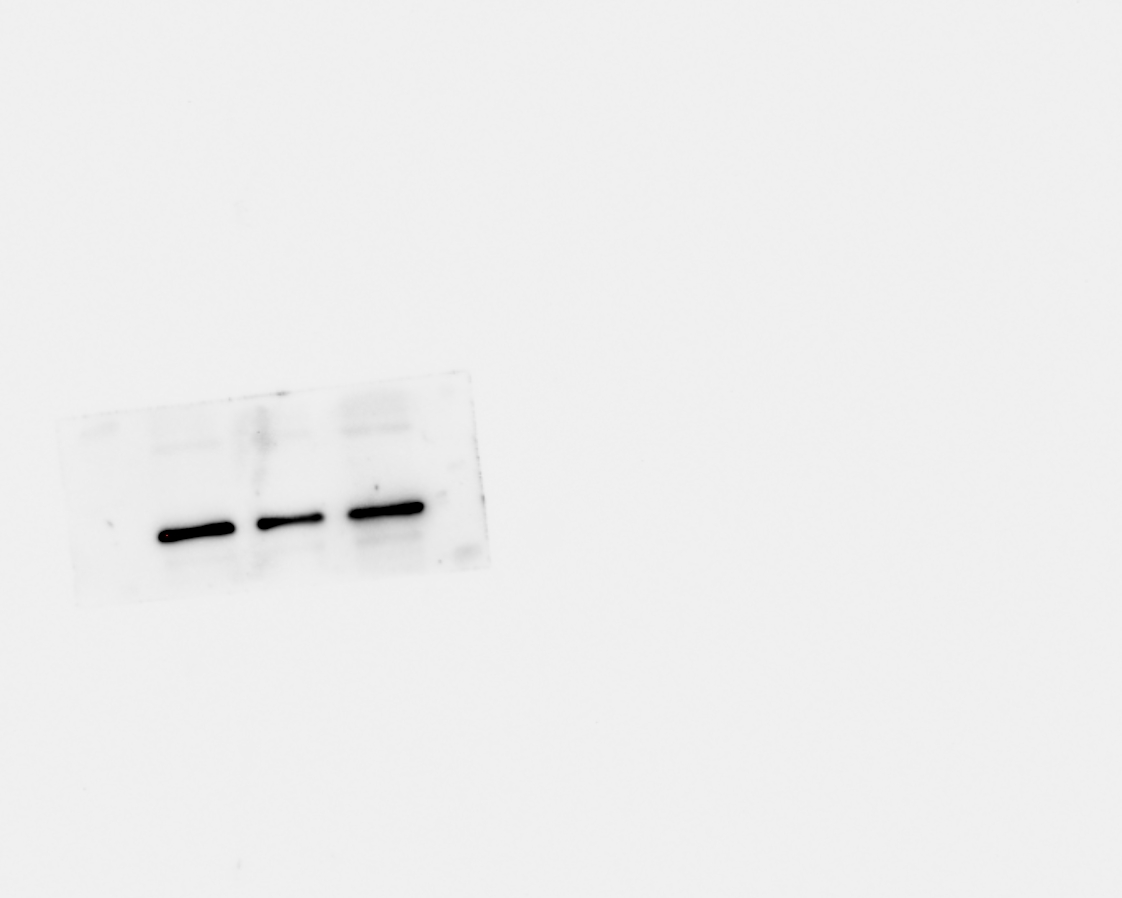

Supplement: Supplementary file 6 [file DataSheet_3.zip › Original Image of Western Blot/22.jpg]

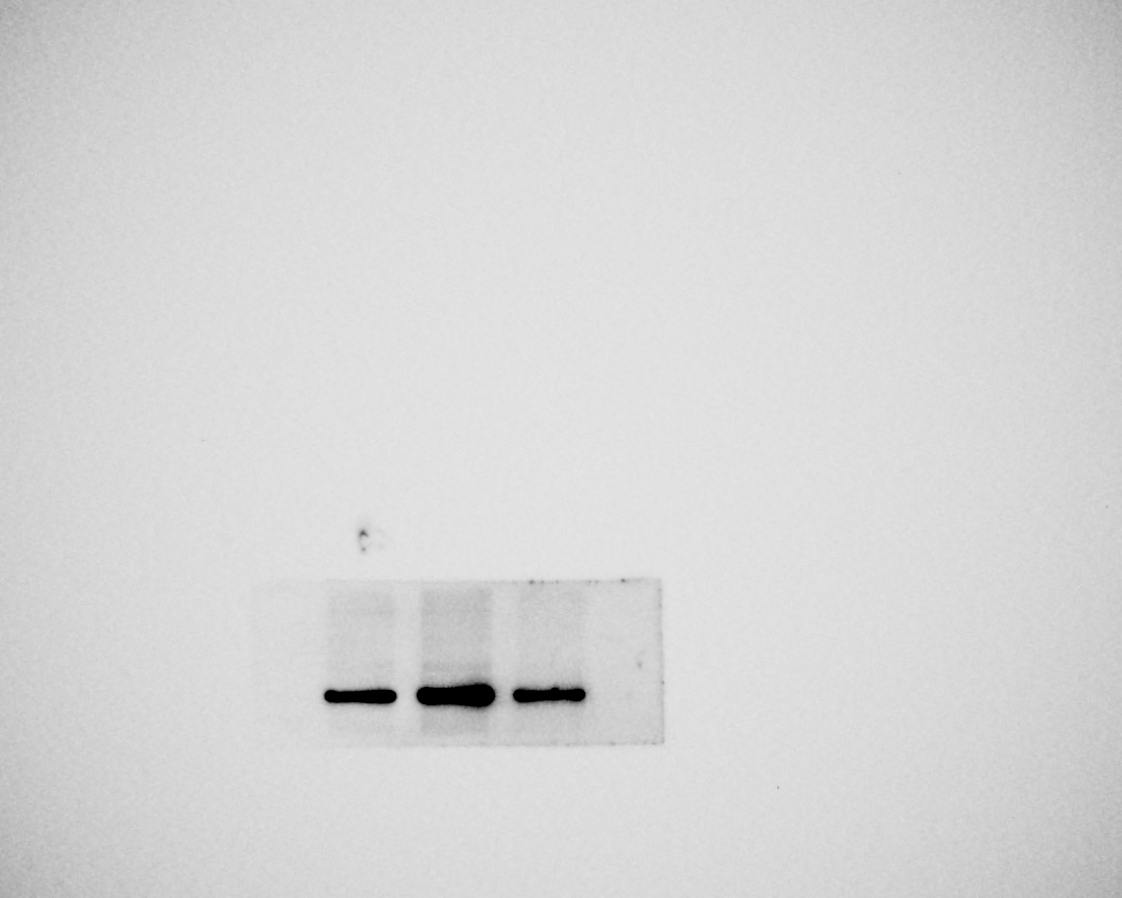

Supplement: Supplementary file 6 [file DataSheet_3.zip › Original Image of Western Blot/23.jpg]

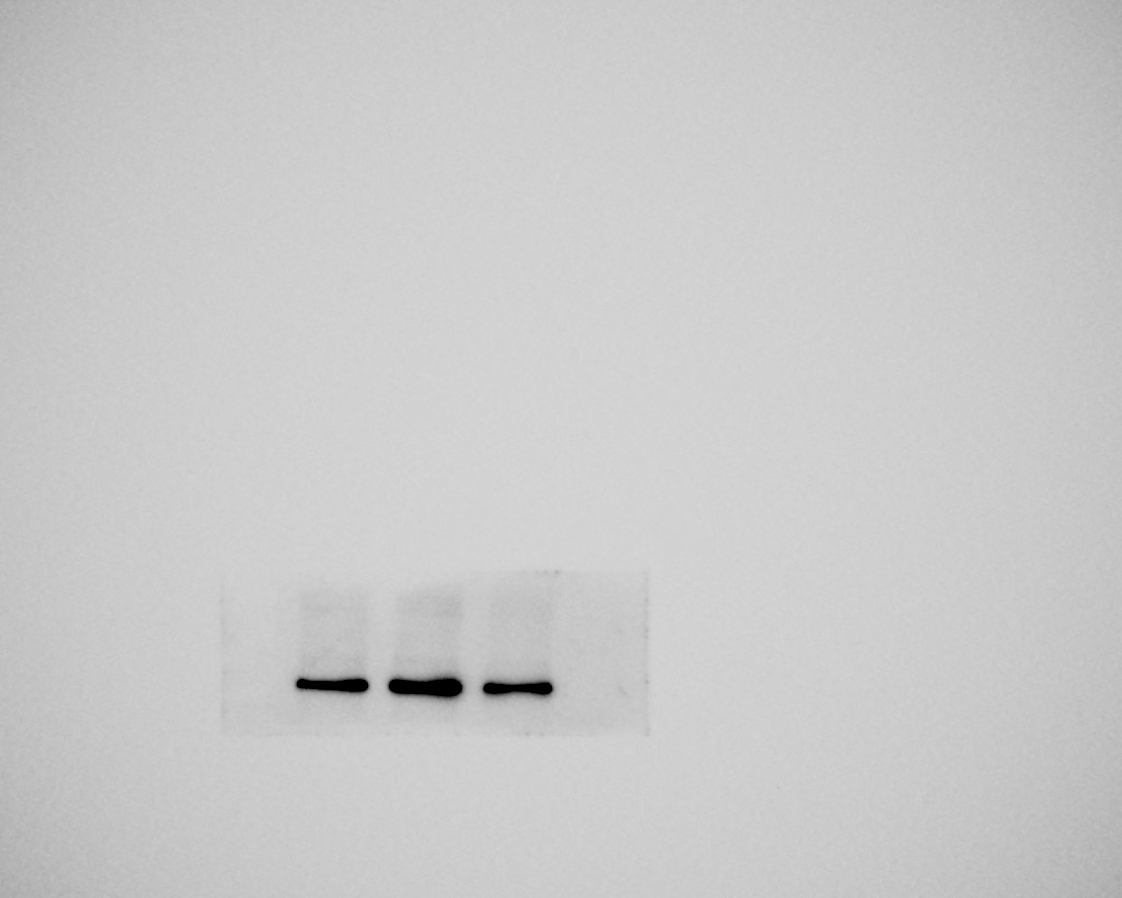

Supplement: Supplementary file 6 [file DataSheet_3.zip › Original Image of Western Blot/24.jpg]

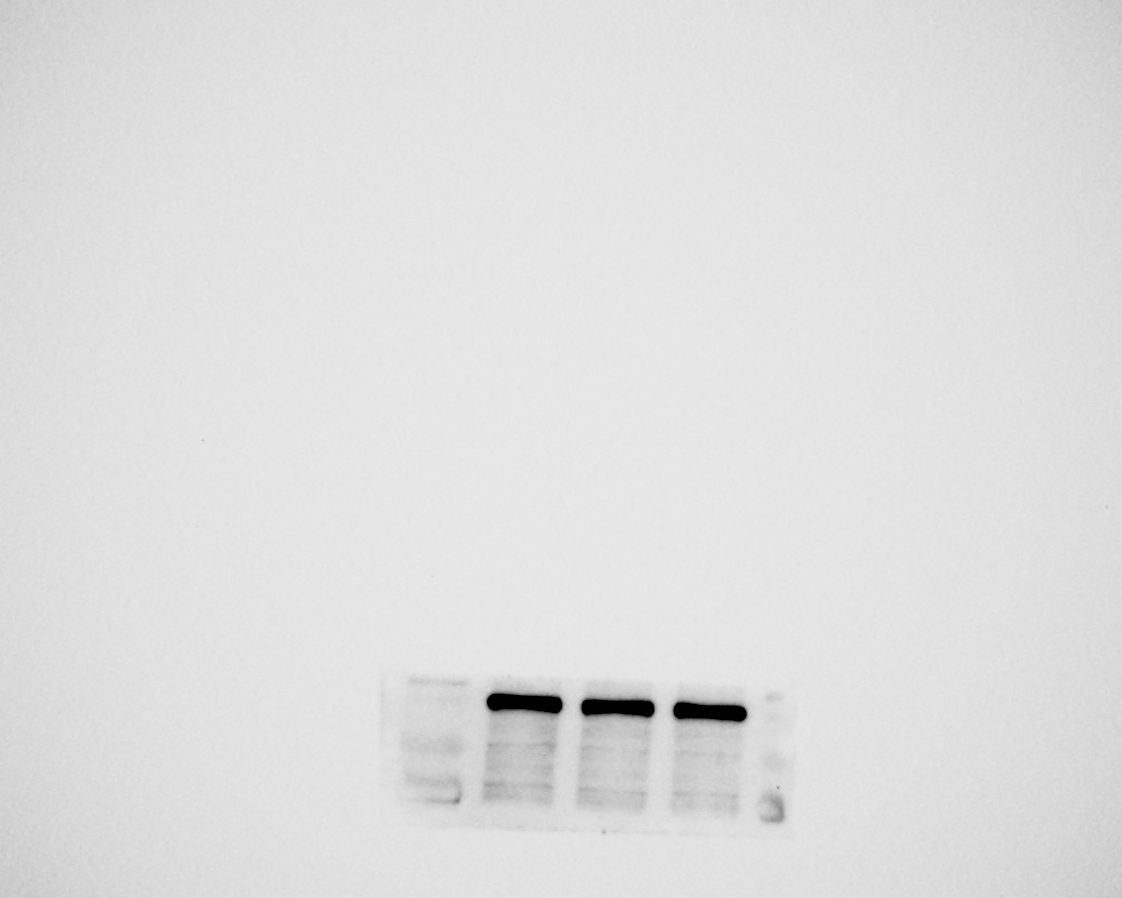

Supplement: Supplementary file 6 [file DataSheet_3.zip › Original Image of Western Blot/25.jpg]

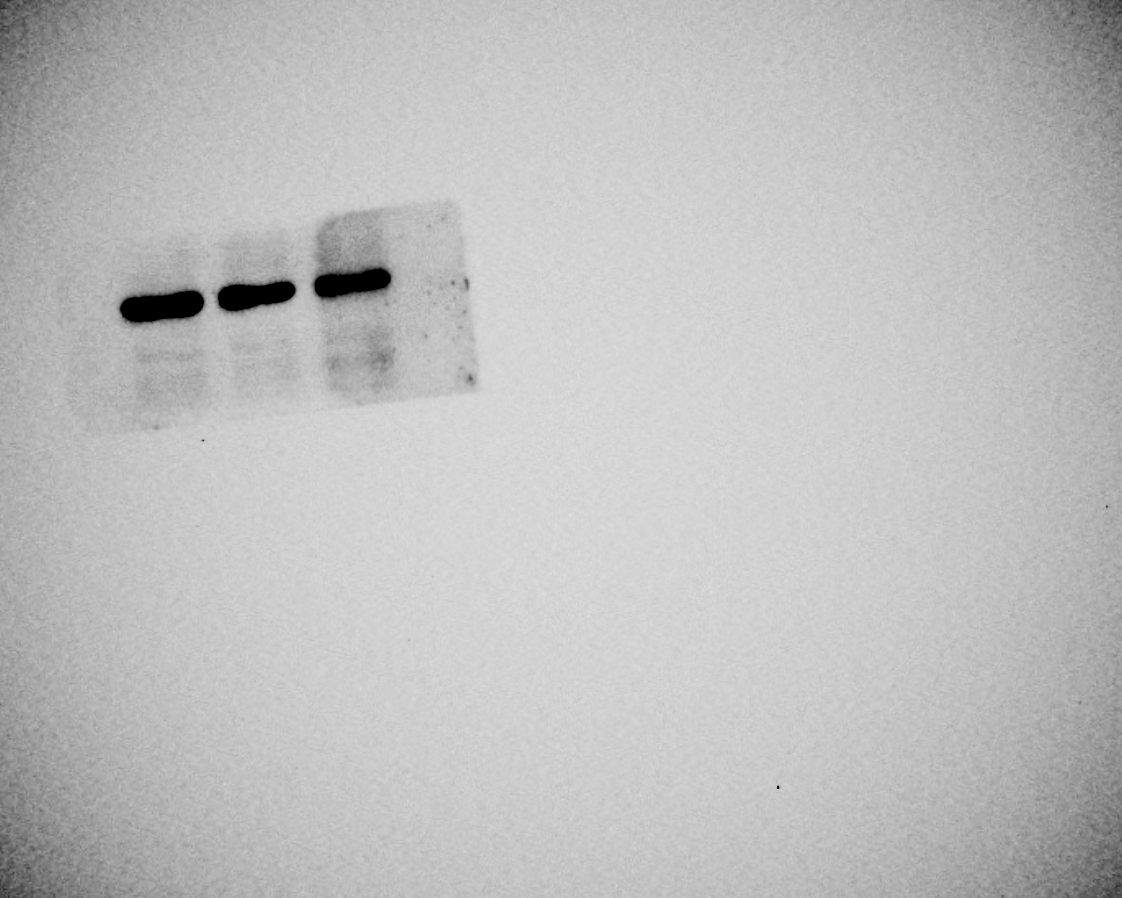

Supplement: Supplementary file 6 [file DataSheet_3.zip › Original Image of Western Blot/26.jpg]

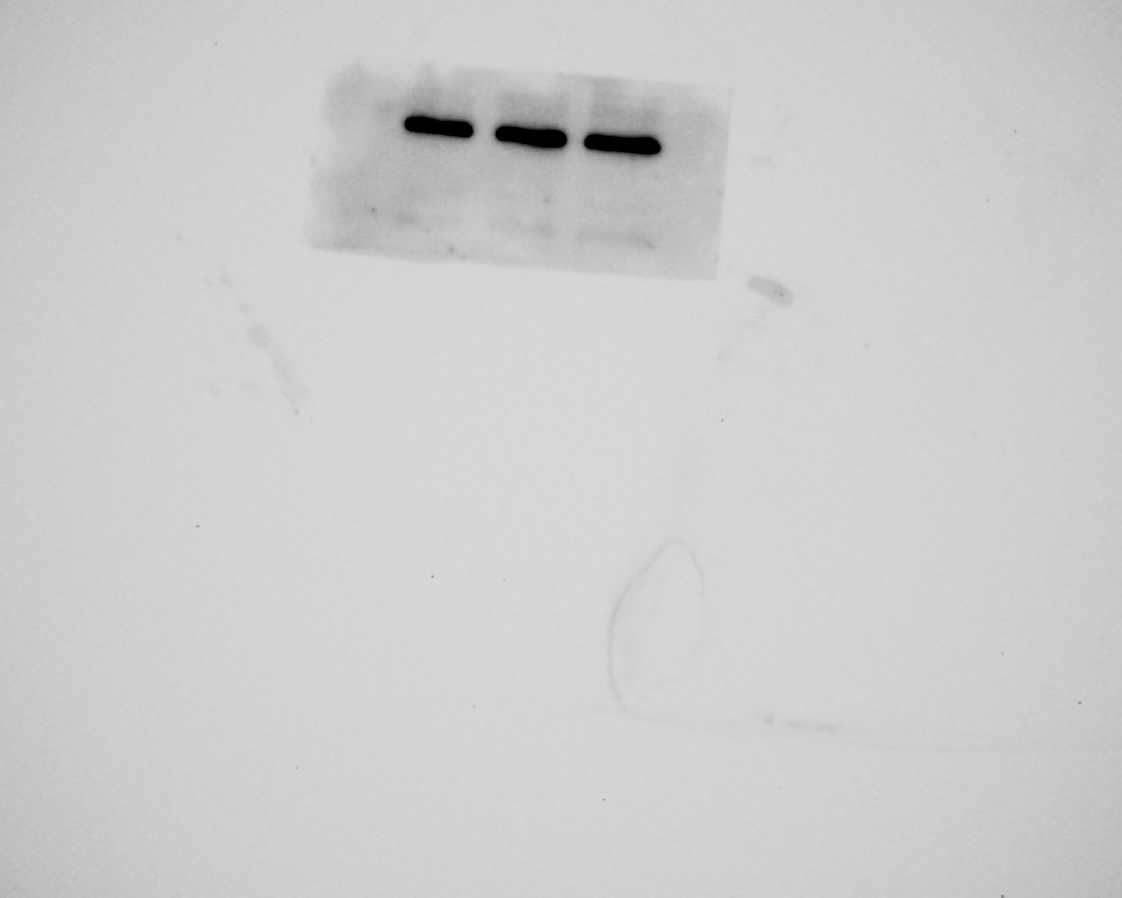

Supplement: Supplementary file 6 [file DataSheet_3.zip › Original Image of Western Blot/27.jpg]

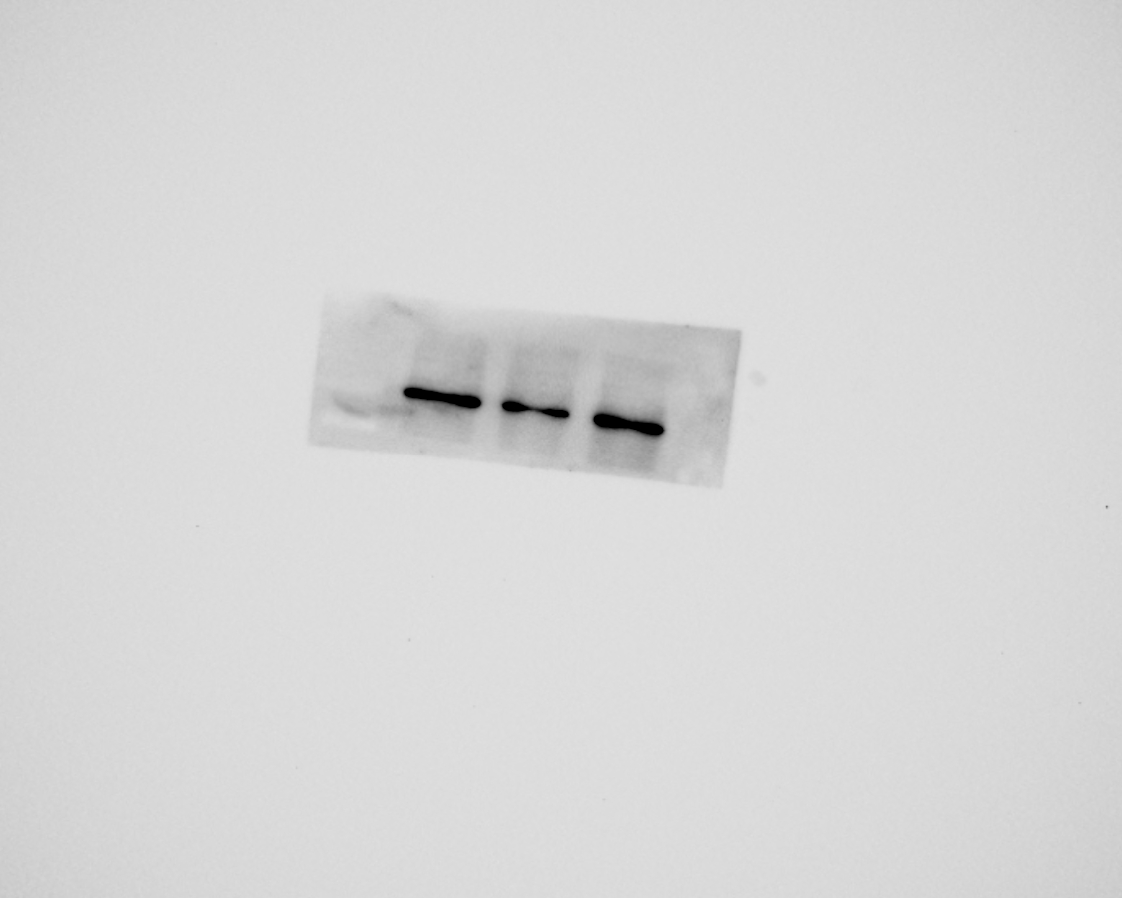

Supplement: Supplementary file 6 [file DataSheet_3.zip › Original Image of Western Blot/28.jpg]

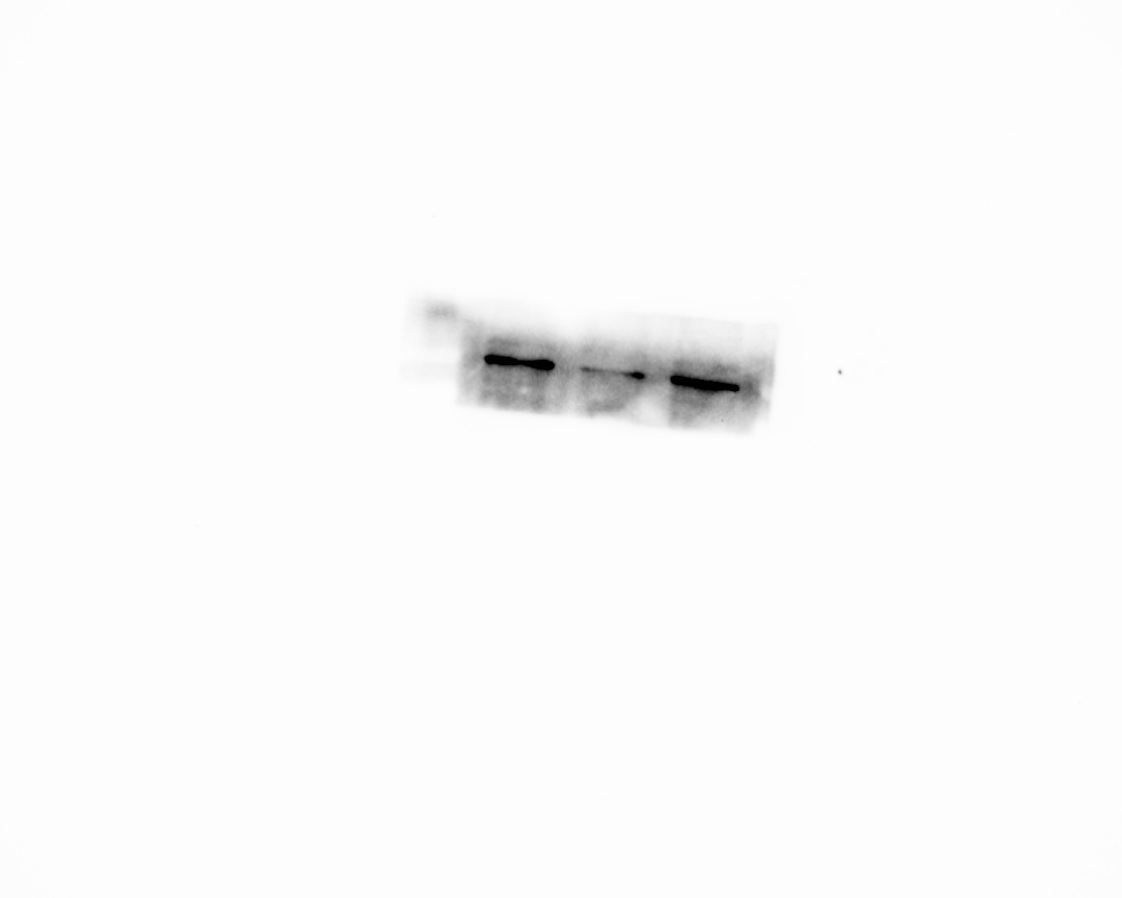

Supplement: Supplementary file 6 [file DataSheet_3.zip › Original Image of Western Blot/29.jpg]

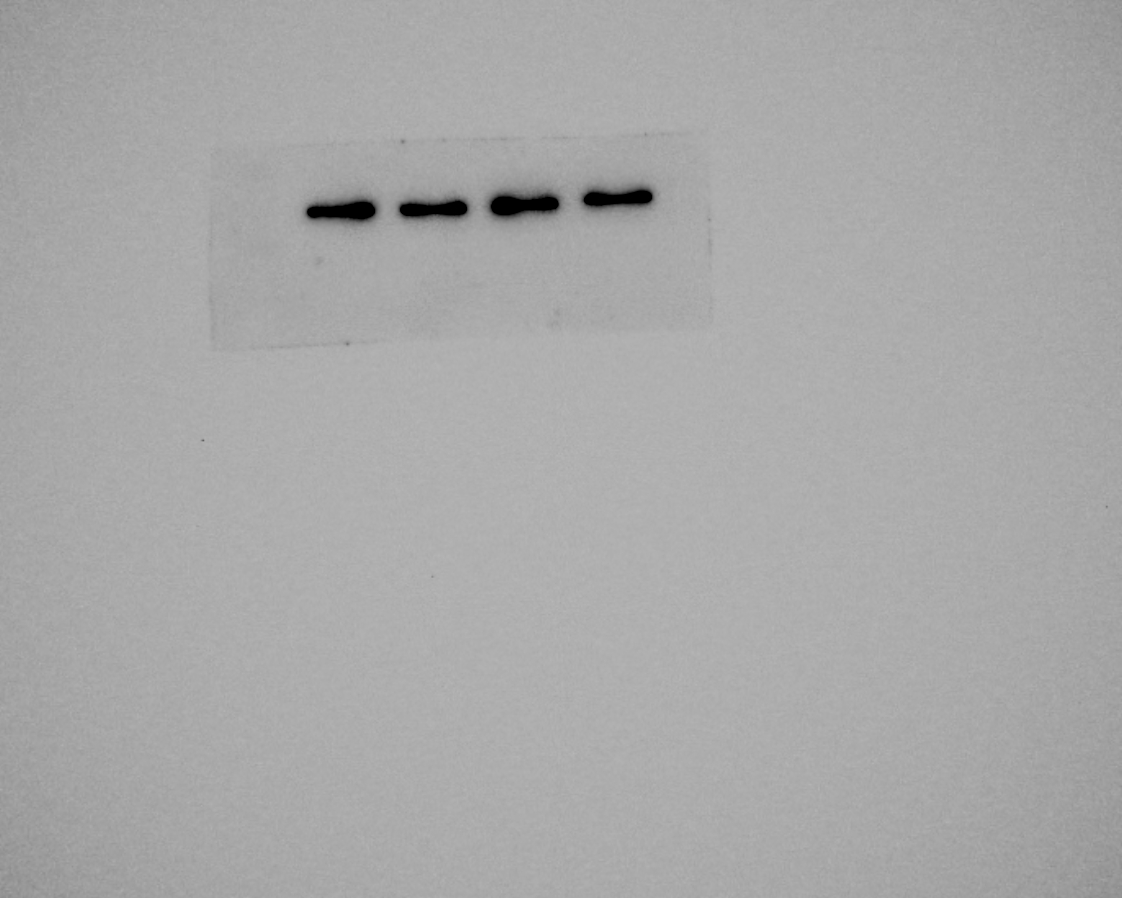

Supplement: Supplementary file 6 [file DataSheet_3.zip › Original Image of Western Blot/3.jpg]

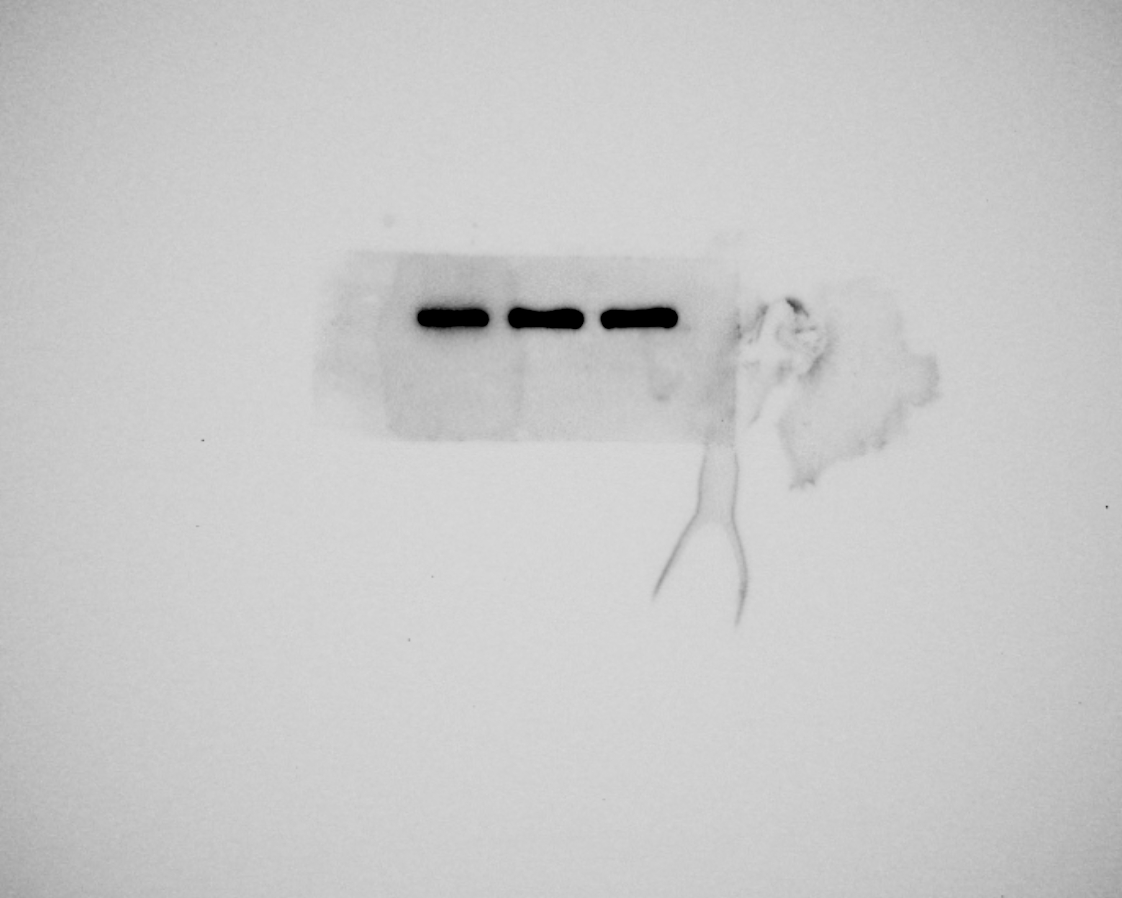

Supplement: Supplementary file 6 [file DataSheet_3.zip › Original Image of Western Blot/30.jpg]

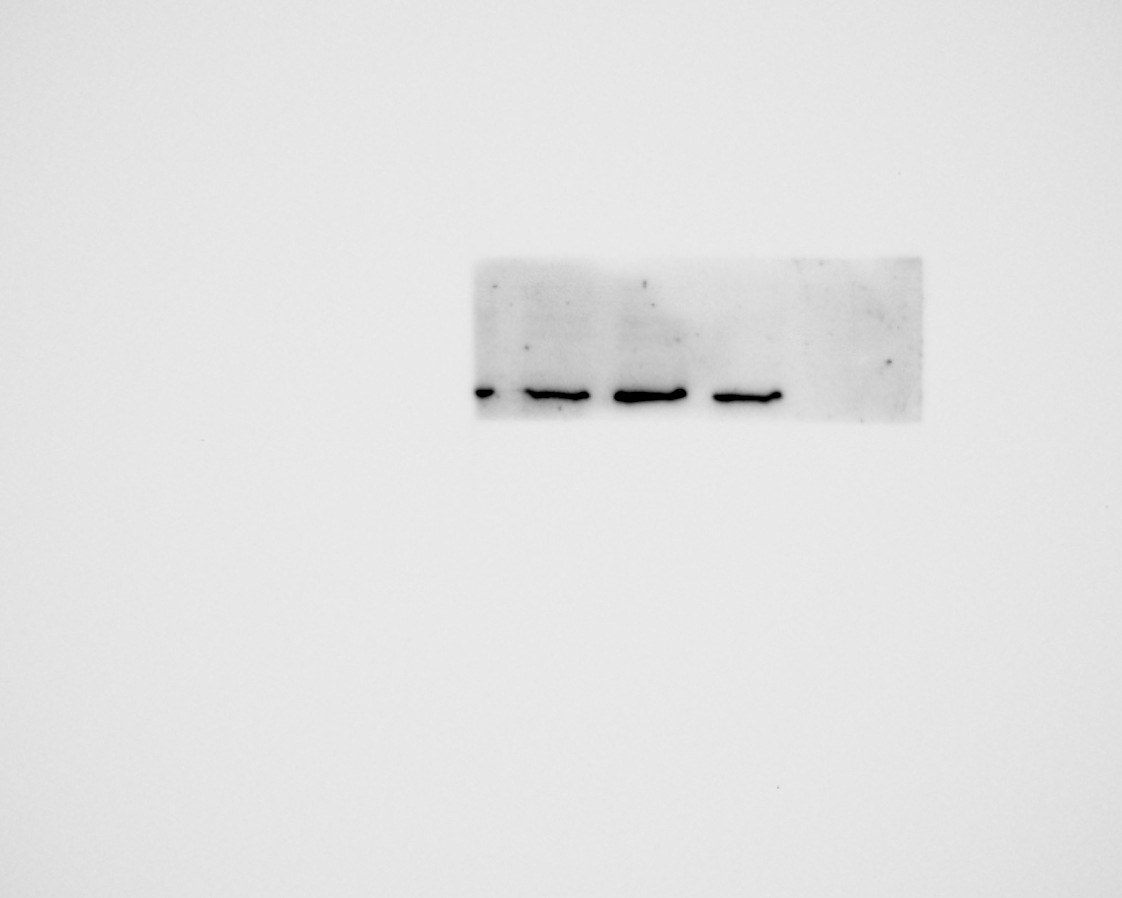

Supplement: Supplementary file 6 [file DataSheet_3.zip › Original Image of Western Blot/31.jpg]

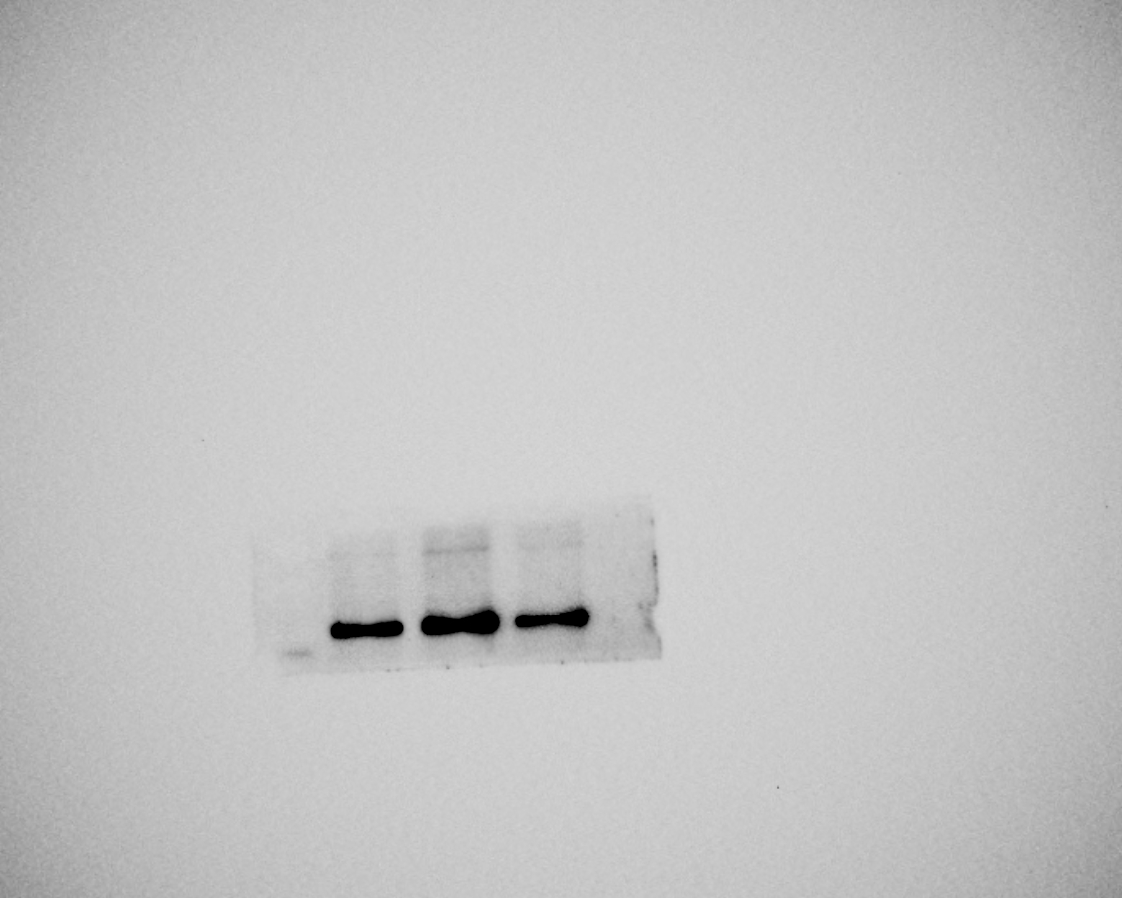

Supplement: Supplementary file 6 [file DataSheet_3.zip › Original Image of Western Blot/32.jpg]

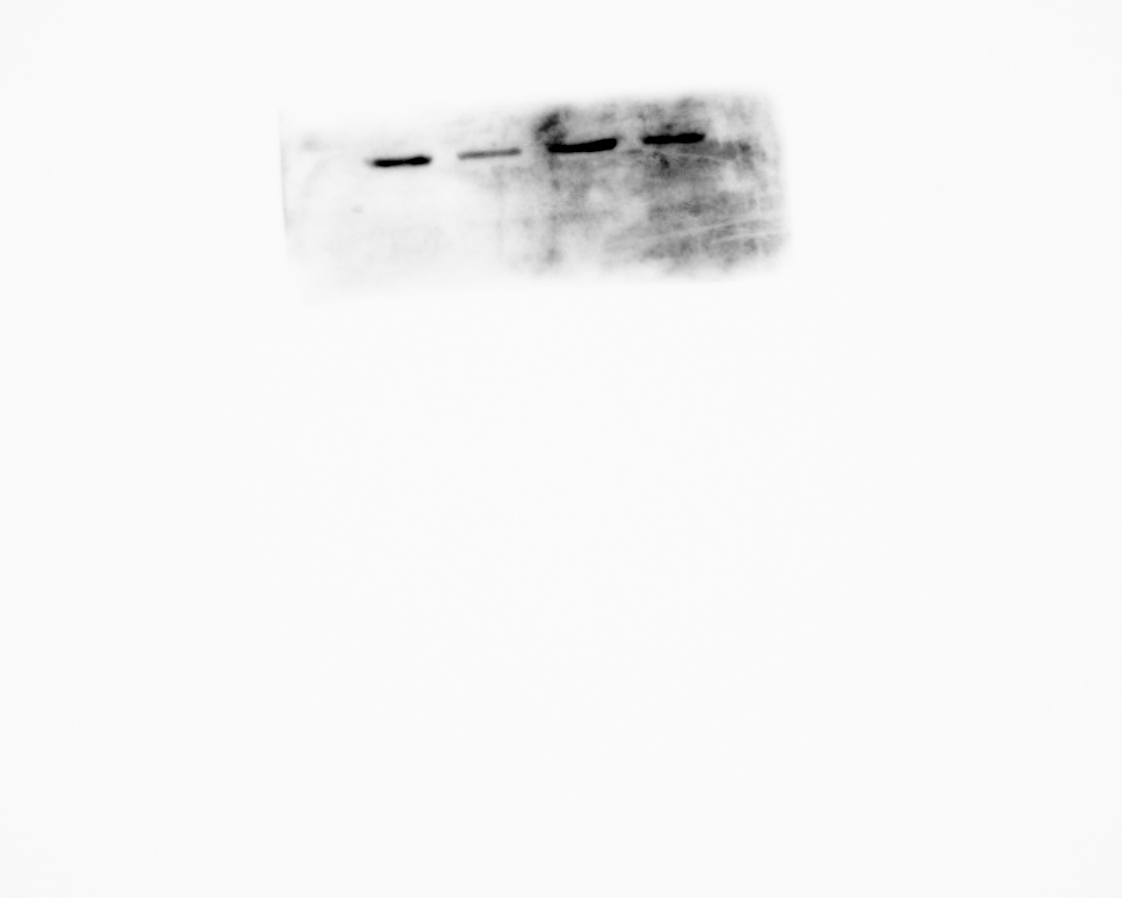

Supplement: Supplementary file 6 [file DataSheet_3.zip › Original Image of Western Blot/4.jpg]

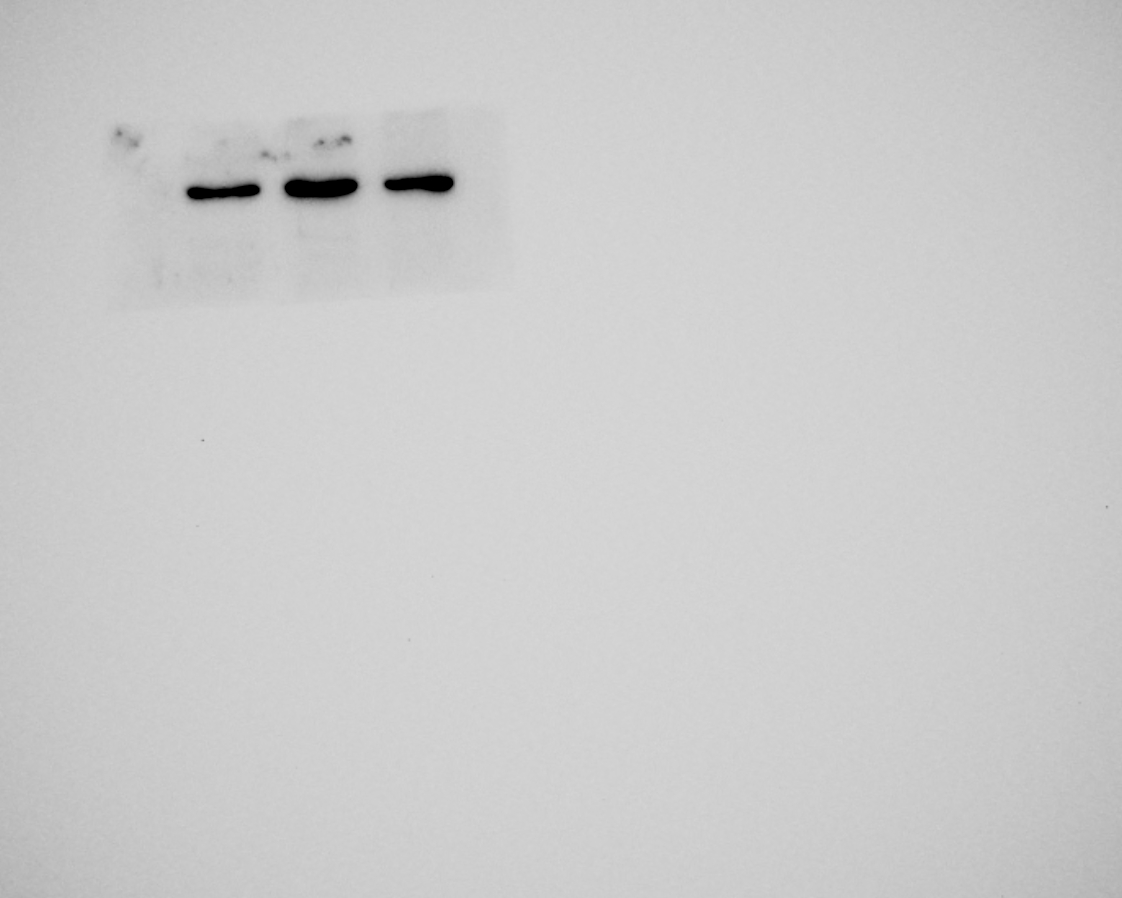

Supplement: Supplementary file 6 [file DataSheet_3.zip › Original Image of Western Blot/5.jpg]

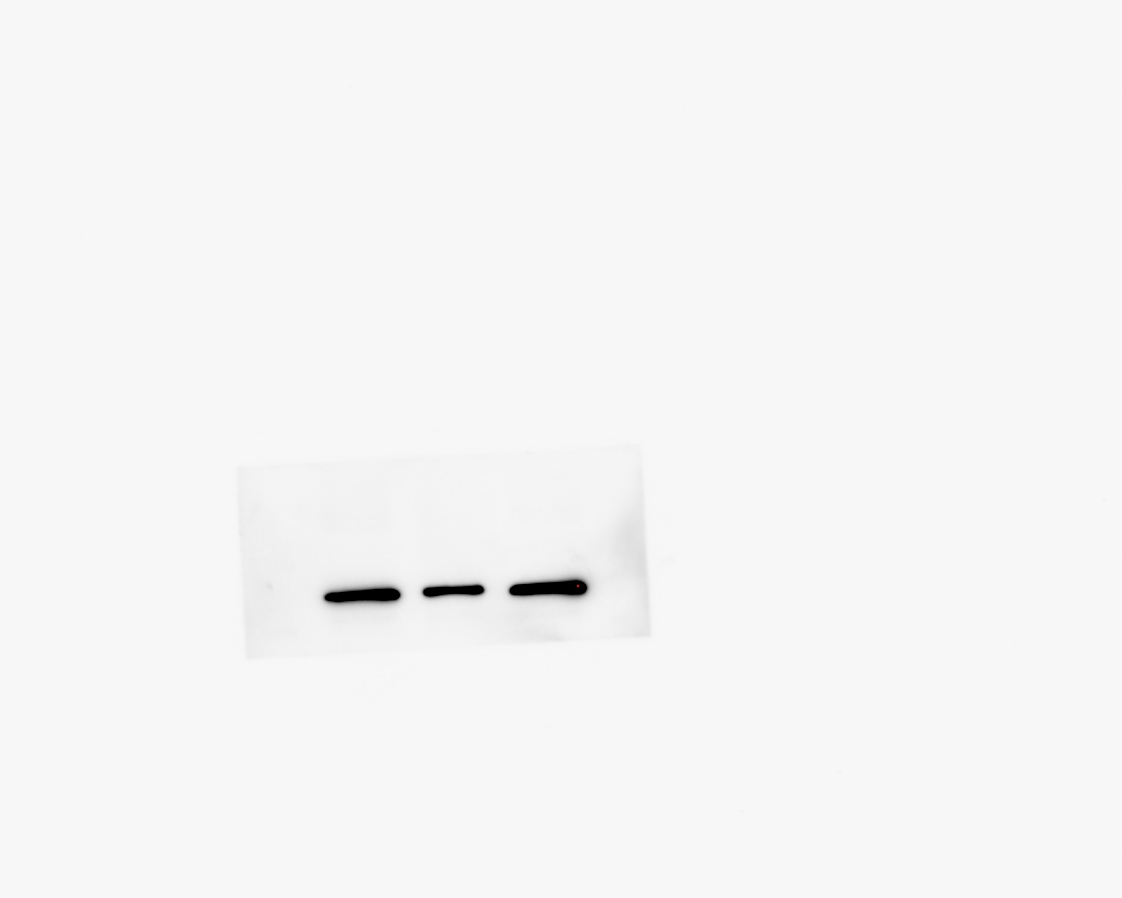

Supplement: Supplementary file 6 [file DataSheet_3.zip › Original Image of Western Blot/6.jpg]

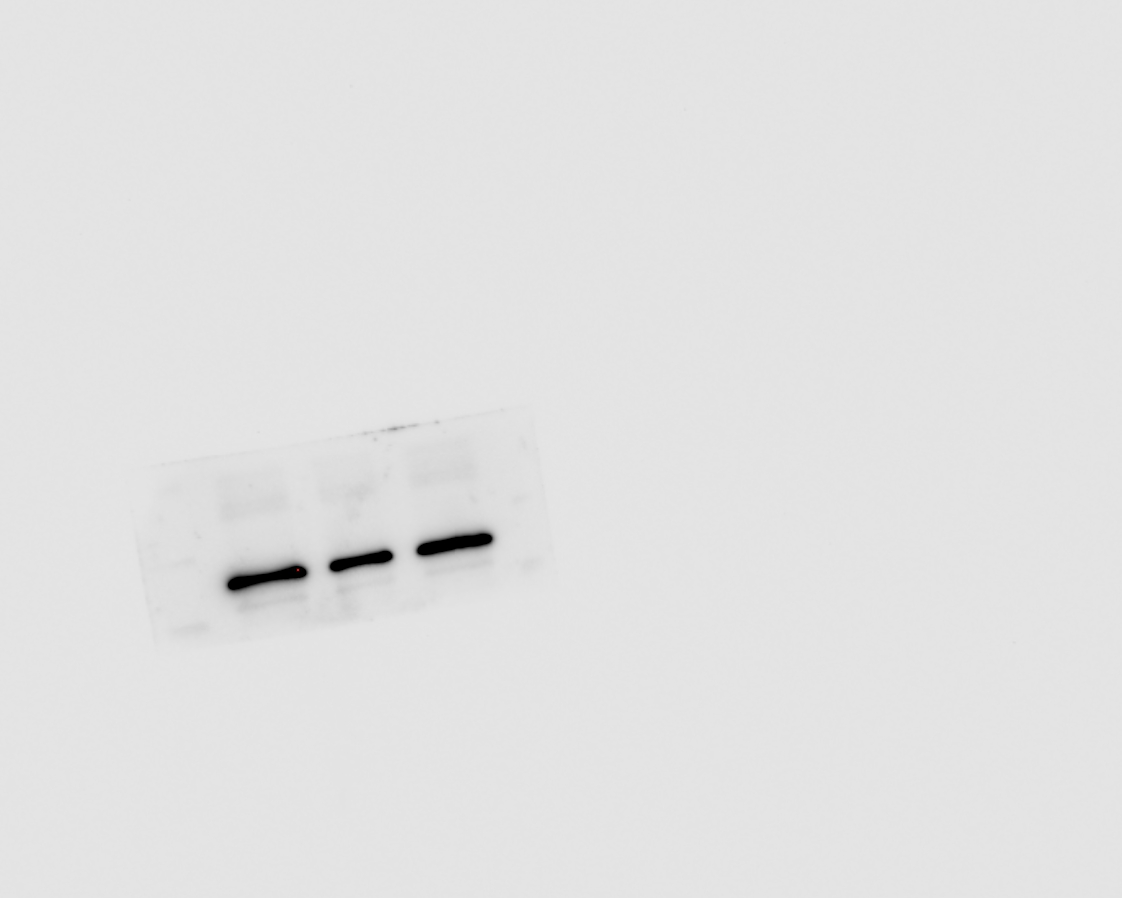

Supplement: Supplementary file 6 [file DataSheet_3.zip › Original Image of Western Blot/7.jpg]

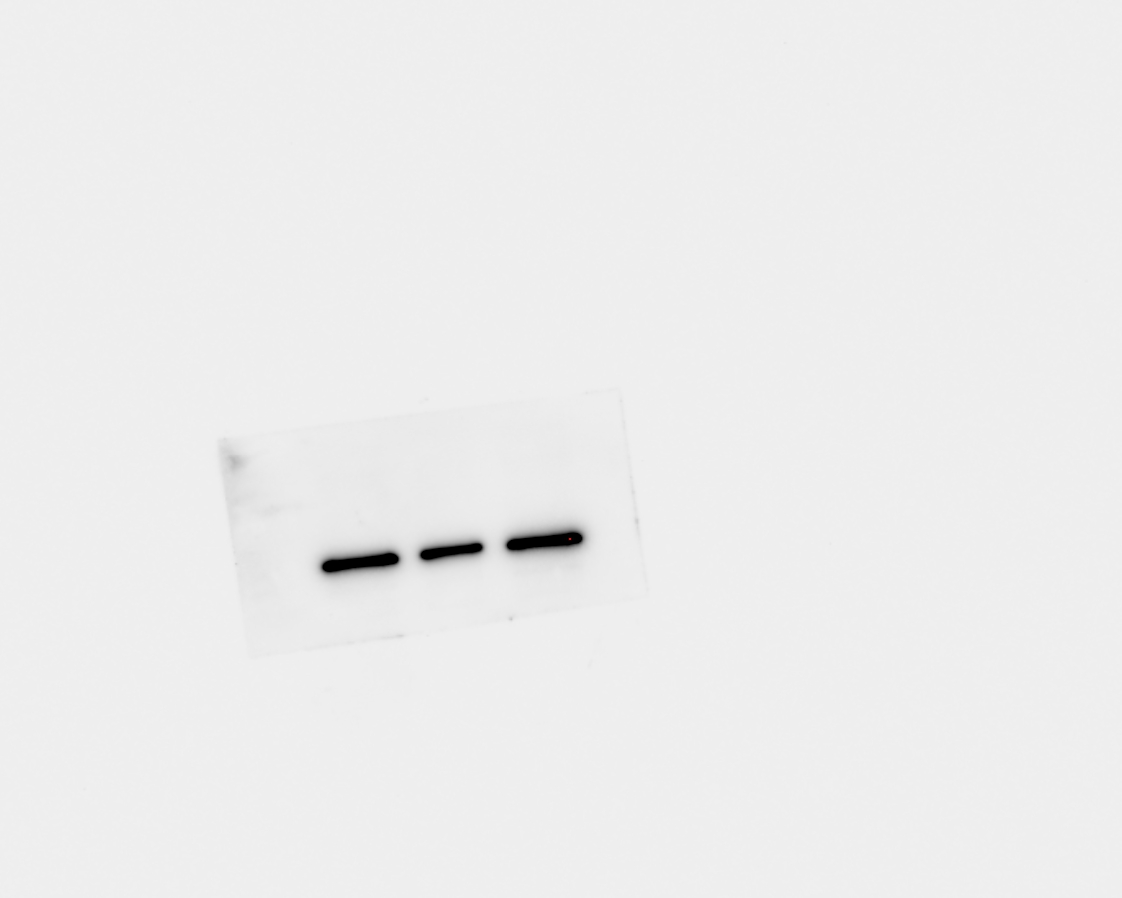

Supplement: Supplementary file 6 [file DataSheet_3.zip › Original Image of Western Blot/8.jpg]

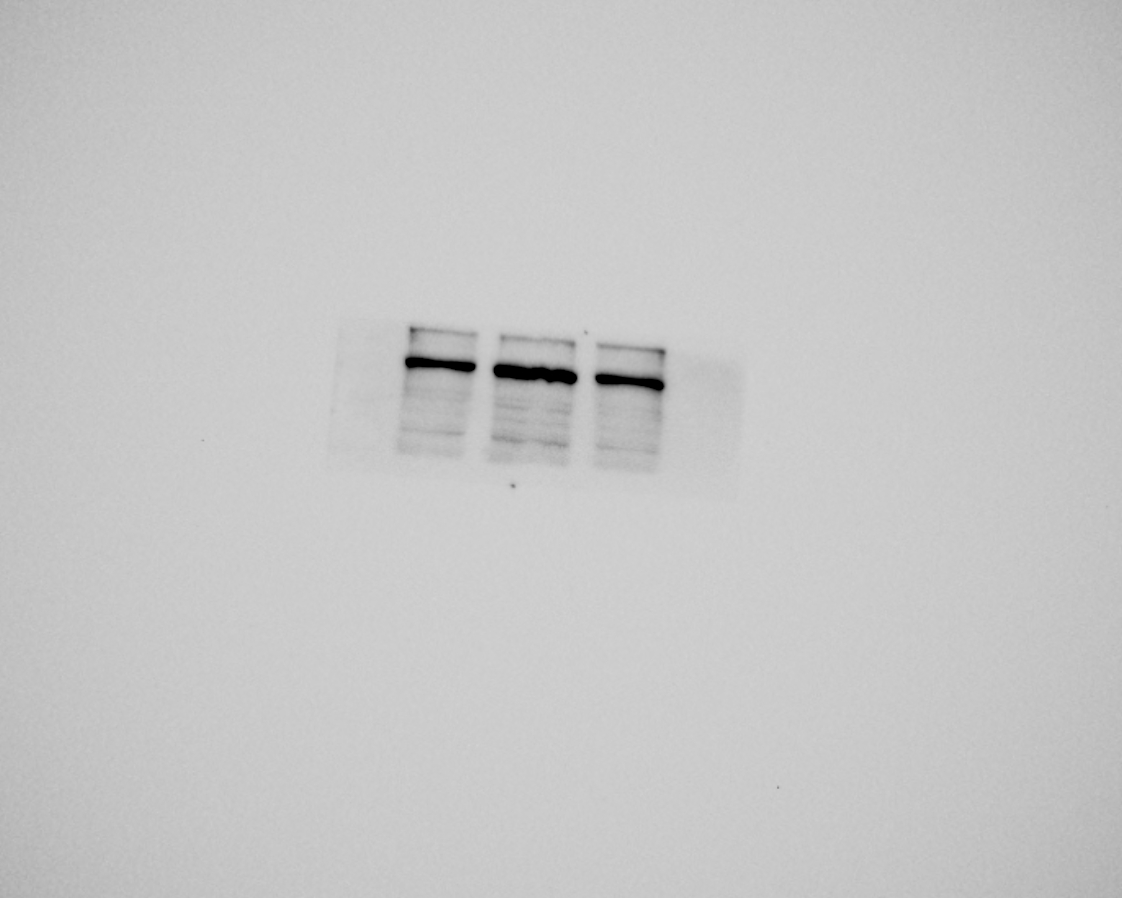

Supplement: Supplementary file 6 [file DataSheet_3.zip › Original Image of Western Blot/9.jpg]
